# Genetic regulators of mineral amount in Nelore cattle muscle predicted by a new co-expression and regulatory impact factor approach

**DOI:** 10.1038/s41598-020-65454-7

**Published:** 2020-05-21

**Authors:** Juliana Afonso, Marina Rufino Salinas Fortes, Antonio Reverter, Wellison Jarles da Silva Diniz, Aline Silva Mello Cesar, Andressa Oliveira de Lima, Juliana Petrini, Marcela M. de Souza, Luiz Lehmann Coutinho, Gerson Barreto Mourão, Adhemar Zerlotini, Caio Fernando Gromboni, Ana Rita Araújo Nogueira, Luciana Correia de Almeida Regitano

**Affiliations:** 1 Department of Evolutionary Genetics and Molecular Biology, Federal University of São Carlos, São Carlos, Brazil; 2 School of Chemistry and Molecular Biosciences, Faculty of Sciences, The University of Queensland, Brisbane, Australia; 3 Agriculture and Food, Commonwealth Scientific and Industrial Research Organisation, Brisbane, Australia; 4 Department of Agroindustry, Food and Nutrition, University of São Paulo/ESALQ, Piracicaba, Brazil; 5 Department of Statistics, Institute of Exact Sciences, Federal University of Alfenas, Alfenas, Brazil; 6 Department of Animal Science, Iowa State University, Ames, IA USA; 7 Department of Animal Science, University of São Paulo/ESALQ, Piracicaba, Brazil; 8 Bioinformatic Multi-user Laboratory, Embrapa Informática Agropecuária, Campinas, São Paulo Brazil; 9 Bahia Federal Institute of Education, Science and Technology, Ilhéus, Brazil; 10 Embrapa Pecuária Sudeste, São Carlos, Brazil

**Keywords:** Genomics, Gene regulation

## Abstract

Mineral contents in bovine muscle can affect meat quality, growth, health, and reproductive traits. To better understand the genetic basis of this phenotype in Nelore (*Bos* indicus) cattle, we analysed genome-wide mRNA and miRNA expression data from 114 muscle samples. The analysis implemented a new application for two complementary algorithms: the partial correlation and information theory (PCIT) and the regulatory impact factor (RIF), in which we included the estimated genomic breeding values (GEBVs) for the phenotypes additionally to the expression levels, originally proposed for these methods. We used PCIT to determine putative regulatory relationships based on significant associations between gene expression and GEBVs for each mineral amount. Then, RIF was adopted to determine the regulatory impact of genes and miRNAs expression over the GEBVs for the mineral amounts. We also investigated over-represented pathways, as well as pieces of evidences from previous studies carried in the same population and in the literature, to determine regulatory genes for the mineral amounts. For example, *NOX1* expression level was positively correlated to Zinc and has been described as Zinc-regulated in humans. Based on our approach, we were able to identify genes, miRNAs and pathways not yet described as underlying mineral amount. The results support the hypothesis that extracellular matrix interactions are the core regulator of mineral amount in muscle cells. Putative regulators described here add information to this hypothesis, expanding the knowledge on molecular relationships between gene expression and minerals.

## Introduction

Besides nutritional quality, mineral amount affects meat quality in many ways. For example, the tenderization process of the skeletal muscle is driven by the action of the calcium-dependent protease calpain^1–4^. Minerals also affect reproduction, as copper, zinc, selenium and manganese supplementation improves pregnancy rate^5^; as well as health and growth performance^6,7^ in beef cattle. Mineral homeostasis regulation partially depends on genetic factors, among others^8^. Thus, understanding the genetic aspects linked to mineral amount in bovine muscle can lead to a better modulation of this trait in Nelore muscle, allowing for future production of healthier, more productive animals, and better-quality meat.

A differential expression approach detected genes and pathways underlying mineral amount in Nelore cattle by comparing extremes of the experimental population used herein^9,10^. However, as mineral amount traits have a continuous distribution trend, it is necessary to study the whole population in order to verify these relationships and infer regulatory modes of action. To go beyond contrasting extreme phenotypes^11^, one can adopt network approaches. For instance, a co-expression network approach allows to identify genome-wide genes with similar expression patterns related to specific phenotypes or conditions. In this methodology, traits are usually integrated into the analysis in a condition-dependent network, by a previous selection of genes or sample clusters related to the trait before the analysis^12^. Another way of including the information of phenotypes to select gene groups putatively involved with them is to cluster all expressed genes by their co-expression profiles and then associate these clusters to the phenotypes using the weighted correlation network analysis (WGCNA) R package^13^. In this analysis, groups of genes with similar functions are identified and associated with the phenotypes, as already described for the mineral amount in our population^14^.

Among the challenges of these methods regarding the inclusion of phenotype information is that no single approach is used to search genome-wide for specific genes linked to phenotypes without prior selection. Also, it is challenging to pinpoint the direction of interactions or the regulation, as co-expression networks do not provide this information a priori^12^. To overcome these limitations, we propose a new application of the partial correlation and information theory (PCIT) algorithm, designed originally for deriving gene co-expression networks through the identification of significant associations between expression profiles^15^, which was applied herein for deriving correlation networks within a matrix of mRNAs, miRNAs and GEBVs for the phenotypes. Additionally, we propose a new application of the regulatory impact factor (RIF) algorithm^16^ to identify genes and miRNAs expression with significant regulatory impact over the GEBVs for mineral amount in bovine muscle. To this end, we tested the impact of mineral associated genes and miRNAs expression values on the GEBVs for minerals, in the same way a transcription factor (TFs) would be tested for its regulatory potential over the expression values of selected genes in the original application. Therefore, we were able to use GEBVs on the networks to identify regulatory elements linked to the phenotypes and, by not relying only on TFs, we allowed the regulatory role to go beyond the current functional annotation of the cattle genome. Herein we describe how this new use of the PCIT-RIF algorithms identified genes and miRNAs whose expression levels in Nelore steers’ *Longissimus thoracis* muscle were correlated to the mass fraction of calcium (Ca), copper (Cu), potassium (K), magnesium (Mg), sodium (Na), phosphorus (P), sulfur (S), selenium (Se), zinc (Zn) and iron (Fe), in the same tissue. We also describe how this information was used to predict the regulatory impact of genes and miRNAs expression over the mineral amount in Nelore muscle.

## Results

### Correlations among genes and miRNAs expression values and minerals

**Figure 1:**
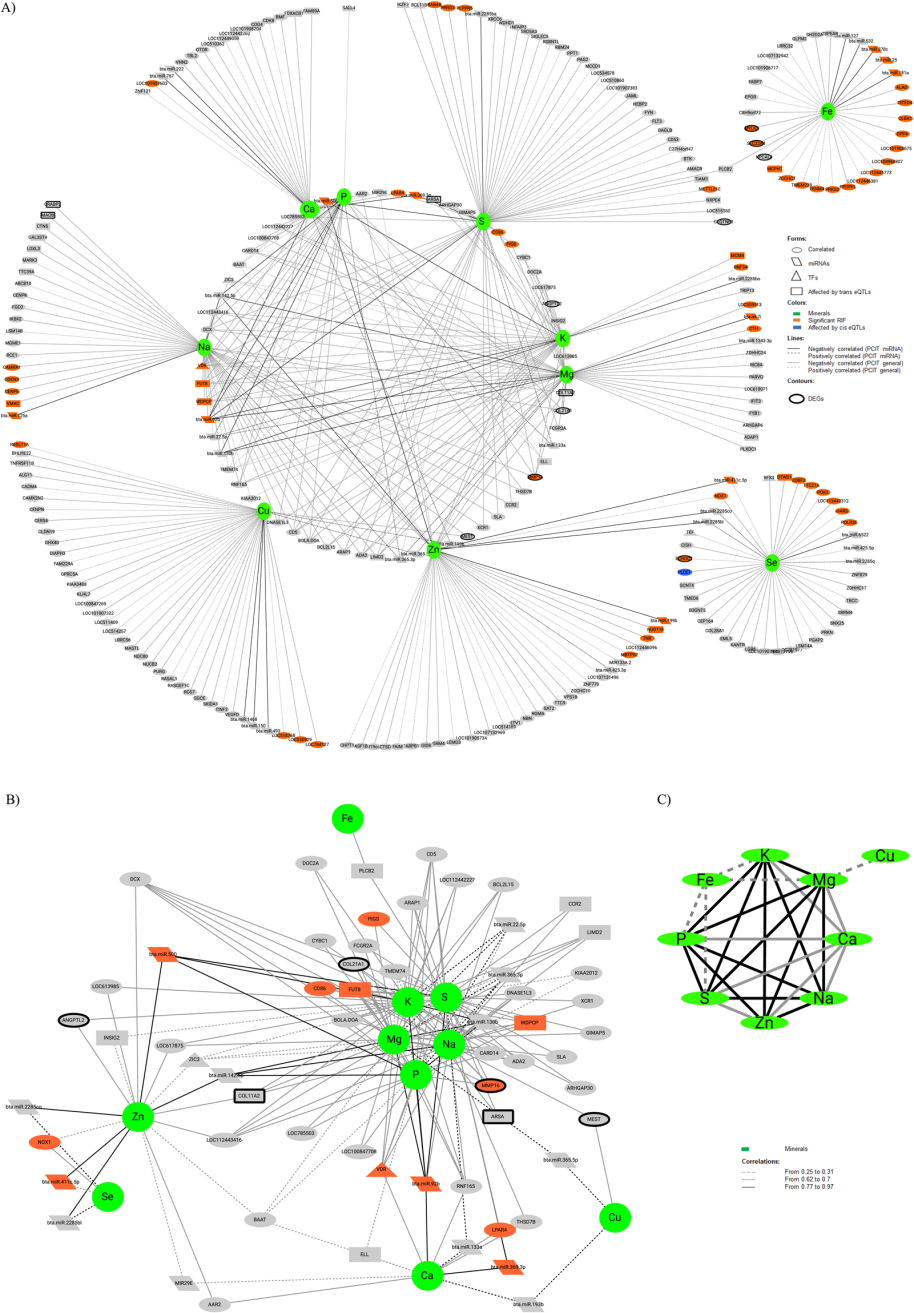
Correlation network among genes and miRNAs with expression values correlated to at least one mineral. This network shows all the significant correlations among genes and miRNAs in the PCIT general and PCIT miRNA analysis. (**A**) Complete network, (**B**) Network with just the correlations regarding the genes and miRNAs with expression values correlated to more than one mineral. It is the internal circle of the complete network with more details, (**C**) Correlations among the mineral’s GEBVs.

The dataset comprehended the expression of 12,943 genes and 705 miRNAs, that remained after data quality control, filtering, normalization and batch effect correction. Simultaneously recognizing the results of both PCIT analyses, PCIT general and PCIT miRNA, we identified a total of 242 genes and 35 miRNAs with expression values correlated to at least one mineral GEBV. From these, the expression of 46 genes and 12 miRNAs were correlated to more than one mineral GEBV. The number of genes and miRNAs with expression values correlated to each mineral ranged from 19 to 55 and from five to nine, respectively. The number of miRNAs whose expression was correlated to a mineral in both PCIT analyses varied from zero to three (Table 1, Fig. 1A). There were two genes and one miRNA expression values correlated to six minerals, being Vitamin D3 receptor (*VDR)* and bta-miR-92b correlated to Ca, K, Mg, Na, P and S; and Doublecortin (*DCX)*, correlated to K, Mg, Na, P, S, and Zn (Fig. 1B). From these analyses, we also identified significant correlations among minerals’ GEBVs. There were no significant correlations between Se and other minerals, while correlations among K, Mg, Na, Zn, S, and P GEBVs ranged from 0.77 to 0.97 (Fig. 1C).

**Table 1:** Number of genes and miRNAs with RNAseq expression values correlated to each mineral amount in *Longissimus thoracis* muscle of Nelore.

Mineral	Gene	miRNA	Repeated miRNA^a^
Ca	22	6	0
Cu	35	5	0
K	33	5	0
Mg	37	8	0
Na	42	6	3
P	19	6	0
S	55	6	1
Se	32	6	2
Zn	36	9	0
Fe	27	5	1

The numbers in the columns Gene and miRNA refer to significant results in at least one PCIT analysis (PCIT general, considering mineral genomic estimates of breeding values, genes and miRNAs expression and PCIT miRNA considering mineral GEBVs and miRNAs expression). ^a^Number of miRNAs with expression values correlated to a mineral in both PCIT analysis (PCIT general and PCIT miRNA).

### Principal component score and Regulatory Impact Factor (RIF)

**Figure 2:**
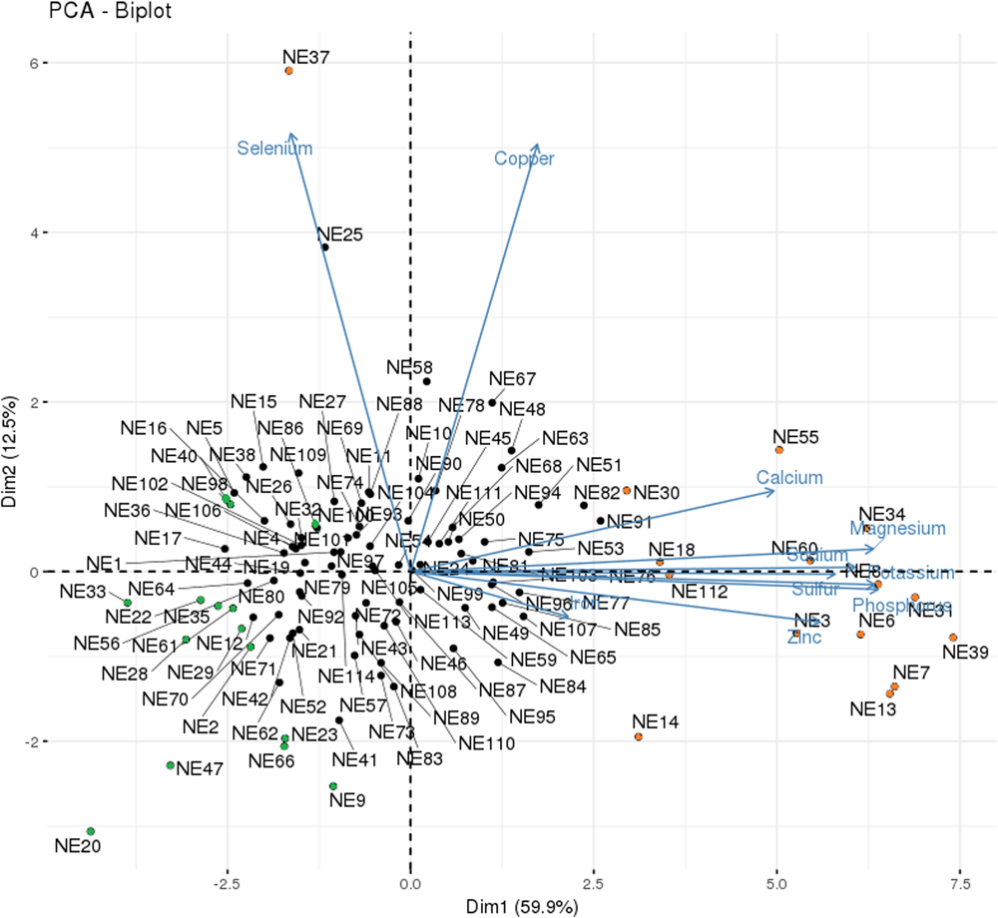
Representation of the contrasting samples considering the genomic estimated breeding values of all 10 minerals together, based on the PCA score. Orange circles represent the samples with the highest scores (positive contrast) and the green circles represent the samples with the lowest scores (negative contrast).

From a principal component analysis based on the GEBVs for each animal, considering ten minerals, we calculated a score for each sample regarding its contribution to phenotypic variation. Based on that, we selected 30 contrasting samples concerning all minerals together, 15 with low score and 15 with high score (Fig. 2). These contrasting groups were used to estimate the RIF in the amount of all minerals together of genes and miRNAs whose expression values correlated to at least one mineral. For these we used our modification of the original RIF algorithm which included genes and miRNAs with expression values correlated to all the minerals, as elements to be tested as regulators and the minerals’ GEBVs as the targets. Additionally, we estimated the RIF of the genes and the miRNAs with expression values correlated to each mineral individually, *i.e*., using contrasting sample groups for each specific mineral. For that, based on the GEBVs, we expanded from six (or five) to 15 the number of samples per contrasting group compared to the detailed in previous works with differentially expressed genes regarding mineral amount^9,10^.

There were 22 genes and two miRNAs with significant RIF based on the high and low general score approach (Table 2). Based on the single mineral analysis, there were three common genes and one common miRNA with significant RIF for two minerals, being CD86 molecule (*CD86)* for K and Mg, *VDR* for Mg and Na, the WD repeat-containing planar cell polarity effector gene (*WDPCP)* for Na and P, as well as bta-miR-369.3p for Ca and S. The number of genes with significant RIFs for each mineral ranged from zero to seven, while for miRNA it ranged from zero to two (Table 2). The RIF values of each significant gene and miRNA by mineral and for the general score analysis are presented in Supplementary Table S1.

**Table 2:** Number of genes and miRNAs with a significant regulatory impact factor over the genomic estimates of breeding values for each mineral and all minerals together (PCA score).

Mineral	Gene	miRNA
Ca	1	1
Cu	4	0
K	3	1
Mg	3	1
Na	6	1
P	1	0
S	5	2
Se	7	0
Zn	4	2
Fe	0	2
PCA Score	22	2

The data came from *Longissimus thoracis* muscle from Nelore steers and the genes and miRNA expressions were identified based on RNA-Seq analysis.

### Correlation network

We used the significant correlations between a gene or a miRNA expression and a given mineral, identified in both analyses implemented with the PCIT algorithm above described, to derive a correlation network. To identify potential regulatory mechanisms related to each mineral, we added on this network other layers of information from the same samples, tissue and experimental population, as follows: differentially expressed genes (DEGs) for contrasting mineral amount sample groups^9,10^, transcription factors (TF)^17^ and genes affected by Expression Quantitative Trait Locus (eQTLs)^18^. This information, along with genes with significant RIFs, was used as node attributes and included in the network analyses (Fig. 1). All correlations and attributes necessary to compose Fig. 1 are provided in the Supplementary Table S2. There was at least one putative regulatory element, *i.e*., a significant RIF, TF, miRNA, or gene affected by eQTLs, correlated to each mineral. The number of genes and of miRNAs with expression values correlated per mineral per attribute is shown in Table 3 and the genes, miRNAs and their attributes are displayed in Supplementary Table S2.

**Table 3:** Number of genes and miRNAs with expression values correlated to mineral amount, per mineral and per matching attribute.

Minerals	DEGs^a^	Significant RIF^b^	TFs^c^	cis eQTLs^d^	trans eQTLs^e^	miRNAs^f^	No attributes^g^
Ca	0	3	2	0	3	5	14
Cu	1	4	1	0	1	5	28
K	2	5	2	0	7	3	19
Mg	2	6	2	0	5	6	23
Na	3	7	2	0	13	6	21
P	0	1	2	0	3	6	12
S	1	8	3	0	8	6	34
Se	1	9	2	1	3	6	17
Zn	0	6	1	0	3	9	27
Fe	3	19	0	0	2	5	9

The numbers result from both PCIT analysis**:** *PCIT general*, with genomic estimates of breeding values (GEBVs) for mineral, genes and miRNAs expression, and *PCIT miRNA*, with only mineral GEBVs and miRNAs expression. Mineral amount, normalized RNAseq obtained gene and miRNA expression levels were from Nelore steers’ *Longissimus thoracis* muscle. Columns represent the number of matches with attributes used for this analysis. ^a^Differentially expressed genes described in refs. ^9,10^. ^b^Genes and miRNAs with significant regulatory impact factor in the present work. ^c^Transcription factors^17^. ^d^Genes affected by cis eQTLs^18^. ^e^Genes affected by trans eQTLs^18^. ^f^Micro RNAs. ^g^Genes and miRNAs with expression values correlated to each mineral that were not identified in previous works.

There were no significant functional clusters or over-represented pathways identified in the functional annotation analysis carried out separately for the genes correlated to a specific mineral. However, from the functional annotation table, we noted that the genes with expression correlated to the minerals are well conserved among a broad range of organisms. These genes have functions related to the extracellular matrix, integral membrane constituents, metal ion binding. They participate on regulatory processes linked to transcription, replication, splicing, apoptotic processes, metabolism, transport vesicles, RNA processing, signalling, cell division, adhesion, migration and proliferation, embryonic development and tissue regeneration.

### Integration with differentially expressed genes (DEGs)

In order to convey the relationship among all genetic elements related to mineral mass fraction detected in our population, we used PCIT. In this analysis we estimated the correlations between the expression of a gene or miRNA, which was found to be correlated to a mineral in the present work, and DEGs previously identified for the same mineral^9,10^. This analysis was carried out for each mineral separately and included the same genes with regulatory potential as in the previous section (DEGs^9,10^, TFs^17^, genes affected by eQTLs^18^ and genes with significant RIF). To identify elements with regulatory potential, we then selected the genes that were network hubs or that were significant according to RIF (see methods). We performed a functional annotation analysis with the selected genes for each mineral, separately, to determine which ones were underlying biological pathways.

**Figure 3:**
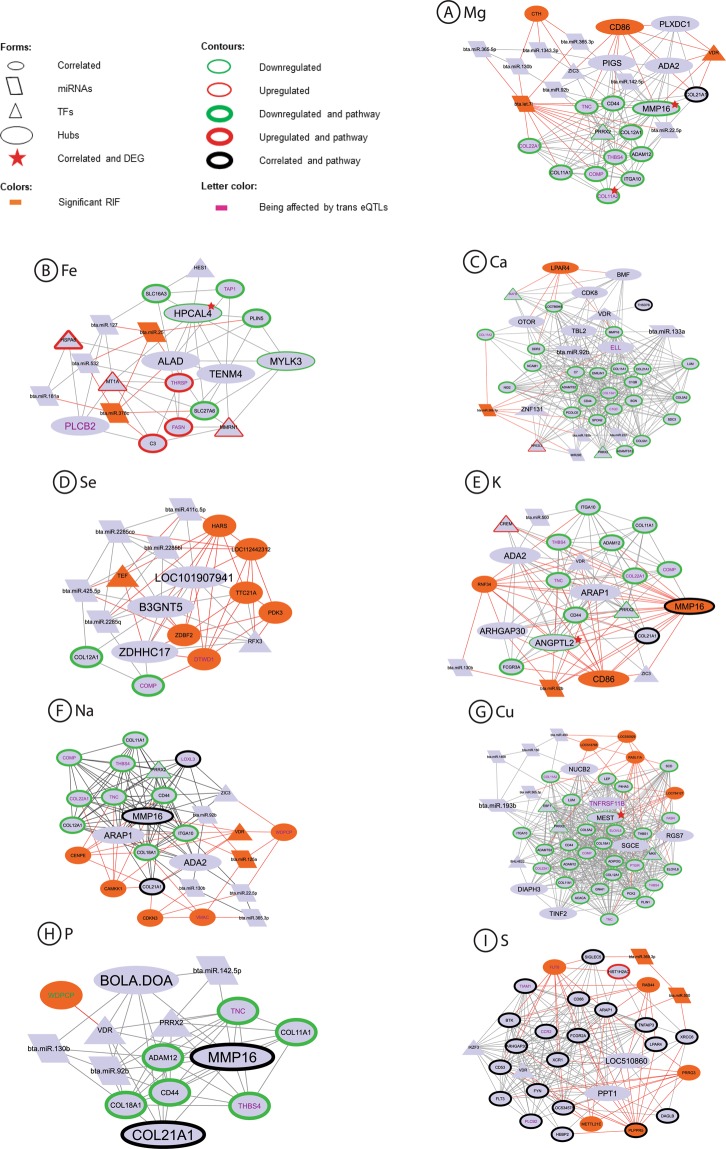
Co-expression networks among genes and miRNAs being part of enriched pathways (DEGs and correlated to a mineral), hubs, TFs, miRNAs or presenting a significant RIF regarding nine of the minerals in study. (**A**) Mg, (**B**) Fe, (**C**) Ca, (**D**) Se, (**E**) K, (**F**) Na, (**G**) Cu, (**H**) P, (**I**) S. Red lines represent the correlations with a significant RIF gene or miRNA.

The expression of all selected putative regulatory elements (hub, significant RIF or miRNA), the ones underlying newly identified biological pathways and the ones being part of enriched pathways in previous work with DEGs related to mineral amount^9,10^, were used as inputs for a final PCIT analyses. This PCIT was carried to identify possible regulators of genes in enriched pathways. Figure 3 shows the co-expression networks built with significant correlations from the final PCIT analyses for Ca, Cu, K, Mg, Na, P, S, Se, and Fe. Supplementary Tables S3 has the correlations and attributes used for creating Fig. 3.

As we included the differentially expressed genes regarding mineral amount previously detected in the same population^9,10^, most of the over-represented pathways identified correspond to the previously detected pathways. Besides, by the inclusion of correlated genes and pathways from the Reactome database^19^, we identified new pathways for K, related to protein metabolism, as well as for Ca, Cu, S and Fe, related to immune response, and for S related to signalling. All the pathways enriched for S are new, when compared with our previous work^9^. A list of the pathways enriched for each mineral, considering both the ones detected with the inclusion of correlated genes expressions and the ones from the previous work^9,10^, is shown in Table 4.

**Table 4:** Pathways enriched for each mineral considering the gene expressions correlated to each one of them and the previously detected differentially expressed genes related to the same minerals in the same Nelore population.

	Ca	Cu	K	Mg	Na	P	S	Se	Fe
AMPK signalling pathway		3							
Antigen processing and presentation									1
Assembly of collagen fibrils and other multimeric structures									
Biosynthesis of unsaturated fatty acids		1							
Collagen biosynthesis and modifying enzymes	2	2							
Collagen chain trimerization	2	2		2	2				
Collagen formation					2				
DAP12 interactions							2		
Degradation of the ECM	2								
ECM organization	2	2	2	2	2	2			
ECM-receptor interaction	1	3	3	3	3	1		1	
Fatty acid biosynthesis		3							
Fatty acid metabolism		3							
Fc gamma receptor (FCGR) dependent phagocytosis							2		
Focal adhesion		1	1	1	1	1			
G alpha (q) signalling events							2		
Herpes simplex infection									1
Immune system							2		
Influenza A									1
Innate immune system							2		
Integrin cell surface interaction	2	2			2				
Measles									1
Neutrophil degranulation							2		
Non-integrin membrane-ECM interactions	2								
O-glycosylation of TSR domain-containing proteins	2								
Phagosome			1						
PI3K-Akt signalling pathway		1	1	1	1	1			
Platelet activation		1							
PPAR signalling pathway		1							1
Prion disease	3								
Protein digestion and absorption	1	3		3	3	3		1	
Signal transduction							2		

Pathways just enriched in previous works with a differential expression approach and the same Nelore population are represented by the number 1, pathways enriched in our correlated genes expression are represented by the number 2 and the pathways enriched both in previous work and in the correlated genes expressions are represented by the number 3. There were no enriched pathways for Zn.

**Figure 4:**
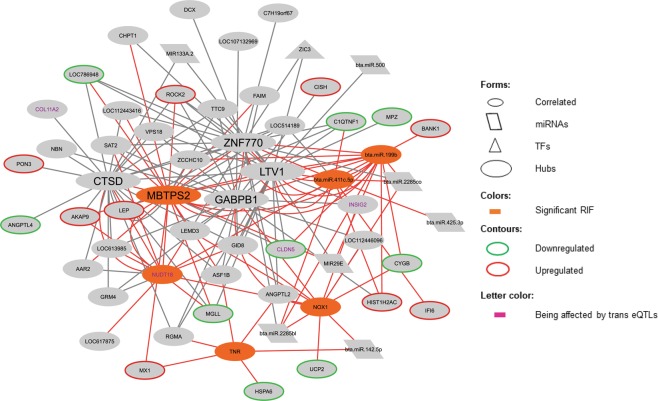
Co-expression network containing DEGs for Zn, genes or miRNAs with expression values that are correlated to these DEGs and are also a hub or a significant RIF for Zn, ora miRNA correlated to Zn. Their functional attributes are presented in different colors or shapes. Red lines represent the correlations with a significant RIF gene or miRNA. This network is presented in separate for the others in Fig. 3 because there are no DEGs for Zn in the network taking part of enriched pathways.

However, no gene taking part in the unique enriched pathway previously detected for Zn^9^ met our criteria. Because of that, we generated a co-expression network by including the DEGs for Zn^9^ that had their expression values significantly correlated to hub or RIF elements for this mineral and their attributes, in order to identify possible regulators for the DEGs found in contrasting Zn samples. This co-expression network is shown in Fig. 4, and the correlations and attributes supporting this network are presented in Supplementary Table S4.

## Discussion

### Relationship among minerals

Correlations identified among GEBVs for most minerals were high (0.77 to 0.97). Thus, a word of caution must inform this discussion of all genes and miRNAs with expression values correlated to each mineral, as correlated responses across minerals may underlie the identified genes and miRNAs, as well as their predicted relationships. In our correlation network, the link between Se and the other minerals was Zn, through the common correlation with the NADPH oxidase 1 (*NOX1)* gene expression, which had significant RIF only for Zn. *NOX1* expression was positively correlated to Zn and negatively to Se. Accordingly, Zn accumulation in the human mitochondria increases the production of reactive oxygen species by the *NOX1* encoded NADPH protein, which in turn activates NF-Kb, a known positive transcriptional regulator of *NOX1*, thus increasing its expression. Conversely, Se acts through selenoproteins, most of which have redox properties. Its deficiency is known to support the oxidation of both Keap1 and Nrx oxidation sensors^20^ in the presence of H_2_O_2_. The oxidation of NrX protein leads to the activation of the Wnt signalling pathway^20^, which can act in adult muscle regeneration^21^, hence adding evidence for the relevance of this regulation for muscle homeostasis. Thus, both the Zn induced accumulation of H_2_O_2_. and the oxidation induced by Se deficiency have similar effects on the same known biochemical process, which is known to impact many production and reproductive traits, such as residual feed intake^22^ and spermatic morphology^23^, indicating that this relationship between this minerals and redox reactions can be useful in selection programs. Another link between Se and Zn were the common correlations with the expression of three miRNAs: bta-miR-411c-5p (with significant RIF for Zn), bta-miR-2285co and bta-miR-2285bl, although no literature relates these miRNAs to Se or Zn amount.

Fe showed a weak correlation with Mg, K, P, and S and was linked to other minerals through S, sharing negative correlations with the expression of Phospholipase C Beta 2 gene (*PLCB2)*. The protein coded by this gene is critical to Ca efflux^24^, although no correlation with Ca amount was found in our data, nor in our previously reported DEGs^9^. The relationship of *PLCB2* gene expression with Fe and S is undocumented, although Fe was reported to cleave the Phospholipase C Beta 2 protein in the cornea of bovine, porcine and humans^25^. The *PLCB2* gene is affected by 61 trans eQTLs, harboured across 12 chromossomes^18^, making these eQTL regions candidates for regulating this gene expression and consequently Fe and S mass fractions in the muscle.

### PCA score analyses identified regulators of mineral composition

Our score successfully detected contrasting samples regarding all minerals together, allowing for the identification of genes and miRNAs with significant overall RIFs. Considering these genes and the functional enrichment analysis, we identified functions conserved across several species for 14 out of 22 genes. From these, we can highlight three with functions related to minerals pointed in DAVID annotation chart: Delta-aminolaevulinic acid dehydratase (*ALAD)* encodes a metal ion binding protein linked to Zn while Zinc finger CCHC domains-containing protein 7 (*ZCCHC7)* encodes a Zn finger chaperone protein, and Myosin light chain kinase 3 (*MYLK3)* is part of the Ca signalling pathway that participates in muscle contraction.

Mutations in the *ALAD* gene were linked to the phenotypic expression of potentially toxic metal by fly ash exposure in cattle born near thermal power plants, being pointed as a candidate for genomic studies related to metal toxicity^26^. Our results indicated that *ALAD* is a candidate linked to minerals in general, including potentially toxic metals.

### Functional analyses and search for regulatory elements

Functional annotation analyses, based on the genes with expression values correlated to each mineral, showed no functional clusters nor enriched pathways for any mineral. However, some of these genes had their expression correlated with DEGs partaking in different pathways in which part of the mineral-correlated genes themselves also figure as members. This led us to hypothesize that the genes that are found in enriched pathways but do not present its expression correlated to minerals may be modulated in less intensity. This agrees with the small Quantitative Trait Loci (QTL) effects already observed for mineral amount^27^. The function annotation for each gene separately indicated membrane proteins and extracellular matrix (ECM) related proteins as common annotations for many genes. This observation helps to corroborate the hypothesis that ECM interactions are at the regulatory core for the mineral mass fraction^9^. ECM pathways were enriched for co-expressed groups of genes related to mineral mass fraction and muscle metabolism in this Nelore population^14^.

When components of a specific pathway are known, a guided-gene approach in a co-expression network can help to identify new genes for the same pathway-related-trait^28^, and a pre-selection of genes by biological meaning can improve the network interpretation^12^. Our selection based on enriched pathways, TFs, and significant RIF allowed the inference of genes and miRNAs with a regulatory potential in these pathways. We identified high correlations among these selected elements when compared with the correlations among unselected genes/miRNAs and minerals or when considering all genes/miRNAs correlated to a mineral and their respective DEGs. These high correlations and the presence of genes related to regulatory processes reinforces that our methodology can be used to drive the search for meaningful regulatory relationships.

### Potential regulators for more than one mineral

Genes with significant RIF and genes with expression values correlated to other genes that belong to enriched pathways were considered as the potential regulators. These candidate genes may modulate mineral mass fraction by affecting their target genes and pathways. For the minerals presenting enriched pathways, except Zn, the elements with significant RIFs were connected to miRNAs, correlated genes expressions, TFs and to genes being affected by trans eQTLs. They were also part of enriched pathways, reinforcing their regulatory role on the phenotypes. The intricate patterns obtained in these network analyses arise from the fact that the same genes are part of different pathways.

As expected, the pathways identified by considering gene expression correlation with mineral GEBVs were often the same already reported in the differential expression study^9^. These results also corroborate our previous hypothesis that the regulatory core of mineral amount is linked to ECM processes^9^. Pathways related to fatty acid metabolism were enriched for Cu, as also reported in that previous study. However, with the inclusion of the genes with expression values correlated with the minerals, pathways linked to immune responses were enriched for Ca, Cu, Fe, and S. The pathways enriched for S, related to signal transduction and immune response, were not detected before, emphasizing that the integrative approach used here can bring up new evidence of regulatory processes not identified under the differential expression analysis.

Most of the putative regulators are correlated to or take part in ECM and immune system-related pathways, as well as fatty acid metabolism-related pathways, as is the case of Cu. ECM is related to the development of skeletal muscle by providing biomechanical strength of the intramuscular connective tissue and regulating muscle cell behaviour. It also is implicated in post-mortem meat aging, as changes in the ECM reduce the strength of the intramuscular connective tissue, contributing to the tenderization of the meat. Additionally, the ECM is linked to fat deposition, once during fattening there is a remodelling of the ECM, reducing the mechanical strength of intramuscular connective tissue^29^. High fatty deposits are linked to inflammation, which links Cu to the immune system-related pathways. Thus, our results may be useful to derive nutritional strategies to improve the performance for fat deposition and beef tenderness.

We identified putative regulators that might impact more than one mineral, which were linked to ECM, immune system or fatty acid related pathways as well. The cluster of differentiation 86 gene (*CD86)* showed a significant RIF and was a hub gene for Mg and K analyses. *CD86* encodes a signalling protein for T cell activation and proliferation^30^ and is linked to T cell adhesion after activation^31^. Another putative regulator, the Mg sensor ITK, seems to be required for optimal T cell activation^32^ after the binding of the CD86 protein in the CD28 receptor^33^. This process involves K^+^ channels, putatively explaining the relationship among these two minerals and *CD86*. The PI3k-akt signalling pathway is activated after CD86 protein binds to the CD86 receptor in an antigen-presenting cell, leading to downregulation of integrins, components of the ECM^34^.

The Vitamin D receptor (*VDR*) was a TF with significant RIF for Mg and Na. The relationship between this gene and the ECM processes-related pathways that were enriched for both minerals seems to be the interaction of the VDR receptor with the Runx2 receptor. In mammals, this interaction stabilizes chromatin remodelers by activating genes involved in ECM mineralization^35^. Mg is essential to vitamin D activation, once both enzymes involved in this process, 25-hydroxylase and 1α-hydroxylase, are Mg-dependent^36^. Nonetheless, the link between *VDR* expression and Na is not extensively documented.

WD repeat-containing planar cell polarity effector (*WDPCP)* gene showed significant RIF for Na and P and was affected by one trans eQTL in chromosome five^18^. This gene encodes a protein that inhibits Wnt activity^37^. Wnt pathway acts in adult muscle regeneration^21^ and is activated by high P amounts^38^. ECM processes-related pathways were also enriched for these minerals. ECM stiffness increases the expression of several members of the Wnt pathway through integrins and focal adhesion pathways^39^, thus relating the *WDPCP* gene expression with the ECM. The link between *WDPCP* expression and Na is not known. In both minerals, Na and P, *WDPCP* expression value is positively correlated (0.19) with the TF *VDR* expression that represses the Wnt pathway^40^. Increased expression of this pathway is involved with increased inflammatory response after tick infestation in the skin of Bradford steers^41^, suggesting that Na and P can be candidates to more studies linking them to the indication of tick resistance.

The miRNA bta-miR-369-3p had a significant RIF for Ca and S and was correlated to several genes involved in immune pathways for Ca and S. The genes with expression values correlated to this miRNA are not known targets to it. This miRNA expression level is increased in skin and serum of humans with psoriasis^42^, which trigger seems to be the activation of the cellular immune system^43^. A homolog of psoriasin, a common protein in psoriasis patients, was identified in bovines and had the same antimicrobial and immune response activity as the human protein^44^. Further, Ca and vitamin D play important roles in keratinocyte differentiation and regulate proteins involved in psoriasis^45^ and S is used as a known treatment and prevention of recurrence for this disease^46^. Our results suggest the genes whose expressions were correlated to bta-miR-369-3p expression as new candidate targets of this miRNA, linked to immune response and mineral concentration.

### Potential regulators for a specific mineral concentration

Some putative regulators showed significant RIF for only one mineral, linked to the same important pathways. The miRNA bta-let-7i showed significant RIF for Mg and one of the genes correlated to this miRNA in the Mg analysis, Collagen alpha-1 (XI) chain (COL11A1) is a target of this miRNA. The *COL11A1* gene is associated to protein digestion and absorption, as well as to ECM receptor interaction. This gene encodes a collagen protein, the most abundant protein in ECM. *COL11A1* expression is correlated to Mg, which stimulates collagen synthesis^47^, and its expression is correlated to the expression of other genes of the same or related pathways. Cystathionine gamma-lyase (*CTH*) is also a gene with significant RIF only for Mg. This gene expression is correlated to a Zn finger protein of the cerebellum (ZIC3), a TF, which was correlated to the already mentioned *CD86* gene expression, also associated with Mg and ECM herein.

We identified two genes with significant RIF, specifically for K: Matrix metallopeptidase (*MMP16)* and E3 ubiquitin-protein ligase (*RNF34)*. The gene *MMP16* encodes a protein whose family is involved in the breakdown of ECM, mostly by degrading collagen proteins^48^. This function could explain the enriched pathways related to ECM organization and its correlation with Collagen type XXI alpha 1 chain (*COL21A1)*. Both *MMP16* and *RNF34* expressions were correlated to *CD86* expression, for which the link to K was already discussed. *RNF34* encodes a RINF finger protein that negatively regulates the NOD1 pathway, involved in receptors that activate immune responses similarly to *CD86*. Bta-miR-92b expression was correlated to the expression of seven genes associated with ECM and immune system-related pathways. One of these genes, *MMP16*, being a known target for this miRNA, which could explain the relationship of this miRNA with the over-represented pathways.

For Na, we identified six genes with significant RIF: *WDPCP* and *VDR*, linked to the already discussed ECM processes, Vimentin type intermediate filament associated coiled-coil protein (*VMAC)*, Cyclin-dependent kinase inhibitor 3 (*CDKN3)*, Centromere protein E (*CENPE)*, and Calcium/calmodulin-dependent protein kinase 1 (*CAMKK1)*. VMAC plays an important role in cytoskeletal organization^49^. Cell adhesion, mediated by integrins, connect ECM and cytoskeleton^50^. *CDKN3* encodes a cyclin-dependent kinase inhibitor that is involved in cell cycle regulation^51^, a process where integrins take part^52^. The presence of an integrin gene, integrin subunit alpha 10 (*ITGA10)* in the network, as well as actin interactions, could explain the link between these two genes and the ECM-related pathways for Na. Na presented a miRNA with significant RIF, bta-miR-125a, with expression values correlated to two genes with significant RIF, *WDPCP* and *VMAC*, as well as with the integrin gene *ITGA10*. This miRNA targets *VMAC* which is also affected by six trans eQTLs in chromosome six, being candidate for future studies on the regulation of this gene.

The miRNAs bta-miR-25 and bta-miR-378c had significant RIF for Fe. Their expression values were correlated to each other and to other miRNAs expression. As with other miRNA found in our results, the genes whose expressions correlated to bta-miR-25 and bta-miR-378c were not described as their targets. Both miRNAs expressions were correlated to the expression of the hub gene *ALAD* in the Fe network. The levels and activity of Delta-aminolevulinic acid dehydratase, the *ALAD* gene encoded protein, are positively affected by the Fe amount in the extracellular environment^53^. The relationship with the immune response pathways enriched for Fe seems to be through the proteasome. Delta-aminolevulinic acid dehydratase protein modulates proteasome activity^54^, which is known to shape innate and adaptative immune responses^55^.

Lysophosphatidic acid receptor 4 (*LPAR4)* was a hub gene with significant RIF for Ca. This gene product is known to positively regulate cytosolic Ca amount through the phospholipase C-activating G protein-coupled signalling pathway (GO:0051482). Its expression was linked in our network to MAF BZIP transcription factor B (*MAFB)* expression, a TF that interacts with Gcm2 and modulates the expression of the parathyroid hormone encoding gene (*PTH*), which in turn regulates Ca mass fraction^56^. The expression of this genes was correlated to other six genes. Three of them were DEGs for Ca being part of pathways involved in ECM processes, and the other three were hub genes. From these hub genes, Bcl-2-modifying factor (*BMF)* regulates apoptosis after cell detachment from the ECM^57^.

We identified the RAS like family 11 member A gene (*RASL11A)*, which encodes a RAS protein, with significant RIF for Cu. This gene expression was positively correlated mainly to the expression of genes involved in fatty acid metabolism, a process where Cu is a known enzymatic co-factor^58^. RAS proteins’ posttranslational modifications are affected by fatty acids^59^, thus suggesting a feed-back mechanism regulating this gene.

For S, we identified Fucosyltransferase 8 (*FUT8)*, RAB44 member RAS oncogene family (*RAB44)*, Proline-rich and gla domain 3 (*PRRG3)*, Protein-lysine methyltransferase METTL21E (*METTL21E)*, and Phospholipid phosphatase related 5 (*PLPPR5)* genes with significant RIF, being their expression values correlated or being part of immune response and signal transduction pathways. Sulfur amino acids affect the inflammatory aspects of the immune system^60^. Although there is no primary connection between *FUT8* and *RAB44* proteins and the immune system, these proteins contribute to tumor progression^61,62^, in which a robust immune response is involved^63^. *PRRG3* encodes a vitamin K-dependent transmembrane protein with a GLA domain involved in coagulation factors^64^, a process related to the innate immune system^65^. Regarding signal transduction pathways, *METTL21E* was linked to signalling pathways in mouse siRNA experiments^66^, and *PLPPR5* encodes a protein member of the phosphatidic acid phosphatase family, acting in phospholipase D mediating signalling^67^. The bta-miR-500, who presented a significant RIF for S is a known regulator of the genes whose mRNA levels were correlated to this miRNA in our analysis.

For Se, all enriched pathways were related to ECM interactions as well as to protein digestion and absorption. For this mineral, we identified six annotated genes with significant RIF, Thyrotroph embryonic factor (*TEF)*, Zn finger DBF-type containing 2 (*ZDBF2)*, Tetratricopeptide repeat domain 21 (*TTC21A)*, Histidyl-tRNA synthetase (*HARS)*, DTW domain containing 1 (*DTWD1)*, and Pyruvate dehydrogenase kinase 3 (*PDK3)*. *TEF* is a TF and a leucine zipper protein^68^, whose family is required for the activation of DDRs receptors, essential to matrix remodeling^69^. *PDK3* encodes an enzyme responsible for the regulation of glucose metabolism and, among many other functions, is related to ECM remodeling^70^. We could not find a link among *ZDBF2*, *HASR*, and *DTWD1* genes expression and Se or the enriched pathways. Thus, they are candidates for future studies regarding these potential relationships.

Regarding Zn, even without over-represented pathways, it is possible to infer that the six elements presenting significant RIF are putative regulators of several genes with correlated expressions and of a few DEGs, as already discussed by *NOX1*. From the six genes with significant RIF, the Membrane-bound transcription factor peptidase, site 2 (*MBTPS2)* gene, which encodes an intramembrane Zn metalloprotease, was also a hub gene, whereas *TNR* encodes an ECM glycoprotein. This information can lead to the assumption that the ECM processes can also be associated with Zn amount, as they putatively do to most of the other minerals in this study^9^.

### New application for PCIT and RIF algorithms

The first co-expression network, containing gene and miRNA expressions correlated to the mass fraction of at least one mineral, is considered to be a correlation network among elements from two different sources: sequencing (mRNA-Seq and miRNA-Seq) and a measure referring to the trait of interest, the minerals‘ GEBVs. Originally, outputs from PCIT algorithm is used to build co-expression networks based on significant correlations between gene and miRNA expression levels. However, in theory PCIT can be used to test the correlation and the significance threshold of other genetic elements, if they vary in the population. Thus, there is no statistical impediment of using PCIT in the way we proposed here, to detect genes and miRNAs whose expression values vary in the experimental samples in correlation with the minerals’ GEBVs, since GEBVs represent only the additive genetic effect of the traits^27^, thus being a genetic element as well.

The RIF algorithm was developed to calculate the impact of TFs over a selected list of genes through the expression values of genes and TFs across samples, in two contrasting groups for the studied phenotype (in our case, minerals). Again, there is no impediment in the analytical method to use other genetic information, *e.g*., GEBVs, since it varies in the population. In our application, RIF gives a high score to the genes or miRNAs that are most differentially co-expressed, highly abundant and with more expression difference between the contrasting groups (mineral specific groups and score-based groups, separately) and to genes and miRNAs for which the expression can predict better the magnitude of the GEBVs. Together, both new applications can be used to predict the correlation between genes or miRNAs expressions and the genetic estimated value for mineral mass fraction, thus highlighting genetic elements with regulatory impact over mineral amount. Besides adding information to the understanding of mineral regulation, our findings might also contribute to SNP prioritization for genomic selection aiming these traits, if the effect of sequence variants in the regulatory candidate genes is verified.

## Conclusion

By using a modification of the PCIT/RIF methodology, we were able to predict regulatory elements related to the mineral amount of ten minerals, indicating over-represented pathways related to the mass fraction of each mineral and putative regulators that are mineral-specific. Our analyses corroborate the link between mineral amounts and the ECM processes, including for Zn, which was not seen in our previous analysis. In our proposed approach, PCIT can be applied to predict the relationship between gene transcripts, or miRNAs, and phenotypes, in a genome-wide fashion. Similarly, RIF may predict the regulatory impact of mRNA and miRNA levels over phenotypes. This new approach can be applied for any phenotype that is of interest for genomic selection and livestock breeding.

## Methods

### Samples

**Figure 5:**
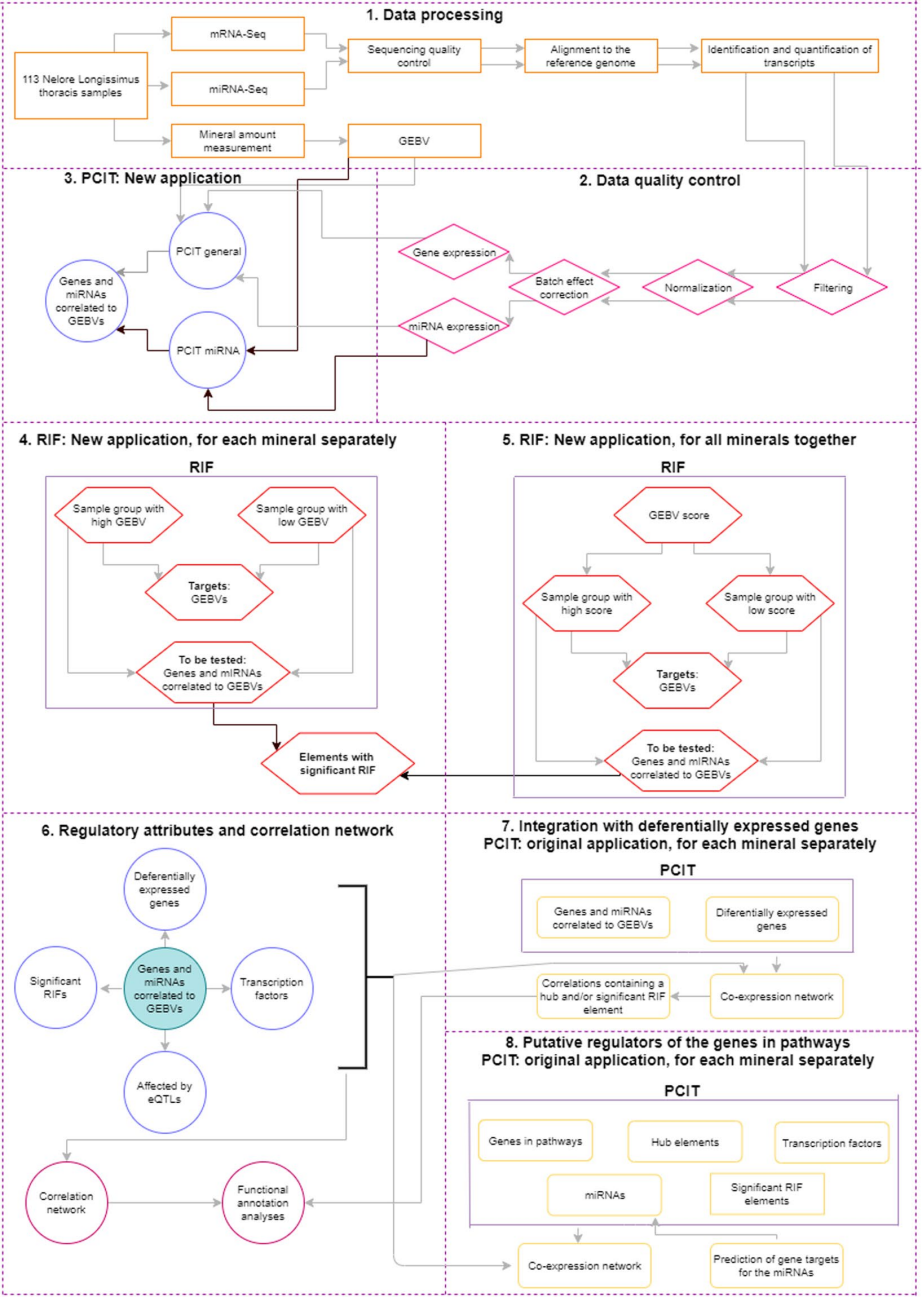
Flowchart representing the steps of the methodology.

The Ethical Committee of Embrapa Pecuária Sudeste (São Carlos, São Paulo, Brazil) approved all experimental, animal protocols and methods (CEUA 01/2013). All methods were performed in accordance with the relevant guidelines and regulations. We used the GEBVs from mineral amount^27^, the mRNA-Seq^10^. and miRNA-Seq^71^. data from 113 samples of *Longissimus thoracis* muscle from Nelore steers, part of the population already described in differential expression analysis related to mineral amount^9,10^. Figure 5 contains the steps of our methodology.

The samples studied here are a subsample from a Nelore steers population described elsewhere^27,72^. In summary, all animals belong to half-sibling families and our samples were generated by artificial insemination in two different farms, during two breeding seasons. They were transferred to a feedlot system at Embrapa Pecuária Sudeste (São Carlos, São Paulo, Brazil) with an average of 21 months of age and mantained in the feedlot with *ad libitum* feed and water access until slaughter. The slaughters were organized in nine different groups, when the animals were in average 25.5 months old, approximately 70 days after the feedlot started. A cross-section of the *Longissimus thoracis* muscle samples were collected in the slaughterhouse right after the exsanguination, using a 5 mm hole saw, between the 11^th^ and 13^th^ ribs, and conserved in liquid nitrogen until RNA extraction.

### Mineral mass fraction and genetic estimated breeding value (GEBV)

Calcium (Ca), copper (Cu), potassium (K), magnesium (Mg), sodium (Na), phosphorus (P), sulfur (S), selenium (Se), zinc (Zn) and iron (Fe) mass fractions were determined from lyophilized and microwave-assisted digested samples, such as described elsewhere^27^. Calcium, Cu, K, Mg, Na, P, S, Zn, and Fe were determined by inductively coupled plasma optical spectrometry (ICP OES, Vista Pro-CCD with a radial view, Varian, Mulgrave, Australia). Selenium was determined by inductively coupled plasma mass spectrometry (ICP-MS 820-MS, Varian, Mulgrave, Australia).

The genetic breeding values (GEBVs) for all the minerals’ amount were previously estimated^27^ through a Bayesian model that considered birthplace, feedlot location and breeding season in the contemporary groups as fixed effects and age at slaughter as a linear covariate.

### mRNA-Seq and miRNA-Seq sequencing and quality control

The total RNA extraction, quality control, and sequencing were described elsewhere^71^. In summary, total RNA from all samples was extracted using Trizol^®^ (Life Technologies, Carlsbad, CA) and its integrity was evaluated in a Bioanalyzer 2100^®^ (Agilent, Santa Clara, CA, USA). Regarding the mRNA-Seq data, the library preparation was made with the TruSeq^®^ sample preparation kit, and the paired-end sequencing^10^ was made in an Illumina HiSeq. 2500^®^. For the miRNA-Seq data, the library preparation was made with TruSeq^®^ small RNA sample preparation kit, and the single-end sequencing^71^ was made in a MiSeq^®^ sequencer.

As a quality control for the sequences, we filtered out reads with less than 65 bp and Phred Score less than 24 for the mRNA-Seq data, and with less than 18 bp and Phred Score less than 28 for the miRNA-Seq data, using the Seqyclean software (http://sourceforge.net/projects/seqclean/files/).

The reads that passed the quality control were aligned to the reference bovine genome ARS-UCD 1.2 with the STAR v.2.5.4 software^73^ for the mRNA-Seq data and with the mirDeep2 software^74^ for the miRNA-Seq. The same software was used for the identification and quantification of transcripts and miRNAs, respectively, in raw counts.

### Filtering, normalization and batch effect correction

After quality control, the mRNA-Seq and miRNA-Seq expression data were filtered separately to remove the transcripts and miRNA not expressed in at least 22 samples, or approximately 20% of the samples.

A first component analysis was performed for the mRNA-Seq expression data with the NOISeq v.2.16.0 software^75^ to visually verify the batch effect of the birthplace, feedlot location, breeding season, age at slaughter, slaughter group and a combination of sequencing flowcell and lane over the expression data. The data were normalized using the VST function from DESEq. 2 software^76^, and the batch effect correction for the combination of sequencing flowcell and lane was made using the ARSyNseq function from NOISeq v.2.16.0 software^75^. For the miRNA-Seq data, the procedure was the same, with the batch effect correction being applied only for the sequencing lane.

### PCIT (Partial Correlation Coefficient with Information Theory) among mRNA, miRNA and phenotypes

A new application of the PCIT algorithm^15^ was developed to test the correlation among the expression values of mRNAs and miRNAs that passed the quality control filters as well as the GEBVs for ten minerals.

The original application of the algorithm is used to test the co-expression between genes by correlation analysis between expression values^15^. First, this algorithm calculates the strength of the linear relationship between every two elements in a trio, independent of the third one, using a calculation of the partial correlation for each trio of elements based on the expression values in a specific set of samples. Then, the algorithm sets an information theory threshold for each significant association, by calculating the average ratio of partial to direct correlations for each trio of elements. In our application, we included the GEBVs for each one of the ten minerals evaluated in each sample in the elements tested by the algorithm along with the gene and miRNA expression values (herein called PCIT general). Using this approach, we estimated the correlations among all the elements. Among the significant correlations, we selected only the genes and miRNAs with expression values correlated to the GEBV of at least one mineral. Due to the low number of miRNAs identified compared to the high number of genes, we did one more PCIT analysis only with the expression values for miRNAs and the GEBVs (herein called PCIT miRNA). The results of these two PCITs analyses were combined. At the end, we had a list of elements (genes and miRNAs) with expression values correlated to each mineral GEBV.

### RIF (regulatory impact factor)

A new application of the RIF algorithm^16^ was adopted to predict the regulatory impact of the genes and miRNAs with expression values associated to a given mineral’s amount GEBVs. The algorithm was originally developed to determine the regulatory impact of TFs over selected genes (targets) related to a given trait through their expression values analysis between contrasting groups for the same trait^16^. To this end, the impact factor is calculated two scores (RIF 1 and RIF 2). RIF 1 gives high scores to TFs that are most differentially co-expressed, highly abundant, and with more expression difference between the groups. RIF 2 gives a high score to TFs whose expression can predict better the abundance of DEGs^16^. In our approach we used the genes and miRNAs with expression values correlated to each mineral, from the previous PCIT analyses, as elements to be tested as regulators and the mineral GEBV as the target, in the place of the TFs in the original application. We used an analogy to the concepts used originally to calculate RIF1 and RIF2, *i.e*., RIF1 gave high score to mRNAs and miRNAs that were most differentially co-expressed, highly abundant, and with more expression difference between the groups of minerals. RIF 2 gave a high score to mRNAs and miRNAs whose expression can predict better the GEBV for mineral amount.

We carried out 10 different analyses with the RIF algorithm^16^, being one for each mineral. As input, we used the GEBVs for the 30 contrasting samples for each mineral as targets (15 representing samples with high mineral mass fraction and 15 with low mineral mass fraction) and the expression values for the genes and miRNAs correlated to the same mineral as elements to be tested. To select these contrasting groups we expanded the sample selection based on GEBVs previously made^9,10^. Genes and miRNAs with RIF I or II scores higher than |1.96| were considered as significant, as authors suggest^16^.

### RIF for all minerals together

To identify genes and miRNAs with significant impact factor in all minerals’ mass fraction together, we used the new application for the RIF algorithm^16^ by applying the GEBV from 30 contrasting samples forming two groups regarding the amount of the ten minerals as targets and the expression values for the genes and miRNAs correlated to at least one mineral as elements to be tested.

To select contrasting samples for all the minerals together, we ranked our samples based on a score. To calculate this score for each sample, we performed a principal component analysis (PCA) using the GEBVs for the ten minerals in the 113 samples. From the PCA results, the score of each sample was calculated based on the following formula:

$${A}_{i}\,=\,\mathop{\sum }\limits_{j=i}^{10}kContri{b}_{ijk}\,\times \,{Z}_{ijk}\,\times  \% {V}_{PCj}$$

Where: *A*_*i*_ = *score* for the animal *i*, $\mathop{\sum }\limits_{j=i}^{10}k\,=$ sum of all minerals k, in all the PCs *j* and in all the animals *i*, $Contri{b}_{ijk}\,=$ contribution of the animal *i* in the PC *j* for the mineral *k*, ${Z}_{ijk}\,=$ standardized value (standard deviation one and mean zero) of the GEBV for the mineral *k* for the animal *i* in the PC *j* and $\% {V}_{PCj}\,=$ eigenvalue of the PC *j*.

We performed a functional annotation analysis using DAVID 6.8 software^77^ with the genes presenting significant RIFs for this score.

### Genes and miRNAs correlated to minerals

Significant correlations obtained from PCIT^15^ analysis between genes or miRNAs expressions and minerals were used to build a co-expression network with the Cytoscape software^78^. We overlapped the gene list from our network with the genes previously reported by our research group to be differentially expressed for at least one mineral, based on the same population evaluated here^9,10^, TFs^17^, genes affected by cis or trans eQTLs^18^ and with significant RIF in the present work. These features were used as attributes in the network. Regarding the differentially expressed genes (DEGs) described for Fe^10^, we called the genes more expressed in the high Fe content group as upregulated whereas genes more expressed in the low Fe content group were called as downregulated, to match the nomination of the other minerals’ DEGs^9^. Functional annotation analyses were made using DAVID 6.8 software, considering significant the pathways presenting a p-value corrected for multiple tests by Benjamini of less than 0.05^77^.

### Integration with DEGs

To estimate the relationship among the genes or miRNAs whose expression values were correlated with minerals and the DEGs previously detected between contrasting groups of mineral concentration^9^, we made ten separately PCIT^15^ analysis. In these analyses, the PCIT algorithm was used as proposed initially^15^ to test the correlations among the genes and miRNAs with expression values correlated to each mineral, and the DEGs previously detected for the same mineral^9,10^.

The significant correlations identified in each analysis were used to obtain co-expression networks with the Cytoscape software^78^. The NetworkAnalyzer tool of the Cytoscape software^78^ was used to obtain the connectivity degree of each gene and miRNA in the networks. This value was used to identify the hub genes/hub miRNAs, that were obtained from the average of the connectivity degree of the network summed with the double of the standard deviation.

We considered only the significant correlations containing at least a hub or significant RIF gene/miRNA for a given mineral. The genes present in these correlations were used to perform a functional annotation analysis with the STRING v.1.2.2 software^79^. From these analyses, we selected the genes being part of enriched pathways, considering KEGG^80^ and Reactome^19^ databases with *Bos taurus* reference genome.

### Putative regulators of the genes being part of enriched pathways

To identify the elements putatively regulating the genes being part of over-represented pathways in the study, we did another round of PCIT^15^ analyses, separately for each mineral. In this case, from the last PCIT analysis by mineral, we selected as inputs the genes being part of enriched pathways, also considering the previously enriched pathways from differentially expressed genes related to mineral amount^9,10^, the hub elements, TFs^17^, miRNAs and the ones with significant RIFs, with their respective attributes. The PCIT^15^ results were used to obtain co-expression networks with Cytoscape^78^ software.

### miRNA-gene targeting confirmation

We used TargetScan software^81^ to predict the target genes for the miRNAs with expression values significantly correlated to a mineral and compared these putative targets with the genes with expression values correlated to them in our networks.

## Supplementary information

Supplementary information.
