# Supplementary material for: Genetic regulators of mineral amount in Nelore cattle muscle predicted by a new co-expression and regulatory impact factor approach
Source: Sci Rep. 2020 May 21;10:8436. doi: 10.1038/s41598-020-65454-7 (PMC7242321; doi:10.1038/s41598-020-65454-7)
Supplement: Supplementary file 1 — Supplementary information. [file 41598_2020_65454_MOESM1_ESM.pdf]

Genetic regulators of mineral amount in Nelore cattle muscle predicted by a new co-expression and regulatory impact factor approach

Juliana Afonso[1], Marina Rufino Salinas Fortes[2], Antonio Reverter[3], Wellison Jarles da Silva Diniz[1], Aline Silva Mello Cesar[4], Andressa Oliveira de Lima[1], Juliana Petrini[5], Marcela M. de Souza[6], Luiz Lehmann Coutinho[7], Gerson Barreto Mourão[4], Adhemar Zerlotini[8], Caio Fernando Gromboni[9], Ana Rita Araújo Nogueira[10], Luciana Correia de Almeida Regitano[*10]

[1]Department of Evolutionary Genetics and Molecular Biology, Federal University of São Carlos, São Carlos, Brazil, [2]School of Chemistry and Molecular Biosciences, Faculty of Sciences, The University of Queensland, Brisbane, Australia, [3]Agriculture and Food, Commonwealth Scientific and Industrial Research Organisation, Brisbane, Australia, [4]Department of Agroindustry, Food and Nutrition, University of São Paulo/ESALQ, Piracicaba, Brazil, [5]Department of Statistics, Institute of Exact Sciences, Federal University of Alfenas, Alfenas, Brazil, [6]Department of Animal Science, Iowa State University, Ames, IA, USA, [7]Department of Animal Science, University of São Paulo/ESALQ, Piracicaba, Brazil, [8]Bioinformatic Multi-user Laboratory, Embrapa Informática Agropecuária, Campinas, São Paulo, Brazil.[9] Bahia Federal Institute of Education, Science and Technology, Ilhéus, Brazil,[10]Embrapa Pecuária Sudeste, São Carlos, Brazil, [*]Corresponding author.

**Correspondence to: luciana.regitano@embrapa.br*

Supplementary Information

**Legend**	
**corr**	Genes correlated to a mineral amount.
**eQTL_cis**	Genes being affected by cis eQTL.
**eQTL_trans**	Genes being affected by trans eQTL.
**miRNA**	micro RNAs.
**RIF_ mineral name or score**	Genes or miRNA presenting a significant regulatory impact over specific mineral amount.
**TF**	Transcription factor
**DEG_ mineral name**	Gene differentially expressed regarding specific mineral amount.
**down**	genes differentially expressed more expressed in the low mineral amount group.
**up**	genes differentially expressed more expressed in the high mineral amount group.
**hub**	genes and miRNAs identified as hub elements in the co-expression networks.
**pathways**	genes partaking over-represented pathways.

Supplementary Table S1. Genes and miRNAs with significant RIF for each mineral or score analysis. The bold values are the significant, higher than |1.96|.

	**Score**		**Ca**		**Cu**		**K**		**Mg**		**Na**		**P**		**S**		**Se**		**Zn**		**Fe**	
**Elements**	**RIF1**	**RIF2**	**RIF1**	**RIF2**	**RIF1**	**RIF2**	**RIF1**	**RIF2**	**RIF1**	**RIF2**	**RIF1**	**RIF2**	**RIF1**	**RIF2**	**RIF1**	**RIF2**	**RIF1**	**RIF2**	**RIF1**	**RIF2**	**RIF1**	**RIF2**
ALAD	-1.92	**-2.97**																				
bta.let.7i									**3.40**	-0.10												
bta.miR.125a											-0.36	**2.09**										
bta.miR.181a	-1.14	**-2.81**																				
bta.miR.199b																			**2.17**	1.41		
bta.miR.25	0.12	**-3.09**																			**-2.59**	-1.56
bta.miR.369.3p			**3.00**	-0.76											**2.92**	1.95						
bta.miR.378c																					**-2.82**	-1.14
bta.miR.411c.5p																			**4.73**	1.73		
bta.miR.500															0.42	**2.05**						
bta.miR.92b							-0.64	**2.33**														
CAMKK1											-0.45	**2.28**										
CD86							**2.46**	-0.29	**2.22**	-0.64												
CDKN3											**2.29**	**3.84**										
CENPE											**3.17**	0.05										
CITED4	-0.41	**-2.59**																				
CLBA1	**-1.98**	-1.94																				
CTH									1.02	**4.14**												
DPP4	**-4.39**	**-3.40**																				
DTWD1																	**-3.76**	1.55				
ECHDC2	**-4.54**	-1.94																				
FUT8															1.08	**3.12**						
HARS																	**-2.60**	0.25				
LOC101905675	**-2.33**	**-4.23**																				
LOC101907603	**-2.03**	0.13																				
LOC104968807	-1.72	**-2.87**																				
LOC112441773	0.22	**-2.45**																				
LOC112442312																	**-2.72**	1.12				
LOC112446381	**-4.24**	**-2.76**																				
LOC509513	**-4.24**	**-2.00**																				

LOC518768					**2.40**	1.43																
LOC530929					**2.94**	1.45																
LOC784127					**2.06**	-0.13																
LPAR4			**3.41**	**-4.07**																		
MBTPS2																			0.47	**2.10**		
MCM4	**-2.23**	0.19																				
MCPH1	0.16	**-2.05**																				
METTL21E															**2.34**	-0.23						
MMP16							0.79	**-2.18**														
MYLK3	**-2.05**	**-2.08**																				
NOX1																			-0.72	**2.24**		
NUDT18																			0.59	**2.78**		
PDK3																	-0.02	**-2.02**				
PIGS	**-3.57**	-1.38																				
PLPPR5															**1.97**	0.51						
POLR3E	-0.90	**-2.44**																				
PPDPFL	**-3.85**	**-2.46**																				
PRKG2	**-5.58**	**-3.26**																				
PRRG3															**3.23**	1.19						
RAB44															**1.99**	0.97						
RASL11A					0.45	**2.67**																
RNF34							**3.54**	**2.06**														
SLC22A4	0.72	**-3.09**																				
TEF																	0.12	**-2.16**				
TENM4	-1.57	**-2.51**																				
TMEM238	**-2.51**	-1.60																				
TNR																			0.44	**2.34**		
TTC21A																	-0.66	**-2.07**				
VDR									**2.75**	-0.68	**2.02**	-0.44										
VMAC											**2.63**	0.44										
WDPCP											1.12	**2.07**	**3.07**	**3.12**								
ZCCHC7	**-2.58**	**-2.56**																				
ZDBF2																	-0.36	**-2.56**				

Supplementary Table S2. Correlations and attributes constituting Figure 1 (A, B and C). The different attributes of the origin or the target regarding each correlation are represented separated by an underline sign.

GEBV	Gene	Correlation value	Correlation type	Gene attributes
Calcium	*ELL*	0.28748	POS	eQTL_trans
Calcium	*FAM89A*	-0.2989	NEG	eQTL_trans
Calcium	*FDXACB1*	-0.25702	NEG	eQTL_trans
Calcium	*MIR29E*	0.25726	POS	miRNA
Calcium	*LPAR4*	-0.28643	NEG	RIF_Ca
Calcium	*LOC101907603*	0.24419	POS	RIF_Score
Calcium	*ZNF131*	0.2465	POS	TF
Calcium	*VDR*	-0.28435	NEG	TF_RIF_Mg_and_Na
Calcium	*AAR2*	-0.30422	NEG	corr
Calcium	*BAAT*	0.26738	POS	corr
Calcium	*BMF*	-0.28173	NEG	corr
Calcium	*CDK8*	0.31904	POS	corr
Calcium	*COG4*	-0.25888	NEG	corr
Calcium	*LOC101908204*	0.22265	POS	corr
Calcium	*LOC112442262*	-0.30615	NEG	corr
Calcium	*LOC112449059*	0.26905	POS	corr
Calcium	*LOC510362*	-0.27261	NEG	corr
Calcium	*OTOR*	-0.22439	NEG	corr
Calcium	*RNF165*	-0.25848	NEG	corr
Calcium	*TBL2*	-0.27247	NEG	corr
Calcium	*THSD7B*	-0.29941	NEG	corr
Calcium	*VNN2*	-0.30558	NEG	corr
Copper	*MEST*	-0.33985	NEG	DEG_Ca_Cu_Mg_K_P
Copper	*TNFRSF11B*	-0.31267	NEG	eQTL_trans
Copper	*LOC518768*	0.28322	POS	RIF_Cu
Copper	*LOC530929*	-0.34437	NEG	RIF_Cu
Copper	*LOC784127*	-0.28084	NEG	RIF_Cu
Copper	*RASL11A*	0.30493	POS	RIF_Cu
Copper	*BHLHE22*	-0.27301	NEG	TF
Copper	*ALG11*	-0.29913	NEG	corr
Copper	*CADM4*	0.26371	POS	corr
Copper	*CAMK2N2*	-0.31188	NEG	corr
Copper	*CENPN*	0.2902	POS	corr
Copper	*CERS4*	-0.24666	NEG	corr
Copper	*CLDN19*	0.22968	POS	corr
Copper	*DHX40*	-0.24675	NEG	corr
Copper	*DIAPH3*	-0.29391	NEG	corr
Copper	*FAM229A*	0.29583	POS	corr
Copper	*GPRC5A*	0.28708	POS	corr
Copper	*KIAA0408*	-0.24594	NEG	corr
Copper	*KLHL7*	-0.26588	NEG	corr
Copper	*LOC100847269*	0.24908	POS	corr
Copper	*LOC101907322*	-0.27148	NEG	corr
Copper	*LOC511409*	0.29005	POS	corr
Copper	*LOC514257*	0.22999	POS	corr
Copper	*LRRC56*	0.23991	POS	corr
Copper	*MASTL*	-0.32249	NEG	corr
Copper	*NDC80*	-0.31721	NEG	corr
Copper	*NUCB2*	-0.28926	NEG	corr

Copper	*PURG*	-0.32558	NEG	corr
Copper	*RASAL1*	0.30467	POS	corr
Copper	*RASGEF1C*	0.22686	POS	corr
Copper	*RGS7*	-0.23266	NEG	corr
Copper	*SGCE*	-0.30115	NEG	corr
Copper	*SKIDA1*	-0.23513	NEG	corr
Copper	*TINF2*	0.28356	POS	corr
Copper	*VEGFD*	-0.26386	NEG	corr
Iron	*SLC22A4*	-0.2616	NEG	DEG_Fe_RIF_Score
Iron	*HPCAL4*	-0.2958	NEG	DEG_S_and_Fe
Iron	*MYLK3*	-0.23175	NEG	DEG_S_Fe_RIF_Score
Iron	*PLCB2*	-0.29366	NEG	eQTL_trans
Iron	*bta-miR-25*	-0.29391	NEG	RIF_Fe_and_Score_miRNA
Iron	*ALAD*	-0.29944	NEG	RIF_score
Iron	*CITED4*	-0.3213	NEG	RIF_Score
Iron	*CLBA1*	0.29126	POS	RIF_Score
Iron	*DPP4*	-0.30184	NEG	RIF_Score
Iron	*LOC101905675*	0.25872	POS	RIF_Score
Iron	*LOC104968807*	0.28433	POS	RIF_Score
Iron	*LOC112441773*	0.30253	POS	RIF_Score
Iron	*LOC112446381*	0.2805	POS	RIF_Score
Iron	*PPDPFL*	-0.27818	NEG	RIF_Score
Iron	*PRKG2*	0.29824	POS	RIF_Score
Iron	*TENM4*	0.28402	POS	RIF_Score
Iron	*TMEM238*	0.26744	POS	RIF_Score
Iron	*ZCCHC7*	0.26352	POS	RIF_Score
Iron	*MCPH1*	0.37197	POS	RIF_Score_eQTL_trans
Iron	*C8H9orf72*	-0.28749	NEG	corr
Iron	*EPOR*	0.25625	POS	corr
Iron	*FABP7*	0.27134	POS	corr
Iron	*LOC101906717*	0.30183	POS	corr
Iron	*LOC107132942*	0.31852	POS	corr
Iron	*LRRC32*	0.30712	POS	corr
Iron	*OLFM2*	0.25687	POS	corr
Iron	*SH2D2A*	-0.28015	NEG	corr
Iron	*TSPEAR*	-0.2662	NEG	corr
Magnesium	*COL21A1*	-0.24532	NEG	DEG_Ca
Magnesium	*PLXDC1*	-0.24977	NEG	DEG_Ca
Magnesium	*COL11A2*	-0.28204	NEG	DEG_Ca_Cu_Mg_K_Na_P_eQTL_trans
Magnesium	*MMP16*	-0.28169	NEG	DEG_Ca_Mg_RIF_K
Magnesium	*ADAP1*	0.22606	POS	eQTL_trans
Magnesium	*LIMD2*	-0.27085	NEG	eQTL_trans
Magnesium	*CTH*	-0.26794	NEG	RIF_Mg
Magnesium	*CD86*	-0.26727	NEG	RIF_Mg_and_K
Magnesium	*WDPCP*	-0.2958	NEG	RIF_Na_and_P_eQTL_trans
Magnesium	*FUT8*	-0.26676	NEG	RIF_S_eQTL_trans
Magnesium	*LOC509513*	-0.29215	NEG	RIF_Score
Magnesium	*PIGS*	-0.24335	NEG	RIF_Score
Magnesium	*ZIC3*	0.27598	POS	TF
Magnesium	*VDR*	-0.29003	NEG	TF_RIF_Mg_and_Na
Magnesium	*ADA2*	-0.26432	NEG	corr
Magnesium	*ARHGAP6*	-0.27247	NEG	corr
Magnesium	*BAAT*	0.28394	POS	corr

Magnesium	*BCL2L15*	-0.26146	NEG	corr
Magnesium	*CARD14*	-0.32134	NEG	corr
Magnesium	*CD5*	-0.27967	NEG	corr
Magnesium	*CYBC1*	-0.28235	NEG	corr
Magnesium	*DCX*	-0.28272	NEG	corr
Magnesium	*DOC2A*	-0.28694	NEG	corr
Magnesium	*FCGR2A*	-0.2343	NEG	corr
Magnesium	*FYB1*	-0.24905	NEG	corr
Magnesium	*GIMAP5*	-0.22633	NEG	corr
Magnesium	*IFIT3*	-0.23621	NEG	corr
Magnesium	*LOC100847708*	-0.25417	NEG	corr
Magnesium	*LOC112442227*	-0.26952	NEG	corr
Magnesium	*LOC112443416*	-0.29748	NEG	corr
Magnesium	*LOC617875*	-0.27305	NEG	corr
Magnesium	*LOC618071*	-0.23885	NEG	corr
Magnesium	*PARVG*	-0.23735	NEG	corr
Magnesium	*RIC8A*	-0.24584	NEG	corr
Magnesium	*RNF165*	-0.28931	NEG	corr
Magnesium	*TMEM74*	0.30371	POS	corr
Magnesium	*ZDHHC24*	-0.23565	NEG	corr
Phosphorus	*COL21A1*	-0.26028	NEG	DEG_Ca
Phosphorus	*MMP16*	-0.27273	NEG	DEG_Ca_Mg_RIF_K
Phosphorus	*ELL*	0.2866	POS	eQTL_trans
Phosphorus	*WDPCP*	-0.27636	NEG	RIF_Na_and_P_eQTL_trans
Phosphorus	*FUT8*	-0.25535	NEG	RIF_S_eQTL_trans
Phosphorus	*SALL4*	0.22611	POS	TF
Phosphorus	*VDR*	-0.29573	NEG	TF_RIF_Mg_and_Na
Phosphorus	*BAAT*	0.29176	POS	corr
Phosphorus	*BOLA.DOA*	-0.24521	NEG	corr
Phosphorus	*CARD14*	-0.31459	NEG	corr
Phosphorus	*CD5*	-0.26699	NEG	corr
Phosphorus	*DCX*	-0.29777	NEG	corr
Phosphorus	*DNASE1L3*	-0.25444	NEG	corr
Phosphorus	*LOC100847708*	-0.23176	NEG	corr
Phosphorus	*LOC112442227*	-0.30324	NEG	corr
Phosphorus	*LOC112443416*	-0.29702	NEG	corr
Phosphorus	*LOC785503*	-0.23372	NEG	corr
Phosphorus	*RNF165*	-0.29805	NEG	corr
Phosphorus	*TMEM74*	0.26101	POS	corr
Potassium	*COL21A1*	-0.26516	NEG	DEG_Ca
Potassium	*COL11A2*	-0.25476	NEG	DEG_Ca_Cu_Mg_K_Na_P_eQTL_trans
Potassium	*ARSA*	-0.26049	NEG	DEG_Ca_eQTL_trans
Potassium	*ANGPTL2*	-0.2645	NEG	DEG_Ca_Mg_K
Potassium	*MMP16*	-0.27796	NEG	DEG_Ca_Mg_RIF_K
Potassium	*INSIG2*	0.24382	POS	eQTL_trans
Potassium	*LIMD2*	-0.29737	NEG	eQTL_trans
Potassium	*RNF34*	0.25231	POS	RIF_K
Potassium	*CD86*	-0.2624	NEG	RIF_Mg_and_K
Potassium	*WDPCP*	-0.2912	NEG	RIF_Na_and_P_eQTL_trans
Potassium	*FUT8*	-0.24579	NEG	RIF_S_eQTL_trans
Potassium	*MCM4*	-0.23484	NEG	RIF_Score_eQTL_trans
Potassium	*ZIC3*	0.3187	POS	TF
Potassium	*VDR*	-0.31361	NEG	TF_RIF_Mg_and_Na

Potassium	*ADA2*	-0.27013	NEG	corr
Potassium	*ARAP1*	-0.24212	NEG	corr
Potassium	*ARHGAP30*	-0.2767	NEG	corr
Potassium	*BCL2L15*	-0.25165	NEG	corr
Potassium	*BOLA.DOA*	-0.24617	NEG	corr
Potassium	*CARD14*	-0.3083	NEG	corr
Potassium	*CD5*	-0.25609	NEG	corr
Potassium	*CYBC1*	-0.27738	NEG	corr
Potassium	*DCX*	-0.30709	NEG	corr
Potassium	*DNASE1L3*	-0.26622	NEG	corr
Potassium	*DOC2A*	-0.30164	NEG	corr
Potassium	*KIAA2012*	0.22718	POS	corr
Potassium	*LOC112442227*	-0.29797	NEG	corr
Potassium	*LOC112443416*	-0.30263	NEG	corr
Potassium	*LOC613985*	-0.28296	NEG	corr
Potassium	*LOC617875*	-0.2534	NEG	corr
Potassium	*RNF165*	-0.29279	NEG	corr
Potassium	*TMEM74*	0.26858	POS	corr
Potassium	*TRIP13*	-0.25022	NEG	corr
Selenium	*CISH*	-0.28436	NEG	DEG_S_and_Zn
Selenium	*ECHDC2*	0.29528	POS	DEG_Se_RIF_Score
Selenium	*PLCE1*	-0.27924	NEG	eQTL_cis
Selenium	*GCNT4*	-0.27806	NEG	eQTL_trans
Selenium	*TMED6*	-0.26239	NEG	eQTL_trans
Selenium	*bta-miR-425-5p*	0.29692	POS	miRNA
Selenium	*POLR3E*	-0.28805	NEG	RIF_Score
Selenium	*LOC112442312*	-0.29381	NEG	RIF_Se
Selenium	*PDK3*	0.25654	POS	RIF_Se
Selenium	*TTC21A*	-0.31632	NEG	RIF_Se
Selenium	*ZDBF2*	-0.25051	NEG	RIF_Se
Selenium	*HARS*	0.27995	POS	RIF_Se
Selenium	*DTWD1*	-0.29469	NEG	RIF_Se_eQTL_trans
Selenium	*NOX1*	-0.31542	NEG	RIF_Zn
Selenium	*bta-miR-411c-5p*	0.22427	POS	RIF_Zn_miRNA
Selenium	*RFX3*	-0.26994	NEG	TF
Selenium	*TEF*	0.31366	POS	TF_RIF_Se
Selenium	*B3GNT5*	-0.304	NEG	corr
Selenium	*CEP164*	-0.28799	NEG	corr
Selenium	*COL28A1*	0.25388	POS	corr
Selenium	*EML5*	-0.29815	NEG	corr
Selenium	*KANTR*	-0.28199	NEG	corr
Selenium	*LGR6*	-0.26007	NEG	corr
Selenium	*LOC101907941*	-0.347	NEG	corr
Selenium	*LOC104973799*	-0.26621	NEG	corr
Selenium	*LOC781977*	0.27438	POS	corr
Selenium	*LSM14A*	-0.2782	NEG	corr
Selenium	*PGAP2*	0.29148	POS	corr
Selenium	*PRKN*	0.22738	POS	corr
Selenium	*SNX25*	-0.27771	NEG	corr
Selenium	*SRRM4*	-0.24893	NEG	corr
Selenium	*TBCC*	0.29396	POS	corr
Selenium	*ZDHHC17*	-0.26022	NEG	corr
Selenium	*ZNF879*	-0.27737	NEG	corr

Sodium	*COL21A1*	-0.27608	NEG	DEG_Ca
Sodium	*COL11A2*	-0.27454	NEG	DEG_Ca_Cu_Mg_K_Na_P_eQTL_trans
Sodium	*MEST*	-0.27057	NEG	DEG_Ca_Cu_Mg_K_P
Sodium	*MMP16*	-0.29696	NEG	DEG_Ca_Mg_RIF_K
Sodium	*CRABP2*	-0.22034	NEG	DEG_Mg_K_Na_P_eQTL_trans
Sodium	*MAOB*	-0.26284	NEG	DEG_Mg_K_Na_S_eQTL_trans
Sodium	*CCR2*	-0.26762	NEG	eQTL_trans
Sodium	*CTNS*	-0.23875	NEG	eQTL_trans
Sodium	*GAL3ST4*	-0.25026	NEG	eQTL_trans
Sodium	*LIMD2*	-0.25136	NEG	eQTL_trans
Sodium	*LOXL3*	-0.29781	NEG	eQTL_trans
Sodium	*MARK3*	0.26859	POS	eQTL_trans
Sodium	*TTC39A*	-0.27635	NEG	eQTL_trans
Sodium	*bta-miR-130b*	-0.23139	NEG	miRNA
Sodium	*bta-miR-22-5p*	0.27415	POS	miRNA
Sodium	*bta-miR-92b*	-0.27755	NEG	RIF_K_miRNA
Sodium	*CAMKK1*	-0.26731	NEG	RIF_Na
Sodium	*CDKN3*	-0.28047	NEG	RIF_Na
Sodium	*CENPE*	-0.22806	NEG	RIF_Na
Sodium	*WDPCP*	-0.27218	NEG	RIF_Na_and_P_eQTL_trans
Sodium	*VMAC*	-0.23703	NEG	RIF_Na_eQTL_trans
Sodium	*FUT8*	-0.31291	NEG	RIF_S_eQTL_trans
Sodium	*ZIC3*	0.27854	POS	TF
Sodium	*VDR*	-0.24407	NEG	TF_RIF_Mg_and_Na
Sodium	*ABCB10*	0.29013	POS	corr
Sodium	*ADA2*	-0.26836	NEG	corr
Sodium	*ARAP1*	-0.26727	NEG	corr
Sodium	*BCL2L15*	-0.24606	NEG	corr
Sodium	*BOLA.DOA*	-0.31489	NEG	corr
Sodium	*CD5*	-0.30633	NEG	corr
Sodium	*CENPK*	-0.2325	NEG	corr
Sodium	*DCX*	-0.26866	NEG	corr
Sodium	*DNASE1L3*	-0.30842	NEG	corr
Sodium	*FGD2*	-0.2388	NEG	corr
Sodium	*IKBKE*	-0.25977	NEG	corr
Sodium	*KIAA2012*	0.24586	POS	corr
Sodium	*LOC112443416*	-0.30465	NEG	corr
Sodium	*LSM14B*	0.26136	POS	corr
Sodium	*MGME1*	-0.24265	NEG	corr
Sodium	*RCE1*	-0.28632	NEG	corr
Sodium	*RNF165*	-0.27684	NEG	corr
Sodium	*SLA*	-0.28033	NEG	corr
Sodium	*THSD7B*	-0.27147	NEG	corr
Sodium	*TMEM74*	0.29169	POS	corr
Sodium	*XCR1*	-0.2605	NEG	corr
Sulfur	*C1QTNF3*	-0.28331	NEG	DEG_Ca_and_S
Sulfur	*ARSA*	-0.30672	NEG	DEG_Ca_eQTL_trans
Sulfur	*MMP16*	-0.28762	NEG	DEG_Ca_Mg_RIF_K
Sulfur	*LOC515150*	-0.27486	NEG	DEG_Na_and_P
Sulfur	*CCR2*	-0.28146	NEG	eQTL_trans
Sulfur	*LIMD2*	-0.28315	NEG	eQTL_trans
Sulfur	*NXPE4*	-0.23989	NEG	eQTL_trans
Sulfur	*PLCB2*	-0.27829	NEG	eQTL_trans

Sulfur	*TIAM1*	-0.26651	NEG	eQTL_trans
Sulfur	*bta-miR-365-3p*	0.30654	POS	miRNA
Sulfur	*LPAR4*	-0.24193	NEG	RIF_Ca
Sulfur	*CD86*	-0.26317	NEG	RIF_Mg_and_K
Sulfur	*WDPCP*	-0.299	NEG	RIF_Na_and_P_eQTL_trans
Sulfur	*METTL21E*	0.23864	POS	RIF_S
Sulfur	*PLPPR5*	-0.25789	NEG	RIF_S
Sulfur	*PRRG3*	-0.33992	NEG	RIF_S
Sulfur	*RAB44*	-0.24272	NEG	RIF_S
Sulfur	*FUT8*	-0.24658	NEG	RIF_S_eQTL_trans
Sulfur	*PIGS*	-0.27016	NEG	RIF_Score
Sulfur	*BCL11B*	-0.24787	NEG	TF
Sulfur	*IKZF3*	-0.31237	NEG	TF
Sulfur	*VDR*	-0.32126	NEG	TF_RIF_Mg_and_Na
Sulfur	*ADA2*	-0.29552	NEG	corr
Sulfur	*AMACR*	-0.24203	NEG	corr
Sulfur	*ARAP1*	-0.29412	NEG	corr
Sulfur	*ARHGAP30*	-0.29766	NEG	corr
Sulfur	*BOLA.DOA*	-0.32479	NEG	corr
Sulfur	*BTK*	-0.31311	NEG	corr
Sulfur	*C27H4orf47*	0.30264	POS	corr
Sulfur	*CD5*	-0.30512	NEG	corr
Sulfur	*CD53*	-0.28108	NEG	corr
Sulfur	*DAGLB*	0.25524	POS	corr
Sulfur	*DCX*	-0.29458	NEG	corr
Sulfur	*FCGR2A*	-0.25147	NEG	corr
Sulfur	*FLT3*	-0.28344	NEG	corr
Sulfur	*FYN*	-0.28861	NEG	corr
Sulfur	*GIMAP5*	-0.28994	NEG	corr
Sulfur	*HEBP2*	-0.2519	NEG	corr
Sulfur	*JAML*	-0.25595	NEG	corr
Sulfur	*LOC101907383*	0.24301	POS	corr
Sulfur	*LOC510860*	-0.24964	NEG	corr
Sulfur	*LOC534578*	-0.28335	NEG	corr
Sulfur	*LOC785503*	-0.27903	NEG	corr
Sulfur	*MCCD1*	0.28112	POS	corr
Sulfur	*PAG2*	-0.26218	NEG	corr
Sulfur	*PPT1*	-0.31558	NEG	corr
Sulfur	*RBM24*	0.28995	POS	corr
Sulfur	*RSBN1L*	0.29938	POS	corr
Sulfur	*SIGLEC5*	-0.26835	NEG	corr
Sulfur	*SLA*	-0.2661	NEG	corr
Sulfur	*SRD5A3*	-0.26023	NEG	corr
Sulfur	*TMEM74*	0.31518	POS	corr
Sulfur	*TNFAIP3*	-0.28388	NEG	corr
Sulfur	*WDHD1*	-0.26582	NEG	corr
Sulfur	*XCR1*	-0.28886	NEG	corr
Sulfur	*XRCC6*	-0.26597	NEG	corr
Zinc	*CHPT1*	0.29913	POS	corr
Zinc	*COL11A2*	-0.30055	NEG	DEG_Ca_Cu_Mg_K_Na_P_eQTL_trans
Zinc	*ANGPTL2*	-0.28977	NEG	DEG_Ca_Mg_K
Zinc	*INSIG2*	0.26396	POS	eQTL_trans
Zinc	*MIR133A.2*	0.26658	POS	miRNA

Zinc	*MIR29E*	0.25458	POS	miRNA
Zinc	*MBTPS2*	0.31293	POS	RIF_Zn
Zinc	*NOX1*	0.34177	POS	RIF_Zn
Zinc	*TNR*	-0.24344	NEG	RIF_Zn
Zinc	*NUDT18*	-0.27722	NEG	RIF_Zn_eQTL_trans
Zinc	*ZIC3*	0.34155	POS	TF
Zinc	*AAR2*	-0.28479	NEG	corr
Zinc	*ASF1B*	-0.26922	NEG	corr
Zinc	*BAAT*	0.23357	POS	corr
Zinc	*C7H19orf67*	-0.27159	NEG	corr
Zinc	*CTSD*	-0.26317	NEG	corr
Zinc	*DCX*	-0.29826	NEG	corr
Zinc	*FAIM*	0.33274	POS	corr
Zinc	*GABPB1*	0.27915	POS	corr
Zinc	*GID8*	-0.27925	NEG	corr
Zinc	*GRM4*	-0.28273	NEG	corr
Zinc	*LEMD3*	0.35822	POS	corr
Zinc	*LOC101905734*	-0.30154	NEG	corr
Zinc	*LOC107131496*	0.24035	POS	corr
Zinc	*LOC107132969*	-0.27363	NEG	corr
Zinc	*LOC112443416*	-0.26314	NEG	corr
Zinc	*LOC112446096*	-0.25666	NEG	corr
Zinc	*LOC514189*	0.2311	POS	corr
Zinc	*LOC613985*	-0.321	NEG	corr
Zinc	*LOC617875*	-0.25932	NEG	corr
Zinc	*LTV1*	0.244	POS	corr
Zinc	*NBN*	0.24718	POS	corr
Zinc	*RGMA*	-0.24903	NEG	corr
Zinc	*SAT2*	-0.29135	NEG	corr
Zinc	*TTC9*	-0.29346	NEG	corr
Zinc	*VPS18*	-0.30612	NEG	corr
Zinc	*ZCCHC10*	0.25584	POS	corr
Zinc	*ZNF770*	0.30012	POS	corr
Calcium	Magnesium	0.65159	POS	
Calcium	Phosphorus	0.67441	POS	
Calcium	Potassium	0.65292	POS	
Calcium	Sodium	0.62988	POS	
Calcium	Sulfur	0.65365	POS	
Calcium	Zinc	0.62582	POS	
Copper	Magnesium	0.25852	POS	
Iron	Magnesium	0.26979	POS	
Iron	Phosphorus	0.27908	POS	
Iron	Potassium	0.27488	POS	
Iron	Sulfur	0.3168	POS	
Magnesium	Phosphorus	0.97196	POS	
Magnesium	Potassium	0.97319	POS	
Magnesium	Sodium	0.90216	POS	
Magnesium	Sulfur	0.80976	POS	
Magnesium	Zinc	0.79179	POS	
Phosphorus	Potassium	0.9713	POS	
Phosphorus	Sodium	0.90035	POS	
Phosphorus	Sulfur	0.83216	POS	
Phosphorus	Zinc	0.80431	POS	

Potassium	Sodium	0.89312	POS
Potassium	Sulfur	0.82771	POS
Potassium	Zinc	0.79254	POS
Sodium	Sulfur	0.79933	POS
Sodium	Zinc	0.77209	POS
Sulfur	Zinc	0.67963	POS

Supplementary Table S3. Correlations and attributes of each significant correlation constituting Figure 3

Mg

**Origin**	**Target**	**Correlation value**	**Correlation type**	**Origin attributes**	**Target attributes**
ADA2	bta-miR-22-5p	-0.13427	NEG	corr_hub	corr_miRNA
ADA2	*CD44*	0.4937	POS	corr_hub	down_pathways
ADA2	*CD86*	0.5016	POS	corr_hub	corr_RIF_hub
ADA2	*COL12A1*	0.34418	POS	corr_hub	down_pathways
ADA2	*MMP16*	0.46043	POS	corr_hub	corr_down_hub_pathways
ADA2	*PIGS*	0.37921	POS	corr_hub	corr_hub
ADA2	*PLXDC1*	0.4142	POS	corr_hub	corr_hub
ADA2	*PRRX2*	0.38604	POS	corr_hub	down_TF
ADA2	*TNC*	0.37287	POS	corr_hub	down_trans_pathways
ADA2	*VDR*	0.39319	POS	corr_hub	corr_TF_RIF
ADAM12	bta-let-7i	0.33608	POS	down_pathways	corr_RIF_miRNA
ADAM12	bta-miR-22-5p	-0.14484	NEG	down_pathways	corr_miRNA
ADAM12	*CD44*	0.40463	POS	down_pathways	down_pathways
ADAM12	*COL11A1*	0.65195	POS	down_pathways	down_pathways
ADAM12	*COL11A2*	0.34259	POS	down_pathways	corr_down_trans_pathways
ADAM12	*COL12A1*	0.62039	POS	down_pathways	down_pathways
ADAM12	*COL21A1*	0.31051	POS	down_pathways	corr_pathways
ADAM12	*COL22A1*	0.62413	POS	down_pathways	down_trans_pathways
ADAM12	*COMP*	0.59696	POS	down_pathways	down_trans_pathways
ADAM12	*ITGA10*	0.58824	POS	down_pathways	down_pathways
ADAM12	*MMP16*	0.48484	POS	down_pathways	corr_down_hub_pathways
ADAM12	*THBS4*	0.62637	POS	down_pathways	down_trans_pathways
ADAM12	*TNC*	0.48486	POS	down_pathways	down_trans_pathways
bta-let-7i	bta-miR-130b	0.18724	POS	corr_RIF_miRNA	corr_miRNA
bta-let-7i	bta-miR-22-5p	-0.13843	NEG	corr_RIF_miRNA	corr_miRNA
bta-let-7i	bta-miR-365-3p	-0.47252	NEG	corr_RIF_miRNA	corr_miRNA
bta-let-7i	bta-miR-365-5p	-0.21895	NEG	corr_RIF_miRNA	corr_miRNA
bta-let-7i	*CD44*	0.25914	POS	corr_RIF_miRNA	down_pathways
bta-let-7i	*COL11A1*	0.31454	POS	corr_RIF_miRNA	down_pathways
bta-let-7i	*COL12A1*	0.34928	POS	corr_RIF_miRNA	down_pathways
bta-let-7i	*COL22A1*	0.26041	POS	corr_RIF_miRNA	down_trans_pathways
bta-let-7i	*COMP*	0.30582	POS	corr_RIF_miRNA	down_trans_pathways
bta-let-7i	*ITGA10*	0.27727	POS	corr_RIF_miRNA	down_pathways
bta-let-7i	*MMP16*	0.29203	POS	corr_RIF_miRNA	corr_down_hub_pathways
bta-let-7i	*PRRX2*	0.31275	POS	corr_RIF_miRNA	down_TF
bta-let-7i	*THBS4*	0.33757	POS	corr_RIF_miRNA	down_trans_pathways
bta-miR-130b	bta-miR-365-5p	-0.23376	NEG	corr_miRNA	corr_miRNA
bta-miR-130b	bta-miR-92b	0.18846	POS	corr_miRNA	corr_miRNA
bta-miR-130b	*CTH*	0.19669	POS	corr_miRNA	corr_RIF
bta-miR-1343-3p	bta-miR-365-5p	0.24281	POS	corr_miRNA	corr_miRNA
bta-miR-1343-3p	*CTH*	-0.2126	NEG	corr_miRNA	corr_RIF
bta-miR-1343-3p	*PIGS*	-0.31264	NEG	corr_miRNA	corr_hub
bta-miR-1343-3p	*ZIC3*	0.22694	POS	corr_miRNA	corr_TF
bta-miR-142-5p	bta-miR-22-5p	-0.23835	NEG	corr_miRNA	corr_miRNA
bta-miR-142-5p	bta-miR-365-3p	-0.28377	NEG	corr_miRNA	corr_miRNA
bta-miR-142-5p	*COL11A2*	0.18951	POS	corr_miRNA	corr_down_trans_pathways
bta-miR-142-5p	*MMP16*	0.1673	POS	corr_miRNA	corr_down_hub_pathways

bta-miR-142-5p	*PRRX2*	0.22458	POS	corr_miRNA	down_TF
bta-miR-22-5p	*ITGA10*	-0.11961	NEG	corr_miRNA	down_pathways
bta-miR-365-3p	*CTH*	-0.18532	NEG	corr_miRNA	corr_RIF
bta-miR-92b	*CD44*	0.18979	POS	corr_miRNA	down_pathways
bta-miR-92b	*PIGS*	0.2556	POS	corr_miRNA	corr_hub
bta-miR-92b	*PRRX2*	0.21628	POS	corr_miRNA	down_TF
bta-miR-92b	*TNC*	0.21131	POS	corr_miRNA	down_trans_pathways
CD44	*CD86*	0.56728	POS	down_pathways	corr_RIF_hub
CD44	*COL11A1*	0.39695	POS	down_pathways	down_pathways
CD44	*COL12A1*	0.50116	POS	down_pathways	down_pathways
CD44	*COL21A1*	0.40465	POS	down_pathways	corr_pathways
CD44	*COL22A1*	0.39618	POS	down_pathways	down_trans_pathways
CD44	*COMP*	0.4391	POS	down_pathways	down_trans_pathways
CD44	*MMP16*	0.54236	POS	down_pathways	corr_down_hub_pathways
CD44	*PIGS*	0.41178	POS	down_pathways	corr_hub
CD44	*PLXDC1*	0.54544	POS	down_pathways	corr_hub
CD44	*PRRX2*	0.53604	POS	down_pathways	down_TF
CD44	*THBS4*	0.47498	POS	down_pathways	down_trans_pathways
CD44	*TNC*	0.62128	POS	down_pathways	down_trans_pathways
CD44	*ZIC3*	-0.22067	NEG	down_pathways	corr_TF
CD86	*COL21A1*	0.50852	POS	corr_RIF_hub	corr_pathways
CD86	*MMP16*	0.53103	POS	corr_RIF_hub	corr_down_hub_pathways
CD86	*PIGS*	0.37817	POS	corr_RIF_hub	corr_hub
CD86	*PLXDC1*	0.58135	POS	corr_RIF_hub	corr_hub
CD86	*TNC*	0.36261	POS	corr_RIF_hub	down_trans_pathways
CD86	*VDR*	0.47296	POS	corr_RIF_hub	corr_TF_RIF
CD86	*ZIC3*	-0.20872	NEG	corr_RIF_hub	corr_TF
COL11A1	*COL11A2*	0.50973	POS	down_pathways	corr_down_trans_pathways
COL11A1	*COL12A1*	0.83629	POS	down_pathways	down_pathways
COL11A1	*COL22A1*	0.8484	POS	down_pathways	down_trans_pathways
COL11A1	*COMP*	0.91524	POS	down_pathways	down_trans_pathways
COL11A1	*ITGA10*	0.65672	POS	down_pathways	down_pathways
COL11A1	*MMP16*	0.46702	POS	down_pathways	corr_down_hub_pathways
COL11A1	*PRRX2*	0.57971	POS	down_pathways	down_TF
COL11A1	*THBS4*	0.86932	POS	down_pathways	down_trans_pathways
COL11A1	*TNC*	0.5626	POS	down_pathways	down_trans_pathways
COL11A2	*COL22A1*	0.40066	POS	corr_down_trans_pathways	down_trans_pathways
COL11A2	*COMP*	0.44414	POS	corr_down_trans_pathways	down_trans_pathways
COL11A2	*ITGA10*	0.45989	POS	corr_down_trans_pathways	down_pathways
COL11A2	*PRRX2*	0.37351	POS	corr_down_trans_pathways	down_TF
COL11A2	*THBS4*	0.44015	POS	corr_down_trans_pathways	down_trans_pathways
COL12A1	*COL22A1*	0.73403	POS	down_pathways	down_trans_pathways
COL12A1	*COMP*	0.80686	POS	down_pathways	down_trans_pathways
COL12A1	*ITGA10*	0.53586	POS	down_pathways	down_pathways
COL12A1	*MMP16*	0.55788	POS	down_pathways	corr_down_hub_pathways
COL12A1	*PIGS*	0.28092	POS	down_pathways	corr_hub
COL12A1	*PRRX2*	0.59196	POS	down_pathways	down_TF
COL12A1	*THBS4*	0.8196	POS	down_pathways	down_trans_pathways
COL12A1	*TNC*	0.70665	POS	down_pathways	down_trans_pathways
COL21A1	*MMP16*	0.57002	POS	corr_pathways	corr_down_hub_pathways
COL21A1	*PIGS*	0.33165	POS	corr_pathways	corr_hub
COL21A1	*PLXDC1*	0.57358	POS	corr_pathways	corr_hub
COL21A1	*VDR*	0.36053	POS	corr_pathways	corr_TF_RIF

COL22A1	*COMP*	0.88862	POS	down_trans_pathways	down_trans_pathways
COL22A1	*ITGA10*	0.7114	POS	down_trans_pathways	down_pathways
COL22A1	*MMP16*	0.4187	POS	down_trans_pathways	corr_down_hub_pathways
COL22A1	*PRRX2*	0.52144	POS	down_trans_pathways	down_TF
COL22A1	*THBS4*	0.82451	POS	down_trans_pathways	down_trans_pathways
COL22A1	*TNC*	0.51025	POS	down_trans_pathways	down_trans_pathways
COMP	*ITGA10*	0.62139	POS	down_trans_pathways	down_pathways
COMP	*MMP16*	0.39517	POS	down_trans_pathways	corr_down_hub_pathways
COMP	*PRRX2*	0.61435	POS	down_trans_pathways	down_TF
COMP	*THBS4*	0.87442	POS	down_trans_pathways	down_trans_pathways
COMP	*TNC*	0.56269	POS	down_trans_pathways	down_trans_pathways
CTH	*ZIC3*	-0.17564	NEG	corr_RIF	corr_TF
ITGA10	*PRRX2*	0.45121	POS	down_pathways	down_TF
ITGA10	*THBS4*	0.61115	POS	down_pathways	down_trans_pathways
ITGA10	*TNC*	0.44769	POS	down_pathways	down_trans_pathways
MMP16	*PIGS*	0.45306	POS	corr_down_hub_pathways	corr_hub
MMP16	*PLXDC1*	0.54148	POS	corr_down_hub_pathways	corr_hub
MMP16	*PRRX2*	0.44498	POS	corr_down_hub_pathways	down_TF
MMP16	*THBS4*	0.49322	POS	corr_down_hub_pathways	down_trans_pathways
MMP16	*TNC*	0.55163	POS	corr_down_hub_pathways	down_trans_pathways
MMP16	*VDR*	0.35183	POS	corr_down_hub_pathways	corr_TF_RIF
MMP16	*ZIC3*	-0.2639	NEG	corr_down_hub_pathways	corr_TF
PIGS	*PLXDC1*	0.38315	POS	corr_hub	corr_hub
PIGS	*PRRX2*	0.31717	POS	corr_hub	down_TF
PIGS	*TNC*	0.3914	POS	corr_hub	down_trans_pathways
PIGS	*ZIC3*	-0.23213	NEG	corr_hub	corr_TF
PLXDC1	*VDR*	0.30439	POS	corr_hub	corr_TF_RIF
PRRX2	*THBS4*	0.63399	POS	down_TF	down_trans_pathways
PRRX2	*TNC*	0.64906	POS	down_TF	down_trans_pathways
PRRX2	*ZIC3*	-0.28348	NEG	down_TF	corr_TF
THBS4	*TNC*	0.61094	POS	down_trans_pathways	down_trans_pathways

Fe

**Origin**	**Target**	**Correlation value**	**Correlation type**	**Origin attributes**	**Target attributes**
ALAD	bta-miR-25	0.26203	POS	Corr_hub	Corr_RIF_miRNA
ALAD	bta-miR-378c	0.21704	POS	Corr_hub	Corr_RIF_miRNA
ALAD	*FASN*	-0.21106	NEG	Corr_hub	up_trans_pathway
ALAD	*HPCAL4*	0.42843	POS	Corr_hub	Corr_down_hub
ALAD	*HSPA6*	0.34633	POS	Corr_hub	up_TF_pathway
ALAD	*MYLK3*	0.37818	POS	Corr_hub	Corr_down_hub
ALAD	*PLIN5*	0.71483	POS	Corr_hub	down_pathway
ALAD	*SLC16A3*	0.29498	POS	Corr_hub	down_pathway
ALAD	*SLC27A6*	0.37818	POS	Corr_hub	down_pathway
ALAD	*TAP1*	0.28155	POS	Corr_hub	down_trans_pathway
ALAD	*TENM4*	-0.23274	NEG	Corr_hub	Corr_hub
bta-miR-127	bta-miR-25	0.14718	POS	Corr_miRNA	Corr_RIF_miRNA
bta-miR-127	bta-miR-532	0.18216	POS	Corr_miRNA	Corr_miRNA
bta-miR-127	*MT1A*	0.19933	POS	Corr_miRNA	up_TF
bta-miR-127	*PLCB2*	0.19909	POS	Corr_miRNA	Corr_trans_hub
bta-miR-127	*SLC16A3*	0.22506	POS	Corr_miRNA	down_pathway
bta-miR-181a	bta-miR-378c	0.37697	POS	Corr_miRNA	Corr_RIF_miRNA
bta-miR-181a	bta-miR-532	0.30467	POS	Corr_miRNA	Corr_miRNA

bta-miR-181a	*HSPA6*	0.24723	POS	Corr_miRNA	up_TF_pathway
bta-miR-25	bta-miR-378c	0.748	POS	Corr_RIF_miRNA	Corr_RIF_miRNA
bta-miR-25	bta-miR-532	0.68557	POS	Corr_RIF_miRNA	Corr_miRNA
bta-miR-25	*PLIN5*	0.17075	POS	Corr_RIF_miRNA	down_pathway
bta-miR-378c	bta-miR-532	0.65989	POS	Corr_RIF_miRNA	Corr_miRNA
bta-miR-378c	*C3*	-0.11905	NEG	Corr_RIF_miRNA	up_pathway
bta-miR-378c	*SLC27A6*	0.20604	POS	Corr_RIF_miRNA	down_pathway
bta-miR-532	*THRSP*	0.1665	POS	Corr_miRNA	up_trans_pathway
C3	*MMRN1*	0.4251	POS	up_pathway	up_TF
C3	*MT1A*	0.38708	POS	up_pathway	up_TF
C3	*PLCB2*	0.28483	POS	up_pathway	Corr_trans_hub
C3	*THRSP*	0.16244	POS	up_pathway	up_trans_pathway
FASN	*MMRN1*	0.28087	POS	up_trans_pathway	up_TF
FASN	*MT1A*	0.24793	POS	up_trans_pathway	up_TF
FASN	*TENM4*	0.52456	POS	up_trans_pathway	Corr_hub
FASN	*THRSP*	0.88294	POS	up_trans_pathway	up_trans_pathway
HES1	*HPCAL4*	0.26395	POS	down_TF	Corr_down_hub
HES1	*SLC16A3*	0.24259	POS	down_TF	down_pathway
HPCAL4	*MT1A*	-0.21551	NEG	Corr_down_hub	up_TF
HPCAL4	*MYLK3*	0.47043	POS	Corr_down_hub	Corr_down_hub
HPCAL4	*PLIN5*	0.49341	POS	Corr_down_hub	down_pathway
HPCAL4	*SLC16A3*	0.4999	POS	Corr_down_hub	down_pathway
HPCAL4	*SLC27A6*	0.41246	POS	Corr_down_hub	down_pathway
HPCAL4	*TENM4*	-0.17565	NEG	Corr_down_hub	Corr_hub
HPCAL4	*THRSP*	-0.21298	NEG	Corr_down_hub	up_trans_pathway
HSPA6	*PLCB2*	0.15599	POS	up_TF_pathway	Corr_trans_hub
MMRN1	*MYLK3*	-0.21045	NEG	up_TF	Corr_down_hub
MMRN1	*SLC27A6*	-0.17624	NEG	up_TF	down_pathway
MMRN1	*TENM4*	0.28434	POS	up_TF	Corr_hub
MT1A	*PLCB2*	0.26604	POS	up_TF	Corr_trans_hub
MT1A	*SLC27A6*	-0.19979	NEG	up_TF	down_pathway
MT1A	*THRSP*	0.21467	POS	up_TF	up_trans_pathway
MYLK3	*PLIN5*	0.33344	POS	Corr_down_hub	down_pathway
MYLK3	*SLC27A6*	0.47734	POS	Corr_down_hub	down_pathway
PLIN5	*TENM4*	-0.25144	NEG	down_pathway	Corr_hub
SLC16A3	*TAP1*	0.29067	POS	down_pathway	down_trans_pathway
SLC27A6	*TENM4*	-0.1988	NEG	down_pathway	Corr_hub
TAP1	*TENM4*	-0.26574	NEG	down_trans_pathway	Corr_hub
TENM4	*THRSP*	0.43831	POS	Corr_hub	up_trans_pathway

Ca

**Origin**	**Target**	**Correlation value**	**Correlation type**	**Origin attributes**	**Target attributes**
ADAMTS12	*ADAMTS2*	0.60203	POS	down_pathway	down_pathway
ADAMTS12	*BGN*	0.45908	POS	down_pathway	down_pathway
ADAMTS12	bta-miR-133a	-0.2387	NEG	down_pathway	Corr_miRNA_hub
ADAMTS12	*C7*	0.27417	POS	down_pathway	Corr_hub
ADAMTS12	*CD44*	0.47868	POS	down_pathway	down_pathway
ADAMTS12	*COL5A1*	0.63145	POS	down_pathway	down_pathway
ADAMTS12	*COL5A2*	0.59279	POS	down_pathway	down_pathway
ADAMTS12	*DDR2*	0.42713	POS	down_pathway	down_pathway
ADAMTS12	*EMILIN1*	0.54637	POS	down_pathway	down_pathway
ADAMTS12	*LUM*	0.43613	POS	down_pathway	down_pathway

ADAMTS12	*NID2*	0.68413	POS	down_pathway	down_pathway
ADAMTS12	*SDC3*	0.47236	POS	down_pathway	down_pathway
ADAMTS2	*BGN*	0.67169	POS	down_pathway	down_pathway
ADAMTS2	bta-miR-133a	-0.22859	NEG	down_pathway	down_pathway
ADAMTS2	bta-miR-369-3p	0.20735	POS	down_pathway	down_TF
ADAMTS2	*C1QB*	0.56418	POS	down_pathway	down_pathway
ADAMTS2	*C1QC*	0.5449	POS	down_pathway	down_pathway
ADAMTS2	*CD44*	0.6027	POS	down_pathway	down_pathway
ADAMTS2	*COL11A1*	0.34933	POS	down_pathway	down_pathway
ADAMTS2	*COL21A1*	0.47441	POS	down_pathway	down_pathway
ADAMTS2	*COL5A1*	0.81734	POS	down_pathway	down_pathway
ADAMTS2	*COL5A2*	0.75798	POS	down_pathway	down_pathway
ADAMTS2	*DDR2*	0.65521	POS	down_pathway	down_pathway
ADAMTS2	*EMILIN1*	0.51558	POS	down_pathway	down_pathway
ADAMTS2	*LOC786948*	0.33676	POS	down_pathway	down_pathway
ADAMTS2	*LUM*	0.63598	POS	down_pathway	down_TF
ADAMTS2	*MMP16*	0.73236	POS	down_pathway	down_pathway
ADAMTS2	*NCAM1*	0.3508	POS	down_pathway	Corr_TF_hub
ADAMTS2	*NID2*	0.64529	POS	down_pathway	down_pathway
ADAMTS2	*PCOLCE*	0.66409	POS	down_pathway	down_pathway
ADAMTS2	*PRRX2*	0.52925	POS	down_pathway	down_pathway
ADAMTS2	*SDC3*	0.60291	POS	down_pathway	down_pathway
ADAMTS2	*SPON2*	0.53794	POS	down_pathway	down_pathway
ADAMTS2	*TBL2*	0.2426	POS	down_pathway	Corr_TF_hub
ADAMTS2	*VDR*	0.29229	POS	down_pathway	Corr_TF_hub
ADAMTS2	*ZNF131*	-0.25091	NEG	down_pathway	down_pathway
BGN	bta-miR-133a	-0.2346	NEG	down_pathway	down_pathway
BGN	bta-miR-222	0.20074	POS	down_pathway	down_pathway
BGN	*C1QB*	0.48592	POS	down_pathway	down_pathway
BGN	*C1QC*	0.49882	POS	down_pathway	down_pathway
BGN	*CD44*	0.5998	POS	down_pathway	down_pathway
BGN	*COL11A1*	0.50256	POS	down_pathway	down_trans_pathway
BGN	*COL13A1*	0.41	POS	down_pathway	down_pathway
BGN	*COL5A1*	0.72655	POS	down_pathway	down_pathway
BGN	*COL5A2*	0.72828	POS	down_pathway	down_pathway
BGN	*DDR2*	0.43067	POS	down_pathway	down_trans_pathway
BGN	*EMILIN1*	0.55768	POS	down_pathway	down_pathway
BGN	*LUM*	0.63159	POS	down_pathway	down_pathway
BGN	*MIR29E*	-0.19653	NEG	down_pathway	Corr_miRNA
BGN	*MMP16*	0.6005	POS	down_pathway	down_pathway
BGN	*NCAM1*	0.36089	POS	down_pathway	down_TF_trans
BGN	*NID2*	0.5341	POS	down_pathway	down_pathway
BGN	*PCOLCE*	0.70481	POS	down_pathway	down_pathway
BGN	*PRRX2*	0.64281	POS	down_pathway	down_pathway
BGN	*SDC3*	0.4179	POS	down_pathway	down_TF_trans
BGN	*SPON2*	0.54657	POS	down_pathway	down_TF
BGN	*VDR*	0.29742	POS	down_pathway	down_pathway
BMF	bta-miR-222	0.18273	POS	Corr_hub	Corr_miRNA_hub
BMF	*CDK8*	-0.38432	NEG	Corr_hub	Corr_trans_hub
BMF	*DDR2*	0.30281	POS	Corr_hub	Corr_TF_hub
BMF	*ELL*	-0.37196	NEG	Corr_hub	Corr_trans_hub
BMF	*LOC786948*	0.29917	POS	Corr_hub	Corr_hub
BMF	*LPAR4*	0.33461	POS	Corr_hub	down_pathway

BMF	*MAFB*	0.41892	POS	Corr_hub	down_pathway
BMF	*MMP16*	0.28642	POS	Corr_hub	Corr_TF_hub
BMF	*OTOR*	0.26106	POS	Corr_hub	Corr_TF_hub
BMF	*ZNF131*	-0.27962	NEG	Corr_hub	up_TF
bta-miR-133a	bta-miR-193b	0.58688	POS	Corr_miRNA_hub	down_pathway
bta-miR-133a	*C1QB*	-0.23318	NEG	Corr_miRNA_hub	down_pathway
bta-miR-133a	*C1QC*	-0.25532	NEG	Corr_miRNA_hub	down_pathway
bta-miR-133a	*CD44*	-0.26122	NEG	Corr_miRNA_hub	Corr_miRNA
bta-miR-133a	*COL5A1*	-0.24584	NEG	Corr_miRNA_hub	Corr_TF_hub
bta-miR-133a	*COL5A2*	-0.31303	NEG	Corr_miRNA_hub	down_pathway
bta-miR-133a	*EMILIN1*	-0.23001	NEG	Corr_miRNA_hub	Corr_miRNA_hub
bta-miR-133a	*MMP16*	-0.21663	NEG	Corr_miRNA_hub	Corr_RIF_miRNA
bta-miR-133a	*NID2*	-0.24286	NEG	Corr_miRNA_hub	Corr_miRNA_hub
bta-miR-133a	*PCOLCE*	-0.2336	NEG	Corr_miRNA_hub	Corr_TF_hub
bta-miR-133a	*SPON2*	-0.23309	NEG	Corr_miRNA_hub	down_pathway
bta-miR-193b	bta-miR-222	-0.17872	NEG	Corr_miRNA	Corr_miRNA
bta-miR-193b	bta-miR-369-3p	-0.21416	NEG	Corr_miRNA	Corr_trans_hub
bta-miR-193b	bta-miR-92b	-0.26458	NEG	Corr_miRNA	down_pathway
bta-miR-193b	*C1QB*	-0.21766	NEG	Corr_miRNA	down_pathway
bta-miR-193b	*C1QC*	-0.25135	NEG	Corr_miRNA	Corr_TF_hub
bta-miR-193b	*CD44*	-0.25227	NEG	Corr_miRNA	down_trans_pathway
bta-miR-222	bta-miR-92b	0.19458	POS	Corr_miRNA	down_trans_pathway
bta-miR-222	*ELL*	-0.20922	NEG	Corr_miRNA	Corr_miRNA
bta-miR-222	*PRRX2*	0.22628	POS	Corr_miRNA	down_pathway
bta-miR-369-3p	*COL11A2*	0.19484	POS	Corr_RIF_miRNA	Corr_miRNA
bta-miR-369-3p	*ELL*	-0.36134	NEG	Corr_RIF_miRNA	up_TF
bta-miR-369-3p	*MIR29E*	-0.29256	NEG	Corr_RIF_miRNA	down_pathway
bta-miR-369-3p	*SPON2*	0.26343	POS	Corr_RIF_miRNA	Corr_TF_hub
bta-miR-369-3p	*ZNF131*	-0.2581	NEG	Corr_RIF_miRNA	down_trans_pathway
bta-miR-92b	*C1QB*	0.22666	POS	Corr_miRNA_hub	Corr_TF_hub
bta-miR-92b	*C1QC*	0.24678	POS	Corr_miRNA_hub	Corr_hub
bta-miR-92b	*COL5A2*	0.26217	POS	Corr_miRNA_hub	Corr_TF_hub
bta-miR-92b	*DDR2*	0.20441	POS	Corr_miRNA_hub	Corr_RIF_miRNA
bta-miR-92b	*PRRX2*	0.21628	POS	Corr_miRNA_hub	down_TF
bta-miR-92b	*TBL2*	0.25541	POS	Corr_miRNA_hub	Corr_RIF_hub
C1QB	*C1QC*	0.92502	POS	down_pathway	
C1QB	*CD44*	0.57187	POS	down_pathway	down_pathway
C1QB	*COL5A1*	0.50976	POS	down_pathway	down_pathway
C1QB	*COL5A2*	0.55117	POS	down_pathway	down_TF
C1QB	*DDR2*	0.4575	POS	down_pathway	down_pathway
C1QB	*EMILIN1*	0.45826	POS	down_pathway	down_pathway
C1QB	*LOC786948*	0.4206	POS	down_pathway	down_pathway
C1QB	*LUM*	0.53529	POS	down_pathway	down_TF
C1QB	*MMP16*	0.4654	POS	down_pathway	down_pathway
C1QB	*NFE2L3*	-0.33781	NEG	down_pathway	down_pathway
C1QB	*NID2*	0.45997	POS	down_pathway	down_pathway
C1QB	*PCOLCE*	0.55703	POS	down_pathway	down_pathway
C1QB	*PRRX2*	0.38989	POS	down_pathway	down_pathway
C1QB	*SDC3*	0.40461	POS	down_pathway	down_pathway
C1QB	*SPON2*	0.4289	POS	down_pathway	down_pathway
C1QB	*VDR*	0.30388	POS	down_pathway	Corr_hub
C1QB	*ZNF131*	-0.24523	NEG	down_pathway	down_pathway
C1QC	*CD44*	0.5838	POS	down_trans_pathway	down_pathway

C1QC	*COL5A1*	0.51573	POS	down_trans_pathway	down_TF_trans
C1QC	*COL5A2*	0.55676	POS	down_trans_pathway	down_pathway
C1QC	*DDR2*	0.44696	POS	down_trans_pathway	down_pathway
C1QC	*EMILIN1*	0.44081	POS	down_trans_pathway	down_pathway
C1QC	*LOC786948*	0.42985	POS	down_trans_pathway	down_pathway
C1QC	*LUM*	0.52716	POS	down_trans_pathway	down_TF
C1QC	*MMP16*	0.46266	POS	down_trans_pathway	down_pathway
C1QC	*NFE2L3*	-0.31873	NEG	down_trans_pathway	down_pathway
C1QC	*NID2*	0.42752	POS	down_trans_pathway	down_pathway
C1QC	*PCOLCE*	0.55551	POS	down_trans_pathway	down_pathway
C1QC	*PRRX2*	0.36607	POS	down_trans_pathway	down_pathway
C1QC	*SPON2*	0.48584	POS	down_trans_pathway	down_pathway
C1QC	*ZNF131*	-0.23349	NEG	down_trans_pathway	down_pathway
C7	*EMILIN1*	0.33243	POS	down_pathway	Corr_RIF_hub
C7	*NFE2L3*	-0.25938	NEG	down_pathway	down_pathway
C7	*NID2*	0.23957	POS	down_pathway	up_TF
C7	*OTOR*	0.25432	POS	down_pathway	Corr_hub
CD44	*COL11A1*	0.39695	POS	down_pathway	Corr_trans_hub
CD44	*COL13A1*	0.4345	POS	down_pathway	Corr_TF_hub
CD44	*COL21A1*	0.40465	POS	down_pathway	down_trans_pathway
CD44	*COL5A1*	0.70007	POS	down_pathway	down_pathway
CD44	*COL5A2*	0.71872	POS	down_pathway	down_trans_pathway
CD44	*EMILIN1*	0.59803	POS	down_pathway	down_pathway
CD44	*LOC786948*	0.35756	POS	down_pathway	down_pathway
CD44	*LUM*	0.61478	POS	down_pathway	down_pathway
CD44	*MIR29E*	-0.26088	NEG	down_pathway	down_pathway
CD44	*MMP16*	0.54236	POS	down_pathway	down_trans_pathway
CD44	*NCAM1*	0.46045	POS	down_pathway	down_pathway
CD44	*NFE2L3*	-0.26831	NEG	down_pathway	Corr_miRNA_hub
CD44	*NID2*	0.5994	POS	down_pathway	down_pathway
CD44	*PCOLCE*	0.62595	POS	down_pathway	down_pathway
CD44	*PRRX2*	0.53604	POS	down_pathway	down_pathway
CD44	*SDC3*	0.38027	POS	down_pathway	down_pathway
CD44	*SPON2*	0.62262	POS	down_pathway	down_pathway
CD44	*TBL2*	0.24581	POS	down_pathway	down_trans_pathway
CD44	*VDR*	0.26342	POS	down_pathway	down_pathway
CDK8	*ELL*	0.40114	POS	Corr_hub	down_pathway
CDK8	*LOC786948*	-0.25884	NEG	Corr_hub	Corr_TF_hub
CDK8	*LPAR4*	-0.32657	NEG	Corr_hub	down_pathway
CDK8	*MAFB*	-0.3464	NEG	Corr_hub	up_TF
CDK8	*MIR29E*	0.37763	POS	Corr_hub	down_pathway
CDK8	*NFE2L3*	0.24065	POS	Corr_hub	Corr_hub
CDK8	*ZNF131*	0.29256	POS	Corr_hub	down_pathway
COL11A1	*COL11A2*	0.50973	POS	down_pathway	down_pathway
COL11A1	*COL13A1*	0.72147	POS	down_pathway	down_pathway
COL11A1	*COL5A1*	0.34866	POS	down_pathway	down_pathway
COL11A1	*COL5A2*	0.43031	POS	down_pathway	down_pathway
COL11A1	*EMILIN1*	0.36645	POS	down_pathway	Corr_TF_hub
COL11A1	*MMP16*	0.46702	POS	down_pathway	down_pathway
COL11A1	*NCAM1*	0.57627	POS	down_pathway	down_pathway
COL11A1	*OTOR*	0.3131	POS	down_pathway	Corr_hub
COL11A1	*PCOLCE*	0.39019	POS	down_pathway	down_pathway
COL11A1	*PRRX2*	0.57971	POS	down_pathway	down_pathway

COL11A2	*COL13A1*	0.40593	POS	down_trans_pathway	down_trans_pathway
COL11A2	*OTOR*	0.31383	POS	down_trans_pathway	Corr_hub
COL11A2	*PRRX2*	0.37351	POS	down_trans_pathway	down_pathway
COL11A2	*TBL2*	0.30256	POS	down_trans_pathway	down_pathway
COL13A1	*COL5A2*	0.45156	POS	down_trans_pathway	down_pathway
COL13A1	*EMILIN1*	0.41214	POS	down_trans_pathway	down_pathway
COL13A1	*MMP16*	0.37799	POS	down_trans_pathway	down_pathway
COL13A1	*NCAM1*	0.49908	POS	down_trans_pathway	down_pathway
COL13A1	*OTOR*	0.35501	POS	down_trans_pathway	down_pathway
COL13A1	*PRRX2*	0.4844	POS	down_trans_pathway	down_pathway
COL13A1	*TBL2*	0.28346	POS	down_trans_pathway	down_pathway
COL21A1	*COL5A1*	0.55115	POS	down_pathway	down_pathway
COL21A1	*COL5A2*	0.4439	POS	down_pathway	Corr_TF_hub
COL21A1	*DDR2*	0.50236	POS	down_pathway	down_pathway
COL21A1	*ELL*	-0.44687	NEG	down_pathway	up_TF
COL21A1	*LUM*	0.67166	POS	down_pathway	down_pathway
COL21A1	*MAFB*	0.5209	POS	down_pathway	down_pathway
COL21A1	*MMP16*	0.57002	POS	down_pathway	down_pathway
COL21A1	*NCAM1*	0.37551	POS	down_pathway	down_pathway
COL21A1	*PCOLCE*	0.5363	POS	down_pathway	down_pathway
COL21A1	*SDC3*	0.31967	POS	down_pathway	down_TF_trans
COL21A1	*SPON2*	0.41993	POS	down_pathway	down_pathway
COL21A1	*VDR*	0.36053	POS	down_pathway	down_pathway
COL5A1	*COL5A2*	0.85293	POS	down_pathway	down_trans_pathway
COL5A1	*DDR2*	0.65301	POS	down_pathway	down_pathway
COL5A1	*EMILIN1*	0.5608	POS	down_pathway	down_pathway
COL5A1	*LUM*	0.69011	POS	down_pathway	down_pathway
COL5A1	*MMP16*	0.70367	POS	down_pathway	down_pathway
COL5A1	*NCAM1*	0.45117	POS	down_pathway	down_pathway
COL5A1	*NID2*	0.69851	POS	down_pathway	down_pathway
COL5A1	*PCOLCE*	0.70041	POS	down_pathway	down_pathway
COL5A1	*PRRX2*	0.5404	POS	down_pathway	down_pathway
COL5A1	*SDC3*	0.51215	POS	down_pathway	down_pathway
COL5A1	*SPON2*	0.58519	POS	down_pathway	Corr_miRNA
COL5A1	*VDR*	0.36719	POS	down_pathway	down_pathway
COL5A2	*DDR2*	0.59242	POS	down_pathway	down_pathway
COL5A2	*EMILIN1*	0.59508	POS	down_pathway	down_pathway
COL5A2	*LOC786948*	0.29757	POS	down_pathway	down_pathway
COL5A2	*LUM*	0.73873	POS	down_pathway	down_pathway
COL5A2	*MMP16*	0.63559	POS	down_pathway	down_pathway
COL5A2	*NCAM1*	0.49323	POS	down_pathway	down_trans_pathway
COL5A2	*NID2*	0.74018	POS	down_pathway	down_pathway
COL5A2	*PCOLCE*	0.65315	POS	down_pathway	down_pathway
COL5A2	*PRRX2*	0.54712	POS	down_pathway	down_pathway
COL5A2	*SDC3*	0.47398	POS	down_pathway	down_pathway
COL5A2	*SPON2*	0.57261	POS	down_pathway	down_pathway
COL5A2	*VDR*	0.27204	POS	down_pathway	down_pathway
DDR2	*ELL*	-0.36595	NEG	down_pathway	Corr_trans_hub
DDR2	*LOC786948*	0.30811	POS	down_pathway	Corr_TF_hub
DDR2	*LPAR4*	0.27567	POS	down_pathway	Corr_hub
DDR2	*LUM*	0.55839	POS	down_pathway	down_pathway
DDR2	*MAFB*	0.36345	POS	down_pathway	down_TF
DDR2	*MMP16*	0.60898	POS	down_pathway	down_pathway

DDR2	*NCAM1*	0.34622	POS	down_pathway	down_pathway
DDR2	*PCOLCE*	0.42748	POS	down_pathway	down_pathway
DDR2	*SDC3*	0.53774	POS	down_pathway	down_pathway
DDR2	*VDR*	0.30607	POS	down_pathway	down_pathway
ELL	*LOC786948*	-0.28854	NEG	Corr_trans_hub	Corr_TF_hub
ELL	*LUM*	-0.39771	NEG	Corr_trans_hub	Corr_TF_hub
ELL	*MAFB*	-0.54326	NEG	Corr_trans_hub	Corr_trans_hub
ELL	*MIR29E*	0.32981	POS	Corr_trans_hub	down_pathway
ELL	*MMP16*	-0.33093	NEG	Corr_trans_hub	down_pathway
ELL	*NCAM1*	-0.32781	NEG	Corr_trans_hub	Corr_RIF_hub
ELL	*PCOLCE*	-0.32174	NEG	Corr_trans_hub	up_TF
ELL	*VDR*	-0.38561	NEG	Corr_trans_hub	Corr_hub
ELL	*ZNF131*	0.43527	POS	Corr_trans_hub	down_pathway
EMILIN1	*LOC786948*	0.3551	POS	down_pathway	down_pathway
EMILIN1	*NCAM1*	0.35923	POS	down_pathway	Corr_TF_hub
EMILIN1	*NFE2L3*	-0.34932	NEG	down_pathway	down_TF_trans
EMILIN1	*NID2*	0.59086	POS	down_pathway	down_pathway
EMILIN1	*OTOR*	0.30476	POS	down_pathway	down_TF
EMILIN1	*PCOLCE*	0.5464	POS	down_pathway	down_pathway
EMILIN1	*PRRX2*	0.52258	POS	down_pathway	down_pathway
EMILIN1	*SPON2*	0.44141	POS	down_pathway	down_pathway
EMILIN1	*TBL2*	0.2569	POS	down_pathway	Corr_TF_hub
LOC786948	*LPAR4*	0.25657	POS	down_pathway	Corr_hub
LOC786948	*LUM*	0.38424	POS	down_pathway	down_TF
LOC786948	*MMP16*	0.29599	POS	down_pathway	Corr_TF_hub
LOC786948	*NFE2L3*	-0.41418	NEG	down_pathway	down_pathway
LOC786948	*OTOR*	0.25515	POS	down_pathway	Corr_hub
LOC786948	*PCOLCE*	0.47021	POS	down_pathway	down_pathway
LOC786948	*PRRX2*	0.30607	POS	down_pathway	Corr_TF_hub
LOC786948	*SPON2*	0.34139	POS	down_pathway	down_pathway
LPAR4	*MAFB*	0.31984	POS	Corr_RIF_hub	up_TF
LPAR4	*MMP16*	0.33709	POS	Corr_RIF_hub	down_pathway
LPAR4	*OTOR*	0.25666	POS	Corr_RIF_hub	Corr_hub
LUM	*MMP16*	0.64738	POS	down_pathway	down_pathway
LUM	*NCAM1*	0.476	POS	down_pathway	down_pathway
LUM	*NID2*	0.57781	POS	down_pathway	down_TF
LUM	*PCOLCE*	0.67807	POS	down_pathway	down_pathway
LUM	*SDC3*	0.43924	POS	down_pathway	down_pathway
LUM	*SPON2*	0.5022	POS	down_pathway	down_pathway
LUM	*VDR*	0.3102	POS	down_pathway	Corr_hub
MAFB	*MMP16*	0.36856	POS	down_TF_trans	down_TF
MAFB	*PCOLCE*	0.37871	POS	down_TF_trans	down_pathway
MAFB	*VDR*	0.26127	POS	down_TF_trans	down_pathway
MIR29E	*NFE2L3*	0.3222	POS	Corr_miRNA	Corr_miRNA
MIR29E	*PCOLCE*	-0.26033	NEG	Corr_miRNA	up_TF
MIR29E	*ZNF131*	0.31943	POS	Corr_miRNA	Corr_hub
MMP16	*NCAM1*	0.48157	POS	down_pathway	down_TF
MMP16	*NID2*	0.48611	POS	down_pathway	down_pathway
MMP16	*PCOLCE*	0.59088	POS	down_pathway	down_pathway
MMP16	*PRRX2*	0.44498	POS	down_pathway	down_pathway
MMP16	*SDC3*	0.45636	POS	down_pathway	down_pathway
MMP16	*SPON2*	0.46821	POS	down_pathway	down_pathway
MMP16	*TBL2*	0.27526	POS	down_pathway	Corr_RIF_hub

MMP16	*VDR*	0.35183	POS	down_pathway	Corr_hub
MMP16	*ZNF131*	-0.28335	NEG	down_pathway	Corr_TF_hub
NCAM1	*NID2*	0.37651	POS	down_pathway	Corr_miRNA
NCAM1	*OTOR*	0.29925	POS	down_pathway	Corr_hub
NCAM1	*PCOLCE*	0.42505	POS	down_pathway	down_pathway
NFE2L3	*PCOLCE*	-0.33526	NEG	up_TF	down_pathway
NFE2L3	*SPON2*	-0.29391	NEG	up_TF	Corr_miRNA
NFE2L3	*ZNF131*	0.44446	POS	up_TF	down_TF
NID2	*PCOLCE*	0.52148	POS	down_pathway	down_TF
NID2	*SDC3*	0.569	POS	down_pathway	down_pathway
NID2	*SPON2*	0.38028	POS	down_pathway	down_pathway
NID2	*TBL2*	0.31948	POS	down_pathway	down_pathway
PCOLCE	*PRRX2*	0.55277	POS	down_pathway	down_pathway
PCOLCE	*SPON2*	0.70278	POS	down_pathway	down_pathway
PCOLCE	*VDR*	0.2446	POS	down_pathway	Corr_hub
PRRX2	*SPON2*	0.53911	POS	down_TF	down_TF
SDC3	*TBL2*	0.31495	POS	down_pathway	Corr_TF_hub
SPON2	*VDR*	0.25906	POS	down_pathway	Corr_hub
THSD7B	*VDR*	0.23242	POS	Corr_pathway	down_pathway

Se

**Origin**	**Target**	**Correlation value**	**Correlation type**	**Origin attributes**	**Target attributes**
B3GNT5	bta-miR-2285bl	-0.22275	NEG	Corr_hub	Corr_miRNA
B3GNT5	bta-miR-2285co	-0.22275	NEG	Corr_hub	Corr_miRNA
B3GNT5	bta-miR-2285q	-0.22007	NEG	Corr_hub	Corr_miRNA
B3GNT5	bta-miR-425-5p	-0.20216	NEG	Corr_hub	Corr_miRNA
B3GNT5	*COL12A1*	0.2591	POS	Corr_hub	down_pathways
B3GNT5	*HARS*	-0.29062	NEG	Corr_hub	Corr_RIF
B3GNT5	*LOC101907941*	0.35999	POS	Corr_hub	Corr_hub
B3GNT5	*RFX3*	0.3497	POS	Corr_hub	Corr_TF
B3GNT5	*TTC21A*	0.29773	POS	Corr_hub	Corr_RIF
B3GNT5	*ZDBF2*	0.35741	POS	Corr_hub	Corr_RIF
B3GNT5	*ZDHHC17*	0.33057	POS	Corr_hub	Corr_hub
bta-miR-2285bl	bta-miR-2285co	1	POS	Corr_miRNA	Corr_miRNA
bta-miR-2285bl	bta-miR-2285q	0.35281	POS	Corr_miRNA	Corr_miRNA
bta-miR-2285bl	bta-miR-411c-5p	0.32726	POS	Corr_miRNA	Corr_miRNA
bta-miR-2285bl	bta-miR-425-5p	0.16937	POS	Corr_miRNA	Corr_miRNA
bta-miR-2285bl	*LOC101907941*	-0.23616	NEG	Corr_miRNA	Corr_hub
bta-miR-2285bl	*LOC112442312*	-0.27456	NEG	Corr_miRNA	Corr_RIF
bta-miR-2285co	bta-miR-2285q	0.35281	POS	Corr_miRNA	Corr_miRNA
bta-miR-2285co	bta-miR-411c-5p	0.32726	POS	Corr_miRNA	Corr_miRNA
bta-miR-2285co	bta-miR-425-5p	0.16937	POS	Corr_miRNA	Corr_miRNA
bta-miR-2285co	*LOC101907941*	-0.23616	NEG	Corr_miRNA	Corr_hub
bta-miR-2285co	*LOC112442312*	-0.27456	NEG	Corr_miRNA	Corr_RIF
bta-miR-2285q	*COMP*	0.14962	POS	Corr_miRNA	down_trans_pathway
bta-miR-2285q	*HARS*	0.18369	POS	Corr_miRNA	Corr_RIF
bta-miR-2285q	*LOC101907941*	-0.20243	NEG	Corr_miRNA	Corr_hub
bta-miR-411c-5p	*HARS*	0.36182	POS	Corr_miRNA	Corr_RIF
bta-miR-411c-5p	*LOC101907941*	-0.25711	NEG	Corr_miRNA	Corr_hub

bta-miR-411c-5p	*TEF*	0.28007	POS	Corr_miRNA	Corr_TF_RIF
bta-miR-425-5p	*COL12A1*	-0.23077	NEG	Corr_miRNA	down_pathways
bta-miR-425-5p	*TEF*	0.23074	POS	Corr_miRNA	Corr_TF_RIF
bta-miR-425-5p	*ZDBF2*	-0.23657	NEG	Corr_miRNA	Corr_RIF
COL12A1	*COMP*	0.80686	POS	down_pathways	down_trans_pathway
COMP	*DTWD1*	-0.20516	NEG	down_trans_pathway	Corr_RIF_trans
COMP	*ZDHHC17*	-0.25903	NEG	down_trans_pathway	Corr_hub
DTWD1	*HARS*	-0.47568	NEG	Corr_RIF_trans	Corr_RIF
DTWD1	*LOC101907941*	0.44639	POS	Corr_RIF_trans	Corr_hub
DTWD1	*LOC112442312*	0.30752	POS	Corr_RIF_trans	Corr_RIF
DTWD1	*PDK3*	-0.24357	NEG	Corr_RIF_trans	Corr_RIF
DTWD1	*TTC21A*	0.2565	POS	Corr_RIF_trans	Corr_RIF
DTWD1	*ZDHHC17*	0.32468	POS	Corr_RIF_trans	Corr_hub
HARS	*LOC112442312*	-0.29246	NEG	Corr_RIF	Corr_RIF
HARS	*TEF*	0.32789	POS	Corr_RIF	Corr_TF_RIF
HARS	*TTC21A*	-0.29023	NEG	Corr_RIF	Corr_RIF
HARS	*ZDBF2*	-0.32652	NEG	Corr_RIF	Corr_RIF
HARS	*ZDHHC17*	-0.39084	NEG	Corr_RIF	Corr_hub
LOC101907941	*LOC112442312*	0.2731	POS	Corr_hub	Corr_RIF
LOC101907941	*RFX3*	0.36447	POS	Corr_hub	Corr_TF
LOC101907941	*TTC21A*	0.35571	POS	Corr_hub	Corr_RIF
LOC101907941	*ZDBF2*	0.42047	POS	Corr_hub	Corr_RIF
LOC101907941	*ZDHHC17*	0.3672	POS	Corr_hub	Corr_hub
LOC112442312	*PDK3*	-0.29305	NEG	Corr_RIF	Corr_RIF
LOC112442312	*RFX3*	0.34973	POS	Corr_RIF	Corr_TF
LOC112442312	*TTC21A*	0.37688	POS	Corr_RIF	Corr_RIF
PDK3	*RFX3*	-0.25699	NEG	Corr_RIF	Corr_TF
PDK3	*TTC21A*	-0.31104	NEG	Corr_RIF	Corr_RIF
PDK3	*ZDBF2*	-0.23919	NEG	Corr_RIF	Corr_RIF
RFX3	*TTC21A*	0.34307	POS	Corr_TF	Corr_RIF
RFX3	*ZDBF2*	0.33798	POS	Corr_TF	Corr_RIF
RFX3	*ZDHHC17*	0.37051	POS	Corr_TF	Corr_hub
TEF	*ZDBF2*	-0.2515	NEG	Corr_TF_RIF	Corr_RIF
TEF	*ZDHHC17*	-0.37387	NEG	Corr_TF_RIF	Corr_hub
TTC21A	*ZDBF2*	0.28816	POS	Corr_RIF	Corr_RIF
ZDBF2	*ZDHHC17*	0.30766	POS	Corr_RIF	Corr_hub

K

**Origin**	**Target**	**Correlation value**	**Correlation type**	**Origin attributes**	**Target attributes**
ADA2	*ANGPTL2*	0.35405	POS	Corr_hub	Corr_down_hub
ADA2	*ARAP1*	0.42043	POS	Corr_hub	Corr_hub
ADA2	*ARHGAP30*	0.62805	POS	Corr_hub	Corr_hub
ADA2	bta-miR-500	0.17628	POS	Corr_hub	Corr_miRNA
ADA2	*CD44*	0.4937	POS	Corr_hub	down_pathways
ADA2	*CD86*	0.5016	POS	Corr_hub	Corr_RIF_hub
ADA2	*CREM*	-0.25042	NEG	Corr_hub	up_TF
ADA2	*FCGR3A*	0.53873	POS	Corr_hub	down_pathways
ADA2	*MMP16*	0.46043	POS	Corr_hub	Corr_RIF_hub_pathways
ADA2	*PRRX2*	0.38604	POS	Corr_hub	down_TF
ADA2	*TNC*	0.37287	POS	Corr_hub	down_trans_pathways
ADA2	*VDR*	0.39319	POS	Corr_hub	Corr_TF
ADAM12	*CD44*	0.40463	POS	down_pathways	down_pathways

ADAM12	*COL11A1*	0.65195	POS	down_pathways	down_pathways
ADAM12	*COL21A1*	0.31051	POS	down_pathways	Corr_pathways
ADAM12	*COL22A1*	0.62413	POS	down_pathways	down_trans_pathways
ADAM12	*COMP*	0.59696	POS	down_pathways	down_trans_pathways
ADAM12	*ITGA10*	0.58824	POS	down_pathways	down_pathways
ADAM12	*MMP16*	0.48484	POS	down_pathways	Corr_RIF_hub_pathways
ADAM12	*RNF34*	-0.33693	NEG	down_pathways	Corr_RIF
ADAM12	*THBS4*	0.62637	POS	down_pathways	down_trans_pathways
ADAM12	*TNC*	0.48486	POS	down_pathways	down_trans_pathways
ANGPTL2	*ARAP1*	0.53772	POS	Corr_down_hub	Corr_hub
ANGPTL2	*ARHGAP30*	0.397	POS	Corr_down_hub	Corr_hub
ANGPTL2	*CD44*	0.49225	POS	Corr_down_hub	down_pathways
ANGPTL2	*CD86*	0.5459	POS	Corr_down_hub	Corr_RIF_hub
ANGPTL2	*COL21A1*	0.67041	POS	Corr_down_hub	Corr_pathways
ANGPTL2	*COL22A1*	0.27257	POS	Corr_down_hub	down_trans_pathways
ANGPTL2	*CREM*	-0.29314	NEG	Corr_down_hub	up_TF
ANGPTL2	*FCGR3A*	0.4017	POS	Corr_down_hub	down_pathways
ANGPTL2	*MMP16*	0.63372	POS	Corr_down_hub	Corr_RIF_hub_pathways
ANGPTL2	*PRRX2*	0.3377	POS	Corr_down_hub	down_TF
ANGPTL2	*RNF34*	-0.37953	NEG	Corr_down_hub	Corr_RIF
ANGPTL2	*THBS4*	0.37246	POS	Corr_down_hub	down_trans_pathways
ANGPTL2	*TNC*	0.40863	POS	Corr_down_hub	down_trans_pathways
ANGPTL2	*VDR*	0.39249	POS	Corr_down_hub	Corr_TF
ARAP1	*ARHGAP30*	0.51489	POS	Corr_hub	Corr_hub
ARAP1	bta-miR-92b	0.17023	POS	Corr_hub	Corr_RIF_miRNA
ARAP1	*CD44*	0.36823	POS	Corr_hub	down_pathways
ARAP1	*CD86*	0.4028	POS	Corr_hub	Corr_RIF_hub
ARAP1	*COL11A1*	0.34854	POS	Corr_hub	down_pathways
ARAP1	*COL21A1*	0.41349	POS	Corr_hub	Corr_pathways
ARAP1	*COL22A1*	0.37363	POS	Corr_hub	down_trans_pathways
ARAP1	*COMP*	0.33452	POS	Corr_hub	down_trans_pathways
ARAP1	*CREM*	-0.29229	NEG	Corr_hub	up_TF
ARAP1	*FCGR3A*	0.41209	POS	Corr_hub	down_pathways
ARAP1	*ITGA10*	0.31531	POS	Corr_hub	down_pathways
ARAP1	*MMP16*	0.52931	POS	Corr_hub	Corr_RIF_hub_pathways
ARAP1	*PRRX2*	0.42059	POS	Corr_hub	down_TF
ARAP1	*RNF34*	-0.47661	NEG	Corr_hub	Corr_RIF
ARAP1	*THBS4*	0.4325	POS	Corr_hub	down_trans_pathways
ARAP1	*TNC*	0.48848	POS	Corr_hub	down_trans_pathways
ARAP1	*VDR*	0.37873	POS	Corr_hub	Corr_TF
ARHGAP30	bta-miR-92b	0.1913	POS	Corr_hub	Corr_RIF_miRNA
ARHGAP30	*CD44*	0.52823	POS	Corr_hub	down_pathways
ARHGAP30	*CD86*	0.66218	POS	Corr_hub	Corr_RIF_hub
ARHGAP30	*FCGR3A*	0.67343	POS	Corr_hub	down_pathways
ARHGAP30	*MMP16*	0.46874	POS	Corr_hub	Corr_RIF_hub_pathways
ARHGAP30	*PRRX2*	0.39351	POS	Corr_hub	down_TF
ARHGAP30	*RNF34*	-0.38796	NEG	Corr_hub	Corr_RIF
ARHGAP30	*TNC*	0.39982	POS	Corr_hub	down_trans_pathways
ARHGAP30	*VDR*	0.37471	POS	Corr_hub	Corr_TF
bta-miR-130b	bta-miR-92b	0.18846	POS	Corr_miRNA	Corr_RIF_miRNA
bta-miR-130b	*RNF34*	-0.18585	NEG	Corr_miRNA	Corr_RIF
bta-miR-500	*ITGA10*	-0.21082	NEG	Corr_miRNA	down_pathways
bta-miR-92b	*CD44*	0.18979	POS	Corr_RIF_miRNA	down_pathways

bta-miR-92b	*COL21A1*	0.17499	POS	Corr_RIF_miRNA	Corr_pathways
bta-miR-92b	*MMP16*	0.18661	POS	Corr_RIF_miRNA	Corr_RIF_hub_pathways
bta-miR-92b	*PRRX2*	0.21628	POS	Corr_RIF_miRNA	down_TF
bta-miR-92b	*TNC*	0.21131	POS	Corr_RIF_miRNA	down_trans_pathways
CD44	*CD86*	0.56728	POS	down_pathways	Corr_RIF_hub
CD44	*COL11A1*	0.39695	POS	down_pathways	down_pathways
CD44	*COL21A1*	0.40465	POS	down_pathways	Corr_pathways
CD44	*COL22A1*	0.39618	POS	down_pathways	down_trans_pathways
CD44	*COMP*	0.4391	POS	down_pathways	down_trans_pathways
CD44	*FCGR3A*	0.4618	POS	down_pathways	down_pathways
CD44	*MMP16*	0.54236	POS	down_pathways	Corr_RIF_hub_pathways
CD44	*PRRX2*	0.53604	POS	down_pathways	down_TF
CD44	*THBS4*	0.47498	POS	down_pathways	down_trans_pathways
CD44	*TNC*	0.62128	POS	down_pathways	down_trans_pathways
CD44	*ZIC3*	-0.22067	NEG	down_pathways	Corr_TF
CD86	*COL21A1*	0.50852	POS	Corr_RIF_hub	Corr_pathways
CD86	*FCGR3A*	0.50597	POS	Corr_RIF_hub	down_pathways
CD86	*MMP16*	0.53103	POS	Corr_RIF_hub	Corr_RIF_hub_pathways
CD86	*RNF34*	-0.41476	NEG	Corr_RIF_hub	Corr_RIF
CD86	*TNC*	0.36261	POS	Corr_RIF_hub	down_trans_pathways
CD86	*VDR*	0.47296	POS	Corr_RIF_hub	Corr_TF
CD86	*ZIC3*	-0.20872	NEG	Corr_RIF_hub	Corr_TF
COL11A1	*COL22A1*	0.8484	POS	down_pathways	down_trans_pathways
COL11A1	*COMP*	0.91524	POS	down_pathways	down_trans_pathways
COL11A1	*ITGA10*	0.65672	POS	down_pathways	down_pathways
COL11A1	*MMP16*	0.46702	POS	down_pathways	Corr_RIF_hub_pathways
COL11A1	*PRRX2*	0.57971	POS	down_pathways	down_TF
COL11A1	*THBS4*	0.86932	POS	down_pathways	down_trans_pathways
COL11A1	*TNC*	0.5626	POS	down_pathways	down_trans_pathways
COL21A1	*MMP16*	0.57002	POS	Corr_pathways	Corr_RIF_hub_pathways
COL21A1	*RNF34*	-0.49719	NEG	Corr_pathways	Corr_RIF
COL21A1	*VDR*	0.36053	POS	Corr_pathways	Corr_TF
COL22A1	*COMP*	0.88862	POS	down_trans_pathways	down_trans_pathways
COL22A1	*ITGA10*	0.7114	POS	down_trans_pathways	down_pathways
COL22A1	*MMP16*	0.4187	POS	down_trans_pathways	Corr_RIF_hub_pathways
COL22A1	*PRRX2*	0.52144	POS	down_trans_pathways	down_TF
COL22A1	*RNF34*	-0.30794	NEG	down_trans_pathways	Corr_RIF
COL22A1	*THBS4*	0.82451	POS	down_trans_pathways	down_trans_pathways
COL22A1	*TNC*	0.51025	POS	down_trans_pathways	down_trans_pathways
COMP	*ITGA10*	0.62139	POS	down_trans_pathways	down_pathways
COMP	*MMP16*	0.39517	POS	down_trans_pathways	Corr_RIF_hub_pathways
COMP	*PRRX2*	0.61435	POS	down_trans_pathways	down_TF
COMP	*THBS4*	0.87442	POS	down_trans_pathways	down_trans_pathways
COMP	*TNC*	0.56269	POS	down_trans_pathways	down_trans_pathways
CREM	*MMP16*	-0.23325	NEG	up_TF	Corr_RIF_hub_pathways
CREM	*THBS4*	-0.24267	NEG	up_TF	down_trans_pathways
CREM	*VDR*	-0.22453	NEG	up_TF	Corr_TF
FCGR3A	*MMP16*	0.41479	POS	down_pathways	Corr_RIF_hub_pathways
FCGR3A	*TNC*	0.33341	POS	down_pathways	down_trans_pathways
ITGA10	*PRRX2*	0.45121	POS	down_pathways	down_TF
ITGA10	*RNF34*	-0.2434	NEG	down_pathways	Corr_RIF
ITGA10	*THBS4*	0.61115	POS	down_pathways	down_trans_pathways
ITGA10	*TNC*	0.44769	POS	down_pathways	down_trans_pathways

MMP16	*PRRX2*	0.44498	POS	Corr_RIF_hub_pathways	down_TF
MMP16	*RNF34*	-0.50322	NEG	Corr_RIF_hub_pathways	Corr_RIF
MMP16	*THBS4*	0.49322	POS	Corr_RIF_hub_pathways	down_trans_pathways
MMP16	*TNC*	0.55163	POS	Corr_RIF_hub_pathways	down_trans_pathways
MMP16	*VDR*	0.35183	POS	Corr_RIF_hub_pathways	Corr_TF
MMP16	*ZIC3*	-0.2639	NEG	Corr_RIF_hub_pathways	Corr_TF
PRRX2	*THBS4*	0.63399	POS	down_TF	down_trans_pathways
PRRX2	*TNC*	0.64906	POS	down_TF	down_trans_pathways
PRRX2	*ZIC3*	-0.28348	NEG	down_TF	Corr_TF
RNF34	*VDR*	-0.2846	NEG	Corr_RIF	Corr_TF
THBS4	*TNC*	0.61094	POS	down_trans_pathways	down_trans_pathways

Na

**Origin**	**Target**	**Correlation value**	**Correlation type**	**Origin attributes**	**Target attributes**
ADA2	*ARAP1*	0.42043	POS	Corr_hub	Corr_hub
ADA2	bta-miR-22-5p	-0.13427	NEG	Corr_hub	Corr_miRNA
ADA2	*CD44*	0.4937	POS	Corr_hub	down_pathways
ADA2	*COL12A1*	0.34418	POS	Corr_hub	down_pathways
ADA2	*COL18A1*	0.34985	POS	Corr_hub	down_pathways
ADA2	*COL21A1*	0.32274	POS	Corr_hub	Corr_pathways
ADA2	*MMP16*	0.46043	POS	Corr_hub	Corr_hub_pathways
ADA2	*PRRX2*	0.38604	POS	Corr_hub	down_TF
ADA2	*TNC*	0.37287	POS	Corr_hub	down_trans_pathways
ADA2	*VDR*	0.39319	POS	Corr_hub	Corr_TF_RIF
ARAP1	*CAMKK1*	0.37721	POS	Corr_hub	Corr_RIF
ARAP1	*CD44*	0.36823	POS	Corr_hub	down_pathways
ARAP1	*COL11A1*	0.34854	POS	Corr_hub	down_pathways
ARAP1	*COL12A1*	0.40061	POS	Corr_hub	down_pathways
ARAP1	*COL18A1*	0.48117	POS	Corr_hub	down_pathways
ARAP1	*COL21A1*	0.41349	POS	Corr_hub	Corr_pathways
ARAP1	*COL22A1*	0.37363	POS	Corr_hub	down_trans_pathways
ARAP1	*COMP*	0.33452	POS	Corr_hub	down_trans_pathways
ARAP1	*ITGA10*	0.31531	POS	Corr_hub	down_pathways
ARAP1	*LOXL3*	0.32665	POS	Corr_hub	Corr_trans_pathways
ARAP1	*MMP16*	0.52931	POS	Corr_hub	Corr_hub_pathways
ARAP1	*PRRX2*	0.42059	POS	Corr_hub	down_TF
ARAP1	*THBS4*	0.4325	POS	Corr_hub	down_trans_pathways
ARAP1	*TNC*	0.48848	POS	Corr_hub	down_trans_pathways
ARAP1	*VDR*	0.37873	POS	Corr_hub	Corr_TF_RIF
bta-miR-125a	bta-miR-92b	0.36714	POS	Corr_RIF_miRNA	Corr_miRNA
bta-miR-125a	*ITGA10*	0.153	POS	Corr_RIF_miRNA	down_pathways
bta-miR-125a	*VMAC*	-0.09683	NEG	Corr_RIF_miRNA	Corr_RIF_trans
bta-miR-125a	*WDPCP*	0.1263	POS	Corr_RIF_miRNA	Corr_RIF_trans
bta-miR-130b	bta-miR-365-3p	-0.14531	NEG	Corr_miRNA	Corr_miRNA
bta-miR-130b	bta-miR-92b	0.18846	POS	Corr_miRNA	Corr_miRNA
bta-miR-130b	*CDKN3*	0.23004	POS	Corr_miRNA	Corr_RIF
bta-miR-130b	*COL18A1*	0.17597	POS	Corr_miRNA	down_pathways
bta-miR-22-5p	bta-miR-365-3p	0.12967	POS	Corr_miRNA	Corr_miRNA
bta-miR-22-5p	*CDKN3*	-0.21977	NEG	Corr_miRNA	Corr_RIF
bta-miR-22-5p	*ITGA10*	-0.11961	NEG	Corr_miRNA	down_pathways
bta-miR-22-5p	*WDPCP*	-0.13938	NEG	Corr_miRNA	Corr_RIF_trans
bta-miR-365-3p	*VMAC*	-0.234	NEG	Corr_miRNA	Corr_RIF_trans

bta-miR-92b	*COL18A1*	0.2622	POS	Corr_miRNA	down_pathways
bta-miR-92b	*LOXL3*	0.25573	POS	Corr_miRNA	Corr_trans_pathways
bta-miR-92b	*PRRX2*	0.21628	POS	Corr_miRNA	down_TF
bta-miR-92b	*TNC*	0.21131	POS	Corr_miRNA	down_trans_pathways
CAMKK1	*COL21A1*	0.28651	POS	Corr_RIF	Corr_pathways
CAMKK1	*COL22A1*	0.31348	POS	Corr_RIF	down_trans_pathways
CAMKK1	*ITGA10*	0.32288	POS	Corr_RIF	down_pathways
CAMKK1	*MMP16*	0.43273	POS	Corr_RIF	Corr_hub_pathways
CAMKK1	*TNC*	0.29579	POS	Corr_RIF	down_trans_pathways
CD44	*CENPE*	0.29282	POS	down_pathways	Corr_RIF
CD44	*COL11A1*	0.39695	POS	down_pathways	down_pathways
CD44	*COL12A1*	0.50116	POS	down_pathways	down_pathways
CD44	*COL18A1*	0.55564	POS	down_pathways	down_pathways
CD44	*COL21A1*	0.40465	POS	down_pathways	Corr_pathways
CD44	*COL22A1*	0.39618	POS	down_pathways	down_trans_pathways
CD44	*COMP*	0.4391	POS	down_pathways	down_trans_pathways
CD44	*LOXL3*	0.31712	POS	down_pathways	Corr_trans_pathways
CD44	*MMP16*	0.54236	POS	down_pathways	Corr_hub_pathways
CD44	*PRRX2*	0.53604	POS	down_pathways	down_TF
CD44	*THBS4*	0.47498	POS	down_pathways	down_trans_pathways
CD44	*TNC*	0.62128	POS	down_pathways	down_trans_pathways
CD44	*ZIC3*	-0.22067	NEG	down_pathways	Corr_TF
CDKN3	*COL21A1*	0.22794	POS	Corr_RIF	Corr_pathways
CDKN3	*VMAC*	0.2189	POS	Corr_RIF	Corr_RIF_trans
CENPE	*COL18A1*	0.30483	POS	Corr_RIF	down_pathways
CENPE	*COL21A1*	0.28536	POS	Corr_RIF	Corr_pathways
CENPE	*ITGA10*	0.29739	POS	Corr_RIF	down_pathways
CENPE	*MMP16*	0.24064	POS	Corr_RIF	Corr_hub_pathways
CENPE	*TNC*	0.23169	POS	Corr_RIF	down_trans_pathways
COL11A1	*COL12A1*	0.83629	POS	down_pathways	down_pathways
COL11A1	*COL22A1*	0.8484	POS	down_pathways	down_trans_pathways
COL11A1	*COMP*	0.91524	POS	down_pathways	down_trans_pathways
COL11A1	*ITGA10*	0.65672	POS	down_pathways	down_pathways
COL11A1	*MMP16*	0.46702	POS	down_pathways	Corr_hub_pathways
COL11A1	*PRRX2*	0.57971	POS	down_pathways	down_TF
COL11A1	*THBS4*	0.86932	POS	down_pathways	down_trans_pathways
COL11A1	*TNC*	0.5626	POS	down_pathways	down_trans_pathways
COL12A1	*COL18A1*	0.45534	POS	down_pathways	down_pathways
COL12A1	*COL22A1*	0.73403	POS	down_pathways	down_trans_pathways
COL12A1	*COMP*	0.80686	POS	down_pathways	down_trans_pathways
COL12A1	*ITGA10*	0.53586	POS	down_pathways	down_pathways
COL12A1	*MMP16*	0.55788	POS	down_pathways	Corr_hub_pathways
COL12A1	*PRRX2*	0.59196	POS	down_pathways	down_TF
COL12A1	*THBS4*	0.8196	POS	down_pathways	down_trans_pathways
COL12A1	*TNC*	0.70665	POS	down_pathways	down_trans_pathways
COL18A1	*COL21A1*	0.43911	POS	down_pathways	Corr_pathways
COL18A1	*COL22A1*	0.40831	POS	down_pathways	down_trans_pathways
COL18A1	*ITGA10*	0.34102	POS	down_pathways	down_pathways
COL18A1	*LOXL3*	0.49161	POS	down_pathways	Corr_trans_pathways
COL18A1	*MMP16*	0.49981	POS	down_pathways	Corr_hub_pathways
COL18A1	*PRRX2*	0.59877	POS	down_pathways	down_TF
COL18A1	*THBS4*	0.49388	POS	down_pathways	down_trans_pathways
COL18A1	*TNC*	0.67805	POS	down_pathways	down_trans_pathways

COL18A1	*VDR*	0.28961	POS	down_pathways	Corr_TF_RIF
COL18A1	*VMAC*	0.2601	POS	down_pathways	Corr_RIF_trans
COL18A1	*ZIC3*	-0.21718	NEG	down_pathways	Corr_TF
COL21A1	*MMP16*	0.57002	POS	Corr_pathways	Corr_hub_pathways
COL21A1	*VDR*	0.36053	POS	Corr_pathways	Corr_TF_RIF
COL21A1	*VMAC*	0.33072	POS	Corr_pathways	Corr_RIF_trans
COL22A1	*COMP*	0.88862	POS	down_trans_pathways	down_trans_pathways
COL22A1	*ITGA10*	0.7114	POS	down_trans_pathways	down_pathways
COL22A1	*MMP16*	0.4187	POS	down_trans_pathways	Corr_hub_pathways
COL22A1	*PRRX2*	0.52144	POS	down_trans_pathways	down_TF
COL22A1	*THBS4*	0.82451	POS	down_trans_pathways	down_trans_pathways
COL22A1	*TNC*	0.51025	POS	down_trans_pathways	down_trans_pathways
COMP	*ITGA10*	0.62139	POS	down_trans_pathways	down_pathways
COMP	*MMP16*	0.39517	POS	down_trans_pathways	Corr_hub_pathways
COMP	*PRRX2*	0.61435	POS	down_trans_pathways	down_TF
COMP	*THBS4*	0.87442	POS	down_trans_pathways	down_trans_pathways
COMP	*TNC*	0.56269	POS	down_trans_pathways	down_trans_pathways
ITGA10	*PRRX2*	0.45121	POS	down_pathways	down_TF
ITGA10	*THBS4*	0.61115	POS	down_pathways	down_trans_pathways
ITGA10	*TNC*	0.44769	POS	down_pathways	down_trans_pathways
LOXL3	*MMP16*	0.34055	POS	Corr_trans_pathways	Corr_hub_pathways
LOXL3	*PRRX2*	0.37333	POS	Corr_trans_pathways	down_TF
LOXL3	*THBS4*	0.33181	POS	Corr_trans_pathways	down_trans_pathways
LOXL3	*TNC*	0.41046	POS	Corr_trans_pathways	down_trans_pathways
LOXL3	*ZIC3*	-0.21593	NEG	Corr_trans_pathways	Corr_TF
MMP16	*PRRX2*	0.44498	POS	Corr_hub_pathways	down_TF
MMP16	*THBS4*	0.49322	POS	Corr_hub_pathways	down_trans_pathways
MMP16	*TNC*	0.55163	POS	Corr_hub_pathways	down_trans_pathways
MMP16	*VDR*	0.35183	POS	Corr_hub_pathways	Corr_TF_RIF
MMP16	*ZIC3*	-0.2639	NEG	Corr_hub_pathways	Corr_TF
PRRX2	*THBS4*	0.63399	POS	down_TF	down_trans_pathways
PRRX2	*TNC*	0.64906	POS	down_TF	down_trans_pathways
PRRX2	*ZIC3*	-0.28348	NEG	down_TF	Corr_TF
THBS4	*TNC*	0.61094	POS	down_trans_pathways	down_trans_pathways
VDR	*WDPCP*	0.19512	POS	Corr_TF_RIF	Corr_RIF_trans
WDPCP	*ZIC3*	-0.20815	NEG	Corr_RIF_trans	Corr_TF

Cu

**Origin**	**Target**	**Correlation value**	**Correlation type**	**Origin attributes**	**Target attributes**
ACACA	*ADAM12*	0.39135	POS	down_pathway	down_pathway
ACACA	*ADIPOQ*	0.69483	POS	down_pathway	down_pathway
ACACA	*CD44*	0.39952	POS	down_pathway	down_pathway
ACACA	*COL12A1*	0.47683	POS	down_pathway	down_pathway
ACACA	*COL18A1*	0.54106	POS	down_pathway	down_pathway
ACACA	*COL22A1*	0.41128	POS	down_pathway	down_trans_pathway
ACACA	*COL5A2*	0.56861	POS	down_pathway	down_pathway
ACACA	*COMP*	0.37713	POS	down_pathway	down_trans_pathway
ACACA	*EBF1*	0.61166	POS	down_pathway	down_TF
ACACA	*ELOVL5*	0.70981	POS	down_pathway	down_trans_pathway
ACACA	*ELOVL6*	0.83635	POS	down_pathway	down_pathway
ACACA	*FASN*	0.83521	POS	down_pathway	down_trans_pathway
ACACA	*GNAI1*	0.77254	POS	down_pathway	down_pathway

ACACA	*ITGA10*	0.35281	POS	down_pathway	down_pathway
ACACA	*LEP*	0.78849	POS	down_pathway	down_pathway
ACACA	*MKX*	0.39623	POS	down_pathway	down_TF
ACACA	*PCK2*	0.76171	POS	down_pathway	down_pathway
ACACA	*PLIN1*	0.72349	POS	down_pathway	down_pathway
ACACA	*PRRX2*	0.36829	POS	down_pathway	down_TF
ACACA	*PTGIR*	0.38688	POS	down_pathway	down_trans_pathway
ACACA	*RGS7*	0.34611	POS	down_pathway	corr_hub
ACACA	*SCD*	0.72632	POS	down_pathway	down_pathway
ACACA	*THBS1*	0.6109	POS	down_pathway	down_pathway
ACACA	*THBS4*	0.39229	POS	down_pathway	down_trans_pathway
ACACA	*TINF2*	-0.28525	NEG	down_pathway	Corr_hub
ACACA	*TNC*	0.51513	POS	down_pathway	down_trans_pathway
ADAM12	*CD44*	0.40463	POS	down_pathway	down_pathway
ADAM12	*COL11A1*	0.65195	POS	down_pathway	down_pathway
ADAM12	*COL11A2*	0.34259	POS	down_pathway	down_trans_pathway
ADAM12	*COL12A1*	0.62039	POS	down_pathway	down_pathway
ADAM12	*COL18A1*	0.39984	POS	down_pathway	down_pathway
ADAM12	*COL22A1*	0.62413	POS	down_pathway	down_trans_pathway
ADAM12	*COL5A2*	0.56362	POS	down_pathway	down_pathway
ADAM12	*COMP*	0.59696	POS	down_pathway	down_trans_pathway
ADAM12	*EBF1*	0.48618	POS	down_pathway	down_TF
ADAM12	*ELOVL5*	0.38741	POS	down_pathway	down_trans_pathway
ADAM12	*ELOVL6*	0.35568	POS	down_pathway	down_pathway
ADAM12	*GNAI1*	0.37752	POS	down_pathway	down_pathway
ADAM12	*ITGA10*	0.58824	POS	down_pathway	down_pathway
ADAM12	*LUM*	0.41996	POS	down_pathway	down_pathway
ADAM12	*MEST*	0.60539	POS	down_pathway	corr_down_hub
ADAM12	*MKX*	0.57413	POS	down_pathway	down_TF
ADAM12	*NUCB2*	0.3408	POS	down_pathway	corr_hub
ADAM12	*PTGIR*	0.64227	POS	down_pathway	down_trans_pathway
ADAM12	*SCD*	0.32089	POS	down_pathway	down_pathway
ADAM12	*THBS1*	0.48719	POS	down_pathway	down_pathway
ADAM12	*THBS4*	0.62637	POS	down_pathway	down_trans_pathway
ADAM12	*TNC*	0.48486	POS	down_pathway	down_trans_pathway
ADAM12	*TNFRSF11B*	0.41583	POS	down_pathway	corr_trans_hub
ADAMTS2	*CD44*	0.6027	POS	down_pathway	down_pathway
ADAMTS2	*COL12A1*	0.55185	POS	down_pathway	down_pathway
ADAMTS2	*COL18A1*	0.57455	POS	down_pathway	down_pathway
ADAMTS2	*COL5A2*	0.75798	POS	down_pathway	down_pathway
ADAMTS2	*DIAPH3*	0.44198	POS	down_pathway	corr_hub
ADAMTS2	*EBF1*	0.55747	POS	down_pathway	down_TF
ADAMTS2	*LOC530929*	-0.19747	NEG	down_pathway	corr_RIF
ADAMTS2	*LUM*	0.63598	POS	down_pathway	down_pathway
ADAMTS2	*MEST*	0.4482	POS	down_pathway	corr_down_hub
ADAMTS2	*PRRX2*	0.52925	POS	down_pathway	down_TF
ADAMTS2	*PTGIR*	0.40315	POS	down_pathway	down_trans_pathway
ADAMTS2	*SGCE*	0.54261	POS	down_pathway	corr_hub
ADAMTS2	*THBS1*	0.4782	POS	down_pathway	down_pathway
ADAMTS2	*THBS4*	0.44508	POS	down_pathway	down_trans_pathway
ADAMTS2	*TINF2*	-0.3197	NEG	down_pathway	Corr_hub
ADAMTS2	*TNC*	0.60397	POS	down_pathway	down_trans_pathway
ADIPOQ	*bta-miR-193b*	-0.19277	NEG	down_pathway	corr_miRNA_hub

ADIPOQ	*CD44*	0.39371	POS	down_pathway	down_pathway
ADIPOQ	*COL12A1*	0.37338	POS	down_pathway	down_pathway
ADIPOQ	*COL18A1*	0.58575	POS	down_pathway	down_pathway
ADIPOQ	*COL22A1*	0.44634	POS	down_pathway	down_trans_pathway
ADIPOQ	*COL5A2*	0.44248	POS	down_pathway	down_pathway
ADIPOQ	*EBF1*	0.51717	POS	down_pathway	down_TF
ADIPOQ	*ELOVL5*	0.61324	POS	down_pathway	down_trans_pathway
ADIPOQ	*ELOVL6*	0.76064	POS	down_pathway	down_pathway
ADIPOQ	*FASN*	0.70297	POS	down_pathway	down_trans_pathway
ADIPOQ	*GNAI1*	0.83027	POS	down_pathway	down_pathway
ADIPOQ	*LEP*	0.80693	POS	down_pathway	down_pathway
ADIPOQ	*MKX*	0.35157	POS	down_pathway	down_TF
ADIPOQ	*PCK2*	0.77798	POS	down_pathway	down_pathway
ADIPOQ	*PLIN1*	0.95922	POS	down_pathway	down_pathway
ADIPOQ	*PRRX2*	0.36001	POS	down_pathway	down_TF
ADIPOQ	*RASL11A*	0.15715	POS	down_pathway	corr_RIF
ADIPOQ	*SCD*	0.57718	POS	down_pathway	down_pathway
ADIPOQ	*SGCE*	0.28932	POS	down_pathway	corr_hub
ADIPOQ	*THBS1*	0.53303	POS	down_pathway	down_pathway
ADIPOQ	*TINF2*	-0.3174	NEG	down_pathway	Corr_hub
ADIPOQ	*TNC*	0.41624	POS	down_pathway	down_trans_pathway
BHLHE22	bta-miR-365-5p	-0.17367	NEG	corr_TF	corr_miRNA
BHLHE22	*CD44*	0.21404	POS	corr_TF	down_pathway
BHLHE22	*COL12A1*	0.16799	POS	corr_TF	down_pathway
BHLHE22	*COL5A2*	0.18036	POS	corr_TF	down_pathway
BHLHE22	*DIAPH3*	0.21716	POS	corr_TF	corr_hub
BHLHE22	*EBF1*	0.17519	POS	corr_TF	down_TF
BHLHE22	*THBS4*	0.20097	POS	corr_TF	down_trans_pathway
bta-miR-1468	bta-miR-150	0.41728	POS	corr_miRNA	corr_miRNA
bta-miR-1468	bta-miR-193b	-0.35982	NEG	corr_miRNA	corr_miRNA_hub
bta-miR-1468	*LUM*	-0.13908	NEG	corr_miRNA	down_pathway
bta-miR-150	bta-miR-193b	-0.26695	NEG	corr_miRNA	corr_miRNA_hub
bta-miR-150	bta-miR-493	0.24899	POS	corr_miRNA	corr_miRNA
bta-miR-150	*NUCB2*	-0.17081	NEG	corr_miRNA	corr_hub
bta-miR-193b	bta-miR-365-5p	0.15542	POS	corr_miRNA_hub	corr_miRNA
bta-miR-193b	*CD44*	-0.25227	NEG	corr_miRNA_hub	down_pathway
bta-miR-193b	*COL5A2*	-0.19257	NEG	corr_miRNA_hub	down_pathway
bta-miR-193b	*ELOVL5*	-0.28507	NEG	corr_miRNA_hub	down_trans_pathway
bta-miR-193b	*GNAI1*	-0.23338	NEG	corr_miRNA_hub	down_pathway
bta-miR-193b	*P4HA3*	-0.22785	NEG	corr_miRNA_hub	down_pathway
bta-miR-365-5p	*RASL11A*	0.12282	POS	corr_miRNA	corr_RIF
bta-miR-365-5p	*SGCE*	-0.1933	NEG	corr_miRNA	corr_hub
bta-miR-493	*COL11A2*	0.18641	POS	corr_miRNA	down_trans_pathway
bta-miR-493	*LOC518768*	-0.26385	NEG	corr_miRNA	corr_RIF
bta-miR-493	*LOC530929*	0.27111	POS	corr_miRNA	corr_RIF
CD44	*COL11A1*	0.39695	POS	down_pathway	down_pathway
CD44	*COL12A1*	0.50116	POS	down_pathway	down_pathway
CD44	*COL18A1*	0.55564	POS	down_pathway	down_pathway
CD44	*COL22A1*	0.39618	POS	down_pathway	down_trans_pathway
CD44	*COL5A2*	0.71872	POS	down_pathway	down_pathway
CD44	*COMP*	0.4391	POS	down_pathway	down_trans_pathway
CD44	*DIAPH3*	0.39266	POS	down_pathway	corr_hub
CD44	*ELOVL5*	0.4623	POS	down_pathway	down_trans_pathway

CD44	*ELOVL6*	0.42851	POS	down_pathway	down_pathway
CD44	*FASN*	0.34523	POS	down_pathway	down_trans_pathway
CD44	*GNAI1*	0.44996	POS	down_pathway	down_pathway
CD44	*LEP*	0.34536	POS	down_pathway	down_pathway
CD44	*LOC784127*	0.231	POS	down_pathway	corr_RIF
CD44	*LUM*	0.61478	POS	down_pathway	down_pathway
CD44	*MEST*	0.49759	POS	down_pathway	corr_down_hub
CD44	*MKX*	0.41899	POS	down_pathway	down_TF
CD44	*NUCB2*	0.34573	POS	down_pathway	corr_hub
CD44	*PCK2*	0.41228	POS	down_pathway	down_pathway
CD44	*PLIN1*	0.34457	POS	down_pathway	down_pathway
CD44	*PRRX2*	0.53604	POS	down_pathway	down_TF
CD44	*PTGIR*	0.46566	POS	down_pathway	down_trans_pathway
CD44	*SGCE*	0.52758	POS	down_pathway	corr_hub
CD44	*THBS1*	0.4821	POS	down_pathway	down_pathway
CD44	*THBS4*	0.47498	POS	down_pathway	down_trans_pathway
CD44	*TINF2*	-0.3483	NEG	down_pathway	Corr_hub
CD44	*TNC*	0.62128	POS	down_pathway	down_trans_pathway
COL11A1	*COL11A2*	0.50973	POS	down_pathway	down_trans_pathway
COL11A1	*COL12A1*	0.83629	POS	down_pathway	down_pathway
COL11A1	*COL22A1*	0.8484	POS	down_pathway	down_trans_pathway
COL11A1	*COMP*	0.91524	POS	down_pathway	down_trans_pathway
COL11A1	*DIAPH3*	0.39593	POS	down_pathway	corr_hub
COL11A1	*ELOVL6*	0.34846	POS	down_pathway	down_pathway
COL11A1	*FASN*	0.31449	POS	down_pathway	down_trans_pathway
COL11A1	*ITGA10*	0.65672	POS	down_pathway	down_pathway
COL11A1	*MEST*	0.59973	POS	down_pathway	corr_down_hub
COL11A1	*MKX*	0.84326	POS	down_pathway	down_TF
COL11A1	*NUCB2*	0.45017	POS	down_pathway	corr_hub
COL11A1	*PRRX2*	0.57971	POS	down_pathway	down_TF
COL11A1	*PTGIR*	0.70835	POS	down_pathway	down_trans_pathway
COL11A1	*SCD*	0.35228	POS	down_pathway	down_pathway
COL11A1	*THBS1*	0.62968	POS	down_pathway	down_pathway
COL11A1	*THBS4*	0.86932	POS	down_pathway	down_trans_pathway
COL11A1	*TNC*	0.5626	POS	down_pathway	down_trans_pathway
COL11A1	*TNFRSF11B*	0.57514	POS	down_pathway	corr_trans_hub
COL11A2	*COL22A1*	0.40066	POS	down_trans_pathway	down_trans_pathway
COL11A2	*COMP*	0.44414	POS	down_trans_pathway	down_trans_pathway
COL11A2	*ITGA10*	0.45989	POS	down_trans_pathway	down_pathway
COL11A2	*LOC530929*	0.21157	POS	down_trans_pathway	corr_RIF
COL11A2	*LOC784127*	0.25927	POS	down_trans_pathway	corr_RIF
COL11A2	*MEST*	0.41446	POS	down_trans_pathway	corr_down_hub
COL11A2	*MKX*	0.3852	POS	down_trans_pathway	down_TF
COL11A2	*PRRX2*	0.37351	POS	down_trans_pathway	down_TF
COL11A2	*PTGIR*	0.45619	POS	down_trans_pathway	down_trans_pathway
COL11A2	*THBS4*	0.44015	POS	down_trans_pathway	down_trans_pathway
COL11A2	*TNFRSF11B*	0.45072	POS	down_trans_pathway	corr_trans_hub
COL12A1	*COL18A1*	0.45534	POS	down_pathway	down_pathway
COL12A1	*COL22A1*	0.73403	POS	down_pathway	down_trans_pathway
COL12A1	*COL5A2*	0.66109	POS	down_pathway	down_pathway
COL12A1	*COMP*	0.80686	POS	down_pathway	down_trans_pathway
COL12A1	*DIAPH3*	0.51382	POS	down_pathway	corr_hub
COL12A1	*EBF1*	0.62502	POS	down_pathway	down_TF

COL12A1	*ELOVL5*	0.43092	POS	down_pathway	down_trans_pathway
COL12A1	*ELOVL6*	0.47456	POS	down_pathway	down_pathway
COL12A1	*FASN*	0.42522	POS	down_pathway	down_trans_pathway
COL12A1	*GNAI1*	0.42119	POS	down_pathway	down_pathway
COL12A1	*ITGA10*	0.53586	POS	down_pathway	down_pathway
COL12A1	*LEP*	0.37929	POS	down_pathway	down_pathway
COL12A1	*LUM*	0.47659	POS	down_pathway	down_pathway
COL12A1	*MEST*	0.60645	POS	down_pathway	corr_down_hub
COL12A1	*MKX*	0.80536	POS	down_pathway	down_TF
COL12A1	*NUCB2*	0.37451	POS	down_pathway	corr_hub
COL12A1	*P4HA3*	0.36693	POS	down_pathway	down_pathway
COL12A1	*PCK2*	0.41195	POS	down_pathway	down_pathway
COL12A1	*PLIN1*	0.3653	POS	down_pathway	down_pathway
COL12A1	*PRRX2*	0.59196	POS	down_pathway	down_TF
COL12A1	*PTGIR*	0.6598	POS	down_pathway	down_trans_pathway
COL12A1	*RGS7*	0.29923	POS	down_pathway	corr_hub
COL12A1	*SCD*	0.45246	POS	down_pathway	down_pathway
COL12A1	*SGCE*	0.40412	POS	down_pathway	corr_hub
COL12A1	*THBS1*	0.77757	POS	down_pathway	down_pathway
COL12A1	*THBS4*	0.8196	POS	down_pathway	down_trans_pathway
COL12A1	*TNC*	0.70665	POS	down_pathway	down_trans_pathway
COL12A1	*TNFRSF11B*	0.51338	POS	down_pathway	corr_trans_hub
COL18A1	*COL22A1*	0.40831	POS	down_pathway	down_trans_pathway
COL18A1	*COL5A2*	0.67625	POS	down_pathway	down_pathway
COL18A1	*EBF1*	0.61681	POS	down_pathway	down_TF
COL18A1	*ELOVL5*	0.59471	POS	down_pathway	down_trans_pathway
COL18A1	*ELOVL6*	0.51138	POS	down_pathway	down_pathway
COL18A1	*GNAI1*	0.69544	POS	down_pathway	down_pathway
COL18A1	*ITGA10*	0.34102	POS	down_pathway	down_pathway
COL18A1	*LEP*	0.54988	POS	down_pathway	down_pathway
COL18A1	*LUM*	0.60643	POS	down_pathway	down_pathway
COL18A1	*MEST*	0.426	POS	down_pathway	corr_down_hub
COL18A1	*MKX*	0.43671	POS	down_pathway	down_TF
COL18A1	*PCK2*	0.50316	POS	down_pathway	down_pathway
COL18A1	*PLIN1*	0.58934	POS	down_pathway	down_pathway
COL18A1	*PRRX2*	0.59877	POS	down_pathway	down_TF
COL18A1	*PTGIR*	0.45348	POS	down_pathway	down_trans_pathway
COL18A1	*RASL11A*	0.20959	POS	down_pathway	corr_RIF
COL18A1	*SCD*	0.43294	POS	down_pathway	down_pathway
COL18A1	*SGCE*	0.44031	POS	down_pathway	corr_hub
COL18A1	*THBS1*	0.53108	POS	down_pathway	down_pathway
COL18A1	*THBS4*	0.49388	POS	down_pathway	down_trans_pathway
COL18A1	*TINF2*	-0.34877	NEG	down_pathway	Corr_hub
COL18A1	*TNC*	0.67805	POS	down_pathway	down_trans_pathway
COL22A1	*COMP*	0.88862	POS	down_trans_pathway	down_trans_pathway
COL22A1	*EBF1*	0.43658	POS	down_trans_pathway	down_TF
COL22A1	*ELOVL5*	0.3348	POS	down_trans_pathway	down_trans_pathway
COL22A1	*ELOVL6*	0.39953	POS	down_trans_pathway	down_pathway
COL22A1	*FASN*	0.38489	POS	down_trans_pathway	down_trans_pathway
COL22A1	*GNAI1*	0.45894	POS	down_trans_pathway	down_pathway
COL22A1	*ITGA10*	0.7114	POS	down_trans_pathway	down_pathway
COL22A1	*LEP*	0.39984	POS	down_trans_pathway	down_pathway
COL22A1	*MKX*	0.71844	POS	down_trans_pathway	down_TF

COL22A1	*NUCB2*	0.34655	POS	down_trans_pathway	corr_hub
COL22A1	*PCK2*	0.44028	POS	down_trans_pathway	down_pathway
COL22A1	*PLIN1*	0.45013	POS	down_trans_pathway	down_pathway
COL22A1	*PRRX2*	0.52144	POS	down_trans_pathway	down_TF
COL22A1	*PTGIR*	0.66055	POS	down_trans_pathway	down_trans_pathway
COL22A1	*SCD*	0.37272	POS	down_trans_pathway	down_pathway
COL22A1	*THBS1*	0.60916	POS	down_trans_pathway	down_pathway
COL22A1	*THBS4*	0.82451	POS	down_trans_pathway	down_trans_pathway
COL22A1	*TNC*	0.51025	POS	down_trans_pathway	down_trans_pathway
COL22A1	*TNFRSF11B*	0.50164	POS	down_trans_pathway	corr_trans_hub
COL5A2	*COMP*	0.47155	POS	down_pathway	down_trans_pathway
COL5A2	*DIAPH3*	0.51084	POS	down_pathway	corr_hub
COL5A2	*EBF1*	0.72974	POS	down_pathway	down_TF
COL5A2	*ELOVL5*	0.61556	POS	down_pathway	down_trans_pathway
COL5A2	*ELOVL6*	0.57332	POS	down_pathway	down_pathway
COL5A2	*FASN*	0.46793	POS	down_pathway	down_trans_pathway
COL5A2	*GNAI1*	0.5492	POS	down_pathway	down_pathway
COL5A2	*LEP*	0.46701	POS	down_pathway	down_pathway
COL5A2	*LOC784127*	0.17874	POS	down_pathway	corr_RIF
COL5A2	*LUM*	0.73873	POS	down_pathway	down_pathway
COL5A2	*MEST*	0.62102	POS	down_pathway	corr_down_hub
COL5A2	*MKX*	0.51319	POS	down_pathway	down_TF
COL5A2	*NUCB2*	0.32453	POS	down_pathway	corr_hub
COL5A2	*P4HA3*	0.33676	POS	down_pathway	down_pathway
COL5A2	*PCK2*	0.48111	POS	down_pathway	down_pathway
COL5A2	*PLIN1*	0.42188	POS	down_pathway	down_pathway
COL5A2	*PRRX2*	0.54712	POS	down_pathway	down_TF
COL5A2	*PTGIR*	0.5388	POS	down_pathway	down_trans_pathway
COL5A2	*SCD*	0.5047	POS	down_pathway	down_pathway
COL5A2	*SGCE*	0.55253	POS	down_pathway	corr_hub
COL5A2	*THBS1*	0.63497	POS	down_pathway	down_pathway
COL5A2	*THBS4*	0.5417	POS	down_pathway	down_trans_pathway
COL5A2	*TINF2*	-0.41668	NEG	down_pathway	Corr_hub
COL5A2	*TNC*	0.70207	POS	down_pathway	down_trans_pathway
COMP	*DIAPH3*	0.39992	POS	down_trans_pathway	corr_hub
COMP	*ELOVL5*	0.37065	POS	down_trans_pathway	down_trans_pathway
COMP	*ELOVL6*	0.36162	POS	down_trans_pathway	down_pathway
COMP	*FASN*	0.34654	POS	down_trans_pathway	down_trans_pathway
COMP	*GNAI1*	0.3677	POS	down_trans_pathway	down_pathway
COMP	*ITGA10*	0.62139	POS	down_trans_pathway	down_pathway
COMP	*LEP*	0.34059	POS	down_trans_pathway	down_pathway
COMP	*MEST*	0.50138	POS	down_trans_pathway	corr_down_hub
COMP	*MKX*	0.79315	POS	down_trans_pathway	down_TF
COMP	*NUCB2*	0.35389	POS	down_trans_pathway	corr_hub
COMP	*P4HA3*	0.38628	POS	down_trans_pathway	down_pathway
COMP	*PCK2*	0.39047	POS	down_trans_pathway	down_pathway
COMP	*PRRX2*	0.61435	POS	down_trans_pathway	down_TF
COMP	*PTGIR*	0.6862	POS	down_trans_pathway	down_trans_pathway
COMP	*SCD*	0.37468	POS	down_trans_pathway	down_pathway
COMP	*THBS1*	0.61137	POS	down_trans_pathway	down_pathway
COMP	*THBS4*	0.87442	POS	down_trans_pathway	down_trans_pathway
COMP	*TNC*	0.56269	POS	down_trans_pathway	down_trans_pathway
COMP	*TNFRSF11B*	0.56944	POS	down_trans_pathway	corr_trans_hub

DIAPH3	*LUM*	0.40369	POS	corr_hub	down_pathway
DIAPH3	*MEST*	0.36131	POS	corr_hub	corr_down_hub
DIAPH3	*PRRX2*	0.32041	POS	corr_hub	down_TF
DIAPH3	*PTGIR*	0.37192	POS	corr_hub	down_trans_pathway
DIAPH3	*THBS1*	0.3832	POS	corr_hub	down_pathway
DIAPH3	*THBS4*	0.42449	POS	corr_hub	down_trans_pathway
DIAPH3	*TINF2*	-0.32601	NEG	corr_hub	Corr_hub
DIAPH3	*TNC*	0.45343	POS	corr_hub	down_trans_pathway
EBF1	*ELOVL5*	0.58808	POS	down_TF	down_trans_pathway
EBF1	*ELOVL6*	0.55398	POS	down_TF	down_pathway
EBF1	*FASN*	0.481	POS	down_TF	down_trans_pathway
EBF1	*GNAI1*	0.60609	POS	down_TF	down_pathway
EBF1	*ITGA10*	0.38725	POS	down_TF	down_pathway
EBF1	*LEP*	0.5168	POS	down_TF	down_pathway
EBF1	*MEST*	0.42403	POS	down_TF	corr_down_hub
EBF1	*MKX*	0.49041	POS	down_TF	down_TF
EBF1	*P4HA3*	0.30251	POS	down_TF	down_pathway
EBF1	*PCK2*	0.43225	POS	down_TF	down_pathway
EBF1	*PLIN1*	0.52505	POS	down_TF	down_pathway
EBF1	*PRRX2*	0.42476	POS	down_TF	down_TF
EBF1	*PTGIR*	0.43319	POS	down_TF	down_trans_pathway
EBF1	*RGS7*	0.2795	POS	down_TF	corr_hub
EBF1	*SCD*	0.47747	POS	down_TF	down_pathway
EBF1	*SGCE*	0.36549	POS	down_TF	corr_hub
EBF1	*THBS1*	0.70549	POS	down_TF	down_pathway
EBF1	*THBS4*	0.49407	POS	down_TF	down_trans_pathway
EBF1	*TINF2*	-0.30445	NEG	down_TF	Corr_hub
EBF1	*TNC*	0.59461	POS	down_TF	down_trans_pathway
EBF1	*TNFRSF11B*	0.36604	POS	down_TF	corr_trans_hub
ELOVL5	*ELOVL6*	0.74965	POS	down_trans_pathway	down_pathway
ELOVL5	*FASN*	0.73275	POS	down_trans_pathway	down_trans_pathway
ELOVL5	*GNAI1*	0.72009	POS	down_trans_pathway	down_pathway
ELOVL5	*LEP*	0.69848	POS	down_trans_pathway	down_pathway
ELOVL5	*LOC784127*	0.20645	POS	down_trans_pathway	corr_RIF
ELOVL5	*MKX*	0.46791	POS	down_trans_pathway	down_TF
ELOVL5	*P4HA3*	0.44994	POS	down_trans_pathway	down_pathway
ELOVL5	*PCK2*	0.7209	POS	down_trans_pathway	down_pathway
ELOVL5	*PLIN1*	0.58964	POS	down_trans_pathway	down_pathway
ELOVL5	*PRRX2*	0.49108	POS	down_trans_pathway	down_TF
ELOVL5	*PTGIR*	0.41843	POS	down_trans_pathway	down_trans_pathway
ELOVL5	*RASL11A*	0.23619	POS	down_trans_pathway	corr_RIF
ELOVL5	*SCD*	0.61993	POS	down_trans_pathway	down_pathway
ELOVL5	*THBS1*	0.53538	POS	down_trans_pathway	down_pathway
ELOVL5	*THBS4*	0.34961	POS	down_trans_pathway	down_trans_pathway
ELOVL5	*TINF2*	-0.28308	NEG	down_trans_pathway	Corr_hub
ELOVL5	*TNC*	0.50932	POS	down_trans_pathway	down_trans_pathway
ELOVL5	*TNFRSF11B*	0.35418	POS	down_trans_pathway	corr_trans_hub
ELOVL6	*FASN*	0.8747	POS	down_pathway	down_trans_pathway
ELOVL6	*GNAI1*	0.80139	POS	down_pathway	down_pathway
ELOVL6	*LEP*	0.84565	POS	down_pathway	down_pathway
ELOVL6	*LUM*	0.40036	POS	down_pathway	down_pathway
ELOVL6	*MEST*	0.37539	POS	down_pathway	corr_down_hub
ELOVL6	*MKX*	0.45184	POS	down_pathway	down_TF

ELOVL6	*PCK2*	0.84471	POS	down_pathway	down_pathway
ELOVL6	*PLIN1*	0.7835	POS	down_pathway	down_pathway
ELOVL6	*PRRX2*	0.36095	POS	down_pathway	down_TF
ELOVL6	*RASL11A*	0.1612	POS	down_pathway	corr_RIF
ELOVL6	*RGS7*	0.38384	POS	down_pathway	corr_hub
ELOVL6	*SCD*	0.79431	POS	down_pathway	down_pathway
ELOVL6	*THBS1*	0.56052	POS	down_pathway	down_pathway
ELOVL6	*THBS4*	0.34027	POS	down_pathway	down_trans_pathway
ELOVL6	*TINF2*	-0.30795	NEG	down_pathway	Corr_hub
ELOVL6	*TNC*	0.42476	POS	down_pathway	down_trans_pathway
ELOVL6	*TNFRSF11B*	0.32699	POS	down_pathway	corr_trans_hub
FASN	*GNAI1*	0.71303	POS	down_trans_pathway	down_pathway
FASN	*LEP*	0.80212	POS	down_trans_pathway	down_pathway
FASN	*MKX*	0.40497	POS	down_trans_pathway	down_TF
FASN	*PCK2*	0.87646	POS	down_trans_pathway	down_pathway
FASN	*PLIN1*	0.71819	POS	down_trans_pathway	down_pathway
FASN	*PRRX2*	0.32308	POS	down_trans_pathway	down_TF
FASN	*RASL11A*	0.15733	POS	down_trans_pathway	corr_RIF
FASN	*RGS7*	0.29124	POS	down_trans_pathway	corr_hub
FASN	*SCD*	0.79362	POS	down_trans_pathway	down_pathway
FASN	*THBS1*	0.49089	POS	down_trans_pathway	down_pathway
FASN	*TNFRSF11B*	0.33495	POS	down_trans_pathway	corr_trans_hub
GNAI1	*ITGA10*	0.38036	POS	down_pathway	down_pathway
GNAI1	*LEP*	0.82826	POS	down_pathway	down_pathway
GNAI1	*MEST*	0.37177	POS	down_pathway	corr_down_hub
GNAI1	*MKX*	0.46115	POS	down_pathway	down_TF
GNAI1	*PCK2*	0.79951	POS	down_pathway	down_pathway
GNAI1	*PLIN1*	0.8572	POS	down_pathway	down_pathway
GNAI1	*PRRX2*	0.42283	POS	down_pathway	down_TF
GNAI1	*PTGIR*	0.34158	POS	down_pathway	down_trans_pathway
GNAI1	*RASL11A*	0.17705	POS	down_pathway	corr_RIF
GNAI1	*RGS7*	0.28679	POS	down_pathway	corr_hub
GNAI1	*SCD*	0.66526	POS	down_pathway	down_pathway
GNAI1	*THBS1*	0.60094	POS	down_pathway	down_pathway
GNAI1	*THBS4*	0.3995	POS	down_pathway	down_trans_pathway
GNAI1	*TINF2*	-0.35672	NEG	down_pathway	Corr_hub
GNAI1	*TNC*	0.52323	POS	down_pathway	down_trans_pathway
ITGA10	*LEP*	0.32149	POS	down_pathway	down_pathway
ITGA10	*MEST*	0.36994	POS	down_pathway	corr_down_hub
ITGA10	*MKX*	0.54173	POS	down_pathway	down_TF
ITGA10	*P4HA3*	0.28567	POS	down_pathway	down_pathway
ITGA10	*PRRX2*	0.45121	POS	down_pathway	down_TF
ITGA10	*PTGIR*	0.65128	POS	down_pathway	down_trans_pathway
ITGA10	*THBS1*	0.48917	POS	down_pathway	down_pathway
ITGA10	*THBS4*	0.61115	POS	down_pathway	down_trans_pathway
ITGA10	*TNC*	0.44769	POS	down_pathway	down_trans_pathway
ITGA10	*TNFRSF11B*	0.45768	POS	down_pathway	corr_trans_hub
LEP	*MKX*	0.36353	POS	down_pathway	down_TF
LEP	*PCK2*	0.83492	POS	down_pathway	down_pathway
LEP	*PLIN1*	0.84377	POS	down_pathway	down_pathway
LEP	*PRRX2*	0.38745	POS	down_pathway	down_TF
LEP	*RASL11A*	0.18722	POS	down_pathway	corr_RIF
LEP	*RGS7*	0.37693	POS	down_pathway	corr_hub

LEP	*SCD*	0.71141	POS	down_pathway	down_pathway
LEP	*THBS1*	0.52939	POS	down_pathway	down_pathway
LEP	*TNC*	0.44719	POS	down_pathway	down_trans_pathway
LOC518768	*NUCB2*	-0.29244	NEG	corr_RIF	corr_hub
LOC518768	*P4HA3*	0.18412	POS	corr_RIF	down_pathway
LOC518768	*SCD*	-0.2094	NEG	corr_RIF	down_pathway
LOC530929	*LOC784127*	0.28852	POS	corr_RIF	corr_RIF
LOC530929	*RASL11A*	-0.16597	NEG	corr_RIF	corr_RIF
LOC784127	*MEST*	0.21988	POS	corr_RIF	corr_down_hub
LOC784127	*TNC*	0.21279	POS	corr_RIF	down_trans_pathway
LOC784127	*TNFRSF11B*	0.22071	POS	corr_RIF	corr_trans_hub
LUM	*MEST*	0.61622	POS	down_pathway	corr_down_hub
LUM	*NUCB2*	0.47398	POS	down_pathway	corr_hub
LUM	*SCD*	0.36947	POS	down_pathway	down_pathway
LUM	*SGCE*	0.61025	POS	down_pathway	corr_hub
LUM	*THBS1*	0.42562	POS	down_pathway	down_pathway
LUM	*THBS4*	0.36164	POS	down_pathway	down_trans_pathway
LUM	*TINF2*	-0.50946	NEG	down_pathway	Corr_hub
LUM	*TNC*	0.52155	POS	down_pathway	down_trans_pathway
MEST	*MKX*	0.55959	POS	corr_down_hub	down_TF
MEST	*NUCB2*	0.46304	POS	corr_down_hub	corr_hub
MEST	*PRRX2*	0.41351	POS	corr_down_hub	down_TF
MEST	*PTGIR*	0.53752	POS	corr_down_hub	down_trans_pathway
MEST	*SCD*	0.37172	POS	corr_down_hub	down_pathway
MEST	*SGCE*	0.5298	POS	corr_down_hub	corr_hub
MEST	*THBS1*	0.48128	POS	corr_down_hub	down_pathway
MEST	*THBS4*	0.53978	POS	corr_down_hub	down_trans_pathway
MEST	*TINF2*	-0.48964	NEG	corr_down_hub	Corr_hub
MEST	*TNC*	0.51829	POS	corr_down_hub	down_trans_pathway
MEST	*TNFRSF11B*	0.37144	POS	corr_down_hub	corr_trans_hub
MKX	*NUCB2*	0.46061	POS	down_TF	corr_hub
MKX	*P4HA3*	0.36715	POS	down_TF	down_pathway
MKX	*PCK2*	0.41794	POS	down_TF	down_pathway
MKX	*PLIN1*	0.35278	POS	down_TF	down_pathway
MKX	*PRRX2*	0.62171	POS	down_TF	down_TF
MKX	*PTGIR*	0.6533	POS	down_TF	down_trans_pathway
MKX	*RGS7*	0.32043	POS	down_TF	corr_hub
MKX	*SCD*	0.43326	POS	down_TF	down_pathway
MKX	*SGCE*	0.39757	POS	down_TF	corr_hub
MKX	*THBS1*	0.65482	POS	down_TF	down_pathway
MKX	*THBS4*	0.79032	POS	down_TF	down_trans_pathway
MKX	*TNC*	0.60945	POS	down_TF	down_trans_pathway
MKX	*TNFRSF11B*	0.64234	POS	down_TF	corr_trans_hub
NUCB2	*PRRX2*	0.36496	POS	corr_hub	down_TF
NUCB2	*RGS7*	0.3105	POS	corr_hub	corr_hub
NUCB2	*SGCE*	0.51704	POS	corr_hub	corr_hub
NUCB2	*THBS4*	0.39092	POS	corr_hub	down_trans_pathway
P4HA3	*PRRX2*	0.34638	POS	down_pathway	down_TF
P4HA3	*PTGIR*	0.4154	POS	down_pathway	down_trans_pathway
P4HA3	*THBS1*	0.33243	POS	down_pathway	down_pathway
P4HA3	*THBS4*	0.32541	POS	down_pathway	down_trans_pathway
P4HA3	*TNC*	0.31712	POS	down_pathway	down_trans_pathway
PCK2	*PLIN1*	0.79985	POS	down_pathway	down_pathway

PCK2	*PRRX2*	0.40642	POS	down_pathway	down_TF
PCK2	*RASL11A*	0.18442	POS	down_pathway	corr_RIF
PCK2	*RGS7*	0.28428	POS	down_pathway	corr_hub
PCK2	*SCD*	0.77978	POS	down_pathway	down_pathway
PCK2	*THBS1*	0.5063	POS	down_pathway	down_pathway
PCK2	*THBS4*	0.32985	POS	down_pathway	down_trans_pathway
PCK2	*TNC*	0.40175	POS	down_pathway	down_trans_pathway
PCK2	*TNFRSF11B*	0.30237	POS	down_pathway	corr_trans_hub
PLIN1	*RGS7*	0.27372	POS	down_pathway	corr_hub
PLIN1	*SCD*	0.63613	POS	down_pathway	down_pathway
PLIN1	*THBS1*	0.52817	POS	down_pathway	down_pathway
PLIN1	*TINF2*	-0.30148	NEG	down_pathway	Corr_hub
PLIN1	*TNC*	0.42398	POS	down_pathway	down_trans_pathway
PRRX2	*PTGIR*	0.60269	POS	down_TF	down_trans_pathway
PRRX2	*SGCE*	0.39773	POS	down_TF	corr_hub
PRRX2	*THBS1*	0.46671	POS	down_TF	down_pathway
PRRX2	*THBS4*	0.63399	POS	down_TF	down_trans_pathway
PRRX2	*TNC*	0.64906	POS	down_TF	down_trans_pathway
PRRX2	*TNFRSF11B*	0.43965	POS	down_TF	corr_trans_hub
PTGIR	*THBS1*	0.56709	POS	down_trans_pathway	down_pathway
PTGIR	*THBS4*	0.7446	POS	down_trans_pathway	down_trans_pathway
PTGIR	*TNC*	0.61874	POS	down_trans_pathway	down_trans_pathway
PTGIR	*TNFRSF11B*	0.51947	POS	down_trans_pathway	corr_trans_hub
RASL11A	*THBS1*	0.1995	POS	corr_RIF	down_pathway
RGS7	*SCD*	0.38102	POS	corr_hub	down_pathway
RGS7	*SGCE*	0.24309	POS	corr_hub	corr_hub
RGS7	*THBS1*	0.31645	POS	corr_hub	down_pathway
RGS7	*TNFRSF11B*	0.27191	POS	corr_hub	corr_trans_hub
SCD	*THBS1*	0.44487	POS	down_pathway	down_pathway
SCD	*THBS4*	0.37004	POS	down_pathway	down_trans_pathway
SCD	*TINF2*	-0.28353	NEG	down_pathway	Corr_hub
SGCE	*THBS4*	0.38905	POS	corr_hub	down_trans_pathway
SGCE	*TNC*	0.38196	POS	corr_hub	down_trans_pathway
THBS1	*THBS4*	0.61881	POS	down_pathway	down_trans_pathway
THBS1	*TNC*	0.74819	POS	down_pathway	down_trans_pathway
THBS1	*TNFRSF11B*	0.42755	POS	down_pathway	corr_trans_hub
THBS4	*TNC*	0.61094	POS	down_trans_pathway	down_trans_pathway
THBS4	*TNFRSF11B*	0.53783	POS	down_trans_pathway	corr_trans_hub
TNC	*TNFRSF11B*	0.43997	POS	down_trans_pathway	corr_trans_hub

P

**Origin**	**Target**	**Correlation value**	**Correlation type**	**Origin attributes**	**Target attributes**
ADAM12	bta-miR-130b	0.13915	POS	down_pathways	Corr_miRNA
ADAM12	*CD44*	0.40463	POS	down_pathways	down_pathways
ADAM12	*COL11A1*	0.65195	POS	down_pathways	down_pathways
ADAM12	*COL18A1*	0.39984	POS	down_pathways	down_pathways
ADAM12	*COL21A1*	0.31051	POS	down_pathways	Corr_hub_pathways
ADAM12	*MMP16*	0.48484	POS	down_pathways	Corr_hub_pathways
ADAM12	*THBS4*	0.62637	POS	down_pathways	down_trans_pathways
ADAM12	*TNC*	0.48486	POS	down_pathways	down_trans_pathways
BOLA.DOA	bta-miR-130b	0.16296	POS	Corr_hub	Corr_miRNA

BOLA.DOA	bta-miR-142-5p	0.15143	POS	Corr_hub	Corr_miRNA
BOLA.DOA	bta-miR-92b	0.23373	POS	Corr_hub	Corr_miRNA
BOLA.DOA	*CD44*	0.40579	POS	Corr_hub	down_pathways
BOLA.DOA	*COL21A1*	0.41144	POS	Corr_hub	Corr_hub_pathways
BOLA.DOA	*MMP16*	0.37342	POS	Corr_hub	Corr_hub_pathways
BOLA.DOA	*VDR*	0.30533	POS	Corr_hub	Corr_TF
bta-miR-130b	bta-miR-92b	0.18846	POS	Corr_miRNA	Corr_miRNA
bta-miR-130b	*COL18A1*	0.17597	POS	Corr_miRNA	down_pathways
bta-miR-142-5p	*MMP16*	0.1673	POS	Corr_miRNA	Corr_hub_pathways
bta-miR-142-5p	*PRRX2*	0.22458	POS	Corr_miRNA	down_TF
bta-miR-92b	*CD44*	0.18979	POS	Corr_miRNA	down_pathways
bta-miR-92b	*COL18A1*	0.2622	POS	Corr_miRNA	down_pathways
bta-miR-92b	*MMP16*	0.18661	POS	Corr_miRNA	Corr_hub_pathways
bta-miR-92b	*PRRX2*	0.21628	POS	Corr_miRNA	down_TF
bta-miR-92b	*TNC*	0.21131	POS	Corr_miRNA	down_trans_pathways
CD44	*COL11A1*	0.39695	POS	down_pathways	down_pathways
CD44	*COL18A1*	0.55564	POS	down_pathways	down_pathways
CD44	*COL21A1*	0.40465	POS	down_pathways	Corr_hub_pathways
CD44	*MMP16*	0.54236	POS	down_pathways	Corr_hub_pathways
CD44	*PRRX2*	0.53604	POS	down_pathways	down_TF
CD44	*THBS4*	0.47498	POS	down_pathways	down_trans_pathways
CD44	*TNC*	0.62128	POS	down_pathways	down_trans_pathways
CD44	*VDR*	0.26342	POS	down_pathways	Corr_TF
COL11A1	*MMP16*	0.46702	POS	down_pathways	Corr_hub_pathways
COL11A1	*PRRX2*	0.57971	POS	down_pathways	down_TF
COL11A1	*THBS4*	0.86932	POS	down_pathways	down_trans_pathways
COL11A1	*TNC*	0.5626	POS	down_pathways	down_trans_pathways
COL18A1	*COL21A1*	0.43911	POS	down_pathways	Corr_hub_pathways
COL18A1	*MMP16*	0.49981	POS	down_pathways	Corr_hub_pathways
COL18A1	*PRRX2*	0.59877	POS	down_pathways	down_TF
COL18A1	*THBS4*	0.49388	POS	down_pathways	down_trans_pathways
COL18A1	*TNC*	0.67805	POS	down_pathways	down_trans_pathways
COL18A1	*VDR*	0.28961	POS	down_pathways	Corr_TF
COL21A1	*MMP16*	0.57002	POS	Corr_hub_pathways	Corr_hub_pathways
COL21A1	*VDR*	0.36053	POS	Corr_hub_pathways	Corr_TF
MMP16	*PRRX2*	0.44498	POS	Corr_hub_pathways	down_TF
MMP16	*THBS4*	0.49322	POS	Corr_hub_pathways	down_trans_pathways
MMP16	*TNC*	0.55163	POS	Corr_hub_pathways	down_trans_pathways
MMP16	*VDR*	0.35183	POS	Corr_hub_pathways	Corr_TF
PRRX2	*THBS4*	0.63399	POS	down_TF	down_trans_pathways
PRRX2	*TNC*	0.64906	POS	down_TF	down_trans_pathways
THBS4	*TNC*	0.61094	POS	down_trans_pathways	down_trans_pathways
TNC	*VDR*	0.22886	POS	down_trans_pathways	Corr_TF
VDR	*WDPCP*	0.19512	POS	Corr_TF	Corr_RIF_trans

S

**Origin**	**Target**	**Correlation value**	**Correlation type**	**Origin attributes**	**Target attributes**
ARAP1	*ARHGAP30*	0-51489	POS	Corr_pathways	Corr_pathways
ARAP1	*BTK*	0-38198	POS	Corr_pathways	Corr_pathways
ARAP1	*CCR2*	0-36261	POS	Corr_pathways	Corr_trans_pathways
ARAP1	*CD53*	0-36119	POS	Corr_pathways	Corr_pathways

ARAP1	*CD86*	0-4028	POS	Corr_pathways	Corr_pathways
ARAP1	*FLT3*	0-34789	POS	Corr_pathways	Corr_pathways
ARAP1	*FUT8*	0-33296	POS	Corr_pathways	Corr_RIF_trans
ARAP1	*FYN*	0-38458	POS	Corr_pathways	Corr_pathways
ARAP1	*HIST1H2AC*	-28572	NEG	Corr_pathways	up_pathways
ARAP1	*LOC510860*	0-42819	POS	Corr_pathways	Corr_hub
ARAP1	*LOC534578*	0-32918	POS	Corr_pathways	Corr_pathways
ARAP1	*LPAR4*	0-36227	POS	Corr_pathways	Corr_pathways
ARAP1	*METTL21E*	-30729	NEG	Corr_pathways	Corr_RIF
ARAP1	*PLPPR5*	0-39405	POS	Corr_pathways	Corr_RIF_pathways
ARAP1	*PPT1*	0-42085	POS	Corr_pathways	Corr_hub
ARAP1	*PRRG3*	0-27633	POS	Corr_pathways	Corr_RIF
ARAP1	*TIAM1*	0-36803	POS	Corr_pathways	Corr_trans_pathways
ARAP1	*TNFAIP3*	0-31403	POS	Corr_pathways	Corr_pathways
ARAP1	*VDR*	0-37873	POS	Corr_pathways	Corr_TF
ARAP1	*XCR1*	0-3264	POS	Corr_pathways	Corr_pathways
ARAP1	*XRCC6*	0-27301	POS	Corr_pathways	Corr_pathways
ARHGAP30	*BTK*	0-65664	POS	Corr_pathways	Corr_pathways
ARHGAP30	*CCR2*	0-58851	POS	Corr_pathways	Corr_trans_pathways
ARHGAP30	*CD53*	0-74812	POS	Corr_pathways	Corr_pathways
ARHGAP30	*CD86*	0-66218	POS	Corr_pathways	Corr_pathways
ARHGAP30	*FCGR2A*	0-5613	POS	Corr_pathways	Corr_pathways
ARHGAP30	*FLT3*	0-63256	POS	Corr_pathways	Corr_pathways
ARHGAP30	*FUT8*	0-44991	POS	Corr_pathways	Corr_RIF_trans
ARHGAP30	*FYN*	0-43832	POS	Corr_pathways	Corr_pathways
ARHGAP30	*IKZF3*	0-57856	POS	Corr_pathways	Corr_TF
ARHGAP30	*LOC510860*	0-6192	POS	Corr_pathways	Corr_hub
ARHGAP30	*LOC534578*	0-53657	POS	Corr_pathways	Corr_pathways
ARHGAP30	*PLCB2*	0-53983	POS	Corr_pathways	Corr_trans_pathways
ARHGAP30	*PLPPR5*	0-30923	POS	Corr_pathways	Corr_RIF_pathways
ARHGAP30	*PPT1*	0-62953	POS	Corr_pathways	Corr_hub
ARHGAP30	*SIGLEC5*	0-40913	POS	Corr_pathways	Corr_pathways
ARHGAP30	*TIAM1*	0-50219	POS	Corr_pathways	Corr_trans_pathways
ARHGAP30	*TNFAIP3*	0-45837	POS	Corr_pathways	Corr_pathways
ARHGAP30	*VDR*	0-37471	POS	Corr_pathways	Corr_TF
ARHGAP30	*XCR1*	0-68928	POS	Corr_pathways	Corr_pathways
bta-miR-369-3p	bta-miR-500	0-18148	POS	Corr_RIF_miRNA	Corr_RIF_miRNA
bta-miR-369-3p	*SIGLEC5*	0-26134	POS	Corr_RIF_miRNA	Corr_pathways
bta-miR-500	*FCGR2A*	0-20032	POS	Corr_RIF_miRNA	Corr_pathways
bta-miR-500	*XRCC6*	0-28559	POS	Corr_RIF_miRNA	Corr_pathways
BTK	*CCR2*	0-44616	POS	Corr_pathways	Corr_trans_pathways
BTK	*CD53*	0-62419	POS	Corr_pathways	Corr_pathways
BTK	*CD86*	0-60907	POS	Corr_pathways	Corr_pathways
BTK	*FCGR2A*	0-50601	POS	Corr_pathways	Corr_pathways
BTK	*FLT3*	0-42047	POS	Corr_pathways	Corr_pathways
BTK	*FUT8*	0-43732	POS	Corr_pathways	Corr_RIF_trans
BTK	*FYN*	0-4107	POS	Corr_pathways	Corr_pathways
BTK	*IKZF3*	0-42846	POS	Corr_pathways	Corr_TF
BTK	*LOC510860*	0-57283	POS	Corr_pathways	Corr_hub
BTK	*LOC534578*	0-3371	POS	Corr_pathways	Corr_pathways
BTK	*PLCB2*	0-36076	POS	Corr_pathways	Corr_trans_pathways
BTK	*PPT1*	0-58	POS	Corr_pathways	Corr_hub
BTK	*RAB44*	0-35512	POS	Corr_pathways	Corr_RIF

BTK	*SIGLEC5*	0-35003		POS	Corr_pathways	Corr_pathways
BTK	*TIAM1*	0-54546		POS	Corr_pathways	Corr_trans_pathways
BTK	*TNFAIP3*	0-36622		POS	Corr_pathways	Corr_pathways
BTK	*VDR*	0-35851		POS	Corr_pathways	Corr_TF
BTK	*XCR1*	0-52325		POS	Corr_pathways	Corr_pathways
CCR2	*CD53*	0-55447		POS	Corr_trans_pathways	Corr_pathways
CCR2	*CD86*	0-45495		POS	Corr_trans_pathways	Corr_pathways
CCR2	*FLT3*	0-42323		POS	Corr_trans_pathways	Corr_pathways
CCR2	*FYN*	0-44734		POS	Corr_trans_pathways	Corr_pathways
CCR2	*IKZF3*	0-37517		POS	Corr_trans_pathways	Corr_TF
CCR2	*LOC510860*	0-37379		POS	Corr_trans_pathways	Corr_hub
CCR2	*LOC534578*	0-35087		POS	Corr_trans_pathways	Corr_pathways
CCR2	*PLCB2*	0-42558		POS	Corr_trans_pathways	Corr_trans_pathways
CCR2	*PPT1*	0-55868		POS	Corr_trans_pathways	Corr_hub
CCR2	*SIGLEC5*	0-2855		POS	Corr_trans_pathways	Corr_pathways
CCR2	*TIAM1*	0-332		POS	Corr_trans_pathways	Corr_trans_pathways
CCR2	*TNFAIP3*	0-42224		POS	Corr_trans_pathways	Corr_pathways
CCR2	*XCR1*	0-44147		POS	Corr_trans_pathways	Corr_pathways
CD53	*CD86*	0-75362		POS	Corr_pathways	Corr_pathways
CD53	*FCGR2A*	0-45011		POS	Corr_pathways	Corr_pathways
CD53	*FLT3*	0-61262		POS	Corr_pathways	Corr_pathways
CD53	*FUT8*	0-33628		POS	Corr_pathways	Corr_RIF_trans
CD53	*FYN*	0-46607		POS	Corr_pathways	Corr_pathways
CD53	*HEBP2*	0-32212		POS	Corr_pathways	Corr_pathways
CD53	*IKZF3*	0-61153		POS	Corr_pathways	Corr_TF
CD53	*LOC510860*	0-61968		POS	Corr_pathways	Corr_hub
CD53	*LOC534578*	0-46938		POS	Corr_pathways	Corr_pathways
CD53	*PLCB2*	0-42669		POS	Corr_pathways	Corr_trans_pathways
CD53	*PLPPR5*	0-34334		POS	Corr_pathways	Corr_RIF_pathways
CD53	*PPT1*	0-64597		POS	Corr_pathways	Corr_hub
CD53	*TIAM1*	0-58519		POS	Corr_pathways	Corr_trans_pathways
CD53	*TNFAIP3*	0-45376		POS	Corr_pathways	Corr_pathways
CD53	*VDR*	0-34829		POS	Corr_pathways	Corr_TF
CD53	*XCR1*	0-59794		POS	Corr_pathways	Corr_pathways
CD86	*FCGR2A*	0-40726		POS	Corr_pathways	Corr_pathways
CD86	*FLT3*	0-48374		POS	Corr_pathways	Corr_pathways
CD86	*FUT8*	0-30409		POS	Corr_pathways	Corr_RIF_trans
CD86	*FYN*	0-47325		POS	Corr_pathways	Corr_pathways
CD86	*IKZF3*	0-55656		POS	Corr_pathways	Corr_TF
CD86	*LOC510860*	0-68092		POS	Corr_pathways	Corr_hub
CD86	*LOC534578*	0-49734		POS	Corr_pathways	Corr_pathways
CD86	*PLPPR5*	0-30245		POS	Corr_pathways	Corr_RIF_pathways
CD86	*PPT1*	0-6067		POS	Corr_pathways	Corr_hub
CD86	*RAB44*	0-25889		POS	Corr_pathways	Corr_RIF
CD86	*TIAM1*	0-67847		POS	Corr_pathways	Corr_trans_pathways
CD86	*TNFAIP3*	0-4122		POS	Corr_pathways	Corr_pathways
CD86	*VDR*	0-47296		POS	Corr_pathways	Corr_TF
DAGLB	*LOC510860*		-27898	NEG	Corr_pathways	Corr_hub
DAGLB	*LOC534578*		-32906	NEG	Corr_pathways	Corr_pathways
DAGLB	*PPT1*		-33036	NEG	Corr_pathways	Corr_hub
DAGLB	*XRCC6*		-26885	NEG	Corr_pathways	Corr_pathways
FCGR2A	*FLT3*	0-34253		POS	Corr_pathways	Corr_pathways
FCGR2A	*FUT8*	0-42817		POS	Corr_pathways	Corr_RIF_trans

FCGR2A	*FYN*	0-30979		POS	Corr_pathways	Corr_pathways
FCGR2A	*IKZF3*	0-37653		POS	Corr_pathways	Corr_TF
FCGR2A	*LOC510860*	0-38297		POS	Corr_pathways	Corr_hub
FCGR2A	*LOC534578*	0-37777		POS	Corr_pathways	Corr_pathways
FCGR2A	*METTL21E*		-26636	NEG	Corr_pathways	Corr_RIF
FCGR2A	*PLCB2*	0-37498		POS	Corr_pathways	Corr_trans_pathways
FCGR2A	*PLPPR5*	0-30134		POS	Corr_pathways	Corr_RIF_pathways
FCGR2A	*PPT1*	0-34129		POS	Corr_pathways	Corr_hub
FCGR2A	*RAB44*	0-28343		POS	Corr_pathways	Corr_RIF
FCGR2A	*SIGLEC5*	0-27062		POS	Corr_pathways	Corr_pathways
FCGR2A	*TIAM1*	0-37957		POS	Corr_pathways	Corr_trans_pathways
FCGR2A	*TNFAIP3*	0-31864		POS	Corr_pathways	Corr_pathways
FCGR2A	*XCR1*	0-44347		POS	Corr_pathways	Corr_pathways
FLT3	*IKZF3*	0-54027		POS	Corr_pathways	Corr_TF
FLT3	*LOC510860*	0-51333		POS	Corr_pathways	Corr_hub
FLT3	*PLCB2*	0-42583		POS	Corr_pathways	Corr_trans_pathways
FLT3	*PPT1*	0-44979		POS	Corr_pathways	Corr_hub
FLT3	*PRRG3*	0-2318		POS	Corr_pathways	Corr_RIF
FLT3	*TIAM1*	0-41807		POS	Corr_pathways	Corr_trans_pathways
FLT3	*TNFAIP3*	0-37213		POS	Corr_pathways	Corr_pathways
FLT3	*VDR*	0-34536		POS	Corr_pathways	Corr_TF
FLT3	*XCR1*	0-62696		POS	Corr_pathways	Corr_pathways
FUT8	*HIST1H2AC*		-22617	NEG	Corr_RIF_trans	up_pathways
FUT8	*LOC534578*	0-30702		POS	Corr_RIF_trans	Corr_pathways
FUT8	*PPT1*	0-37802		POS	Corr_RIF_trans	Corr_hub
FUT8	*SIGLEC5*	0-33395		POS	Corr_RIF_trans	Corr_pathways
FUT8	*XCR1*	0-39418		POS	Corr_RIF_trans	Corr_pathways
FYN	*HEBP2*	0-228		POS	Corr_pathways	Corr_pathways
FYN	*IKZF3*	0-29856		POS	Corr_pathways	Corr_TF
FYN	*LOC510860*	0-3443		POS	Corr_pathways	Corr_hub
FYN	*LOC534578*	0-43808		POS	Corr_pathways	Corr_pathways
FYN	*PLPPR5*	0-36632		POS	Corr_pathways	Corr_RIF_pathways
FYN	*PPT1*	0-5906		POS	Corr_pathways	Corr_hub
FYN	*PRRG3*	0-35164		POS	Corr_pathways	Corr_RIF
FYN	*TIAM1*	0-31961		POS	Corr_pathways	Corr_trans_pathways
FYN	*TNFAIP3*	0-31063		POS	Corr_pathways	Corr_pathways
HEBP2	*PLPPR5*	0-26179		POS	Corr_pathways	Corr_RIF_pathways
HEBP2	*PPT1*	0-33621		POS	Corr_pathways	Corr_hub
HIST1H2AC	*TNFAIP3*		-22809	NEG	up_pathways	Corr_pathways
HIST1H2AC	*XRCC6*		-34757	NEG	up_pathways	Corr_pathways
IKZF3	*LOC510860*	0-43366		POS	Corr_TF	Corr_hub
IKZF3	*LOC534578*	0-31628		POS	Corr_TF	Corr_pathways
IKZF3	*PLCB2*	0-37276		POS	Corr_TF	Corr_trans_pathways
IKZF3	*PPT1*	0-42029		POS	Corr_TF	Corr_hub
IKZF3	*TIAM1*	0-50903		POS	Corr_TF	Corr_trans_pathways
IKZF3	*VDR*	0-30084		POS	Corr_TF	Corr_TF
IKZF3	*XCR1*	0-50368		POS	Corr_TF	Corr_pathways
LOC510860	*LOC534578*	0-44349		POS	Corr_hub	Corr_pathways
LOC510860	*LPAR4*	0-28254		POS	Corr_hub	Corr_pathways
LOC510860	*PLPPR5*	0-38647		POS	Corr_hub	Corr_RIF_pathways
LOC510860	*PPT1*	0-57315		POS	Corr_hub	Corr_hub
LOC510860	*RAB44*	0-35388		POS	Corr_hub	Corr_RIF
LOC510860	*SIGLEC5*	0-29684		POS	Corr_hub	Corr_pathways

LOC510860	*TIAM1*	0-50839	POS	Corr_hub	Corr_trans_pathways
LOC510860	*TNFAIP3*	0-34064	POS	Corr_hub	Corr_pathways
LOC510860	*VDR*	0-34286	POS	Corr_hub	Corr_TF
LOC510860	*XRCC6*	0-25681	POS	Corr_hub	Corr_pathways
LOC534578	*METTL21E*	-2926	NEG	Corr_pathways	Corr_RIF
LOC534578	*PPT1*	0-50389	POS	Corr_pathways	Corr_hub
LOC534578	*PRRG3*	0-28013	POS	Corr_pathways	Corr_RIF
LOC534578	*VDR*	0-38362	POS	Corr_pathways	Corr_TF
LOC534578	*XCR1*	0-34365	POS	Corr_pathways	Corr_pathways
LPAR4	*PLPPR5*	0-30937	POS	Corr_pathways	Corr_RIF_pathways
LPAR4	*RAB44*	0-31385	POS	Corr_pathways	Corr_RIF
METTL21E	*PRRG3*	-26111	NEG	Corr_RIF	Corr_RIF
METTL21E	*VDR*	-2346	NEG	Corr_RIF	Corr_TF
PLCB2	*PPT1*	0-43394	POS	Corr_trans_pathways	Corr_hub
PLCB2	*TNFAIP3*	0-35565	POS	Corr_trans_pathways	Corr_pathways
PLCB2	*XCR1*	0-54544	POS	Corr_trans_pathways	Corr_pathways
PLPPR5	*PPT1*	0-3894	POS	Corr_RIF_pathways	Corr_hub
PLPPR5	*PRRG3*	0-27899	POS	Corr_RIF_pathways	Corr_RIF
PLPPR5	*RAB44*	0-3015	POS	Corr_RIF_pathways	Corr_RIF
PLPPR5	*XRCC6*	0-24823	POS	Corr_RIF_pathways	Corr_pathways
PPT1	*PRRG3*	0-29195	POS	Corr_hub	Corr_RIF
PPT1	*SIGLEC5*	0-31909	POS	Corr_hub	Corr_pathways
PPT1	*TNFAIP3*	0-37246	POS	Corr_hub	Corr_pathways
PPT1	*VDR*	0-34204	POS	Corr_hub	Corr_TF
PPT1	*XCR1*	0-58532	POS	Corr_hub	Corr_pathways
PPT1	*XRCC6*	0-27682	POS	Corr_hub	Corr_pathways
PRRG3	*XCR1*	0-26394	POS	Corr_RIF	Corr_pathways
RAB44	*TIAM1*	0-35877	POS	Corr_RIF	Corr_trans_pathways
SIGLEC5	*XCR1*	0-30233	POS	Corr_pathways	Corr_pathways
TIAM1	*TNFAIP3*	0-374	POS	Corr_trans_pathways	Corr_pathways
TIAM1	*VDR*	0-3994	POS	Corr_trans_pathways	Corr_TF
TIAM1	*XCR1*	0-35556	POS	Corr_trans_pathways	Corr_pathways
TNFAIP3	*VDR*	0-29242	POS	Corr_pathways	Corr_TF
TNFAIP3	*XCR1*	0-39622	POS	Corr_pathways	Corr_pathways

Supplementary Table S4. Correlations and attributes of each significant correlation constituting Figure 4

Zn

Origin	Target	Correlation value	Correlation type	Origin attributes	Target attributes
AAR2	*CTSD*	0.28108	POS	Corr	Corr
AAR2	*MBTPS2*	-0.24427	NEG	Corr	Corr_RIF
AAR2	*NUDT18*	0.34448	POS	Corr	Corr_RIF_trans
AKAP9	*CTSD*	-0.43766	NEG	up	Corr
AKAP9	*MBTPS2*	0.50854	POS	up	Corr_RIF
AKAP9	*NUDT18*	-0.31265	NEG	up	Corr_RIF_trans
ANGPTL2	*LTV1*	-0.42055	NEG	Corr	Corr
ANGPTL2	*TNR*	0.33011	POS	Corr	Corr_RIF
ANGPTL2	*ZNF770*	-0.3445	NEG	Corr	Corr
ANGPTL4	*CTSD*	0.29498	POS	down	Corr
ASF1B	*CTSD*	0.24898	POS	Corr	Corr
ASF1B	*GABPB1*	-0.28056	NEG	Corr	Corr
ASF1B	*LTV1*	-0.33302	NEG	Corr	Corr
ASF1B	*MBTPS2*	-0.25637	NEG	Corr	Corr_RIF
ASF1B	*TNR*	0.26734	POS	Corr	Corr_RIF
BANK1	bta-miR-199b	-0.20456	NEG	up	Corr_RIF_miRNA
bta-miR-142-5p	*NOX1*	-0.16718	NEG	Corr_miRNA	Corr_RIF
bta-miR-142-5p	*TNR*	0.18856	POS	Corr_miRNA	Corr_RIF
bta-miR-199b	bta-miR-2285bl	0.48295	POS	Corr_RIF_miRNA	Corr_miRNA
bta-miR-199b	bta-miR-2285co	0.48295	POS	Corr_RIF_miRNA	Corr_miRNA
bta-miR-199b	bta-miR-411c-5p	0.33691	POS	Corr_RIF_miRNA	Corr_RIF_miRNA
bta-miR-199b	*CLDN5*	0.20895	POS	Corr_RIF_miRNA	down
bta-miR-199b	*CYGB*	0.22007	POS	Corr_RIF_miRNA	down
bta-miR-199b	*GABPB1*	-0.20605	NEG	Corr_RIF_miRNA	Corr
bta-miR-199b	*GABPB1*	-0.20605	NEG	Corr_RIF_miRNA	Corr
bta-miR-199b	*HIST1H2AC*	-0.1978	NEG	Corr_RIF_miRNA	up
bta-miR-199b	*LTV1*	-0.23378	NEG	Corr_RIF_miRNA	Corr
bta-miR-199b	*LTV1*	-0.23378	NEG	Corr_RIF_miRNA	Corr
bta-miR-199b	*MBTPS2*	-0.22852	NEG	Corr_RIF_miRNA	Corr_RIF
bta-miR-199b	*MBTPS2*	-0.22852	NEG	Corr_RIF_miRNA	Corr_RIF
bta-miR-199b	*MIR29E*	-0.21675	NEG	Corr_RIF_miRNA	Corr_miRNA
bta-miR-199b	*ZCCHC10*	-0.2059	NEG	Corr_RIF_miRNA	Corr
bta-miR-199b	*ZNF770*	-0.26387	NEG	Corr_RIF_miRNA	Corr
bta-miR-199b	*ZNF770*	-0.26387	NEG	Corr_RIF_miRNA	Corr
bta-miR-2285bl	bta-miR-411c-5p	0.32726	POS	Corr_miRNA	Corr_RIF_miRNA
bta-miR-2285bl	*NOX1*	-0.21825	NEG	Corr_miRNA	Corr_RIF
bta-miR-2285bl	*ZNF770*	-0.22403	NEG	Corr_miRNA	Corr
bta-miR-2285co	bta-miR-411c-5p	0.32726	POS	Corr_miRNA	Corr_RIF_miRNA
bta-miR-2285co	*NOX1*	-0.21825	NEG	Corr_miRNA	Corr_RIF
bta-miR-2285co	*ZNF770*	-0.22403	NEG	Corr_miRNA	Corr
bta-miR-411c-5p	bta-miR-425-3p	-0.15359	NEG	Corr_RIF_miRNA	Corr_miRNA
bta-miR-411c-5p	*C1QTNF1*	0.24445	POS	Corr_RIF_miRNA	down
bta-miR-411c-5p	*HIST1H2AC*	-0.22101	NEG	Corr_RIF_miRNA	up
bta-miR-411c-5p	*IFI6*	0.1738	POS	Corr_RIF_miRNA	up
bta-miR-411c-5p	*LOC514189*	-0.2206	NEG	Corr_RIF_miRNA	Corr

bta-miR-411c-5p	*LTV1*	-0.23098	NEG	Corr_RIF_miRNA	Corr
bta-miR-411c-5p	*LTV1*	-0.23098	NEG	Corr_RIF_miRNA	Corr
bta-miR-411c-5p	*NUDT18*	0.19876	POS	Corr_RIF_miRNA	Corr_RIF_trans
bta-miR-411c-5p	*ZNF770*	-0.26779	NEG	Corr_RIF_miRNA	Corr
bta-miR-411c-5p	*ZNF770*	-0.26779	NEG	Corr_RIF_miRNA	Corr
bta-miR-500	*LTV1*	-0.2087	NEG	Corr_miRNA	Corr
C1QTNF1	*LTV1*	-0.32425	NEG	down	Corr
C1QTNF1	*MBTPS2*	-0.33074	NEG	down	Corr_RIF
C1QTNF1	*ZNF770*	-0.41717	NEG	down	Corr
C7H19orf67	*ZNF770*	-0.27928	NEG	Corr	Corr
CHPT1	*MBTPS2*	0.27574	POS	Corr_ASE	Corr_RIF
CHPT1	*ZNF770*	0.24672	POS	Corr_ASE	Corr
CISH	*GABPB1*	0.31863	POS	up	Corr
CLDN5	*LTV1*	-0.34565	NEG	down_trans	Corr
CLDN5	*MBTPS2*	-0.32143	NEG	down_trans	Corr_RIF
COL11A2	*CTSD*	0.17185	POS	Corr_trans	Corr
CTSD	*GID8*	0.46674	POS	Corr	Corr
CTSD	*GRM4*	0.27252	POS	Corr	Corr
CTSD	*LOC112443416*	0.24901	POS	Corr	Corr
CTSD	*LOC514189*	-0.29473	NEG	Corr	Corr
CTSD	*LOC613985*	0.26814	POS	Corr	Corr
CTSD	*LOC786948*	0.33692	POS	Corr	down
CTSD	*LTV1*	-0.52707	NEG	Corr	Corr
CTSD	*MBTPS2*	-0.4495	NEG	Corr	Corr_RIF
CTSD	*MBTPS2*	-0.4495	NEG	Corr	Corr_RIF
CTSD	*MGLL*	0.5637	POS	Corr	down
CTSD	*MIR133A.2*	-0.26866	NEG	Corr	Corr_miRNA
CTSD	*NBN*	-0.35234	NEG	Corr	Corr
CTSD	*NUDT18*	0.32112	POS	Corr	Corr_RIF_trans
CTSD	*NUDT18*	0.32112	POS	Corr	Corr_RIF_trans
CTSD	*PON3*	-0.40338	NEG	Corr	up
CTSD	*RGMA*	0.40336	POS	Corr	Corr
CTSD	*ROCK2*	-0.30379	NEG	Corr	up_ASE
CTSD	*TTC9*	0.35054	POS	Corr	Corr
CTSD	*VPS18*	0.37271	POS	Corr	Corr
CTSD	*ZNF770*	-0.30763	NEG	Corr	Corr
CYGB	*NOX1*	-0.20447	NEG	down	Corr_RIF
DCX	*ZNF770*	-0.22009	NEG	Corr	Corr
FAIM	*GABPB1*	0.30886	POS	Corr	Corr
FAIM	*MBTPS2*	0.22133	POS	Corr	Corr_RIF
GABPB1	*GID8*	-0.33334	NEG	Corr	Corr
GABPB1	*GRM4*	-0.28547	NEG	Corr	Corr
GABPB1	*HIST1H2AC*	0.31302	POS	Corr	up
GABPB1	*INSIG2*	0.32392	POS	Corr	Corr_trans
GABPB1	*LEMD3*	0.31656	POS	Corr	Corr
GABPB1	*LOC107132969*	-0.26644	NEG	Corr	Corr
GABPB1	*LOC112446096*	-0.21757	NEG	Corr	Corr
GABPB1	*LOC613985*	-0.31462	NEG	Corr	Corr
GABPB1	*LTV1*	0.32642	POS	Corr	Corr
GABPB1	*NBN*	0.29468	POS	Corr	Corr

GABPB1	*NOX1*	0.42009	POS	Corr	Corr_RIF
GABPB1	*NOX1*	0.42009	POS	Corr	Corr_RIF
GABPB1	*RGMA*	-0.26288	NEG	Corr	Corr
GABPB1	*ROCK2*	0.25672	POS	Corr	up_ASE
GABPB1	*SAT2*	-0.26351	NEG	Corr	Corr
GABPB1	*TTC9*	-0.25702	NEG	Corr	Corr
GABPB1	*VPS18*	-0.42129	NEG	Corr	Corr
GID8	*LTV1*	-0.30295	NEG	Corr	Corr
GID8	*NOX1*	-0.2938	NEG	Corr	Corr_RIF
GID8	*NUDT18*	0.27679	POS	Corr	Corr_RIF_trans
GRM4	*NUDT18*	0.2405	POS	Corr	Corr_RIF_trans
HSPA6	*TNR*	-0.19207	NEG	down	Corr_RIF
INSIG2	*LTV1*	0.26483	POS	Corr_trans	Corr
INSIG2	*NOX1*	0.42979	POS	Corr_trans	Corr_RIF
LEMD3	*MBTPS2*	0.32064	POS	Corr	Corr_RIF
LEMD3	*NOX1*	0.28393	POS	Corr	Corr_RIF
LEMD3	*NUDT18*	-0.27272	NEG	Corr	Corr_RIF_trans
LEMD3	*ZNF770*	0.32174	POS	Corr	Corr
LEP	*ZNF770*	-0.26784	NEG	up	Corr
LOC112443416	*MBTPS2*	-0.25125	NEG	Corr	Corr_RIF
LOC112443416	*ZNF770*	-0.21988	NEG	Corr	Corr
LOC112446096	*NOX1*	-0.18781	NEG	Corr	Corr_RIF
LOC514189	*LTV1*	0.32039	POS	Corr	Corr
LOC514189	*MBTPS2*	0.28909	POS	Corr	Corr_RIF
LOC514189	*ZNF770*	0.26242	POS	Corr	Corr
LOC613985	*NUDT18*	0.37017	POS	Corr	Corr_RIF_trans
LOC617875	*NUDT18*	0.21105	POS	Corr	Corr_RIF_trans
LOC786948	*LTV1*	-0.3489	NEG	down	Corr
LOC786948	*MBTPS2*	-0.39048	NEG	down	Corr_RIF
LOC786948	*ZNF770*	-0.27263	NEG	down	Corr
LTV1	*MBTPS2*	0.48598	POS	Corr	Corr_RIF
LTV1	*MBTPS2*	0.48598	POS	Corr	Corr_RIF
LTV1	*MGLL*	-0.38026	NEG	Corr	down
LTV1	*MIR133A.2*	0.34694	POS	Corr	Corr_miRNA
LTV1	*MIR29E*	0.30578	POS	Corr	Corr_miRNA
LTV1	*MPZ*	-0.17809	NEG	Corr	down
LTV1	*RGMA*	-0.46768	NEG	Corr	Corr
LTV1	*ROCK2*	0.33819	POS	Corr	up_ASE
LTV1	*TTC9*	-0.31801	NEG	Corr	Corr
LTV1	*VPS18*	-0.43152	NEG	Corr	Corr
LTV1	*ZIC3*	0.25538	POS	Corr	Corr_TF
MBTPS2	*MGLL*	-0.37126	NEG	Corr_RIF	down
MBTPS2	*MIR29E*	0.30704	POS	Corr_RIF	Corr_miRNA
MBTPS2	*MX1*	0.22051	POS	Corr_RIF	up
MBTPS2	*NUDT18*	-0.26507	NEG	Corr_RIF	Corr_RIF_trans
MBTPS2	*NUDT18*	-0.26507	NEG	Corr_RIF	Corr_RIF_trans
MBTPS2	*ROCK2*	0.42649	POS	Corr_RIF	up_ASE
MBTPS2	*SAT2*	-0.48258	NEG	Corr_RIF	Corr
MBTPS2	*TTC9*	-0.2767	NEG	Corr_RIF	Corr
MBTPS2	*ZCCHC10*	0.4179	POS	Corr_RIF	Corr
MBTPS2	*ZNF770*	0.55736	POS	Corr_RIF	Corr
MBTPS2	*ZNF770*	0.55736	POS	Corr_RIF	Corr
MGLL	*NUDT18*	0.35164	POS	down	Corr_RIF_trans

MX1	*TNR*	0.1198	POS	up	Corr_RIF
NOX1	*NUDT18*	-0.22951	NEG	Corr_RIF	Corr_RIF_trans
NOX1	*UCP2*	-0.2164	NEG	Corr_RIF	down
NUDT18	*SAT2*	0.28157	POS	Corr_RIF_trans	Corr
RGMA	*TNR*	0.34056	POS	Corr	Corr_RIF
ROCK2	*ZNF770*	0.45341	POS	up_ASE	Corr
SAT2	*ZNF770*	-0.34911	NEG	Corr	Corr
ZCCHC10	*ZNF770*	0.43701	POS	Corr	Corr
ZIC3	*ZNF770*	0.26069	POS	Corr_TF	Corr
